# Cellular Immune Responses Induced with Dose-Sparing Intradermal Administration of HIV Vaccine to HIV-Uninfected Volunteers in the ANRS VAC16 Trial

**DOI:** 10.1371/journal.pone.0000725

**Published:** 2007-08-22

**Authors:** Odile Launay, Christine Durier, Corinne Desaint, Benjamin Silbermann, Angela Jackson, Gilles Pialoux, Bénédicte Bonnet, Isabelle Poizot-Martin, Gustavo Gonzalez-Canali, Lise Cuzin, Suzanne Figuereido, Mathieu Surenaud, Nadine Ben Hamouda, Hanne Gahery, Jeannine Choppin, Dominique Salmon, Corinne Guérin, Isabelle Bourgault Villada, Jean-Gérard Guillet, for the ANRS VAC16 Study Group

**Affiliations:** 1 Université Paris Descartes, Faculté de Médecine, Paris, France; 2 Assistance Publique–Hôpitaux de Paris, Groupe Hospitalier Cochin-Saint Vincent de Paul, Pôle de Médecine, CIC de Vaccinologie Cochin-Pasteur, Paris, France; 3 INSERM, CIC de Vaccinologie Cochin-Pasteur, Paris, France; 4 INSERM SC10, Villejuif, France; 5 Institut Cochin, Université Paris Descartes, CNRS UMR 8104, Paris, France; 6 INSERM, U567, Paris, France; 7 AP–HP, Hôpital Tenon, Service des Maladies Infectieuses, Paris, France; 8 CHU Hôtel Dieu, Service des Maladies Infectieuses, Nantes, France; 9 Hôpital Sainte-Marguerite, Unité CISIH Sud Hématologie VIH, Marseille, France; 10 Assistance Publique–Hôpitaux de Paris, Hôpital Européen Georges Pompidou, Service d'Immunologie Clinique, Paris, France; 11 Hôpital Purpan, Service des Maladies Infectieuses, Toulouse, France; 12 Assistance Publique–Hôpitaux de Paris, Hôpital Cochin, Pharmacie, Paris, France; 13 Assistance Publique–Hôpitaux de Paris, Hôpital Ambroise Paré, Service de Dermatologie, Boulogne, France; University of Maryland School of Medicine, United States of America

## Abstract

**Objective:**

The objective was to compare the safety and cellular immunogenicity of intradermal versus intramuscular immunization with an HIV-lipopeptide candidate vaccine (LIPO-4) in healthy volunteers.

**Methodology:**

A randomized, open-label trial with 24 weeks of follow-up was conducted in France at six HIV-vaccine trial sites. Sixty-eight healthy 21– to 55–year-old HIV-uninfected subjects were randomized to receive the LIPO-4 vaccine (four HIV lipopeptides linked to a T-helper–stimulating epitope of tetanus-toxin protein) at weeks 0, 4 and 12, either intradermally (0.1 ml, 100 µg of each peptide) or intramuscularly (0.5 ml, 500 µg of each peptide). Comparative safety of both routes was evaluated. CD8^+ ^T-cell immune responses to HIV epitopes (ELISpot interferon-γ assay) and tetanus toxin-specific CD4^+ ^T-cell responses (lymphoproliferation) were assessed at baseline, two weeks after each injection, and at week 24.

**Results and Conclusion:**

No severe, serious or life-threatening adverse events were observed. Local pain was significantly more frequent after intramuscular injection, but local inflammatory reactions were more frequent after intradermal immunization. At weeks 2, 6, 14 and 24, the respective cumulative percentages of induced CD8^+ ^T-cell responses to at least one HIV peptide were 9, 33, 39 and 52 (intradermal group) or 14, 20, 26 and 37 (intramuscular group), and induced tetanus toxin-specific CD4^+ ^T-cell responses were 6, 27, 33 and 39 (intradermal), or 9, 46, 54 and 63 (intramuscular). In conclusion, intradermal LIPO-4 immunization was well tolerated, required one-fifth of the intramuscular dose, and induced similar HIV-specific CD8^+ ^T-cell responses. Moreover, the immunization route influenced which antigen-specific T-cells (CD4^+^ or CD8^+^) were induced.

**Trial Registration:**

ClinicalTrials.gov NCT00121121

## Introduction

One of the greatest challenges in human immunodeficiency virus/acquired immunodeficiency syndrome (HIV/AIDS) research is to develop a vaccine that can prevent virus transmission or halt progression to AIDS. An effective HIV vaccine should induce neutralizing antibodies to protect against infection [1]. However, inducing neutralizing antibodies specific to the broad range of HIV subspecies has proven difficult with current candidate vaccines. Alternatively, numerous clinical and experimental observations have shown that cellular immunity, particularly CD8^+^ T lymphocytes, plays an important role in controlling HIV infection. These findings led researchers to develop vaccines able to generate HIV-specific cellular responses. Using HIV peptides covalently linked to a lipid tail, an epitope-based candidate vaccine was found to be safe and was able to elicit HIV-specific CD4^+^ and CD8^+^ T-cell responses [2]. Lipopeptide formulations successfully induced antiviral cytotoxic T-lymphocyte (CTL) responses in mice [3]–[7] and monkeys [8]–[10], and hepatitis B virus (HBV)-specific CTL in humans [11], [12]. More recent studies showed that intramuscularly injected HIV lipopeptides were able to trigger HIV-specific T-cell responses in HIV-uninfected volunteers [13]–[15] and HIV-infected patients [16]–[17]. The lipid moiety facilitates the peptide's entry into antigen-presenting dendritic cells, thereby enhancing cell-mediated immune responses [18]–[20].

Intradermal administration is expected to enhance antigen exposure to antigen-presenting cells, because skin harbors more macrophages and dendritic cells than muscle. Those cells incorporate antigens and migrate to draining lymph nodes to present antigen fragments to resting T lymphocytes, thereby initiating antigen-specific immune responses. Thus, the skin is an attractive site for vaccine delivery, achieving the most effective immunization with the smallest antigen load. Indeed, studies on intradermal injection of HBV, rabies and, more recently, influenza vaccines highlighted the potential of this route in improving immunogenicity [21]–[25]. However, most of those studies evaluated only humoral immune responses; very few examined whether that route could induce cellular immune responses more efficiently than intramuscular injection [26]. Moreover, to our knowledge, no studies have been conducted in humans to determine whether the immunization route influences the nature of the antigen-specific CD4^+^ or CD8^+^ T cell stimulation. Our preclinical study results showed that intradermal administration of simian immunodeficiency virus (SIV) lipopeptides triggered multispecific and sustained SIV-specific T-cell responses in rhesus macaques [27]. Intradermal injection of HIV lipopeptides might be used to induce a more favorable immune response. Also, whether intradermal injection of a fraction of the HIV-lipopeptide vaccine dose was proven to be as immunogenic as the intramuscular full dose, it would be a valid dose-sparing strategy. Encouraged by the results obtained with intradermal lipopeptide immunization in the SIV–macaque model, we evaluated the safety and cellular immunogenicity of intradermal injection of one-fifth the intramuscular dose of the HIV-lipopeptide candidate vaccine (LIPO-4) [28], [29] in a prospective, randomized trial in HIV-uninfected adult volunteers.

## Methods

The protocol for this trial and supporting CONSORT checklist are available as supporting information; see Checklist S1 and Protocol S1.

### Study design

This multicenter, open-label, randomized, phase I-B trial was conducted at six HIV-vaccine trial sites in France. The protocol was reviewed and approved by the Pitié–Salpêtrière Hospital Ethics Committee (Paris, France) and all volunteers gave informed consent.

### Participants

HIV-1-uninfected healthy volunteers, 21 through 55 years of age, were pre-screened according to the procedure established by the French National Agency for AIDS Research (ANRS) to select HIV-unexposed healthy volunteers presenting psychological and sociological stability, i.e. able to be followed and with no plan to move during the entire trial. The selection procedure consisted in medical and psychological exams and standard laboratory tests. Subjects with a pre-existing medical condition or abnormal laboratory values that could affect the standard or protocol safety or immunogenicity evaluations were excluded. HIV-negative status was confirmed by both ELISA and Western blot tests. A committee composed of physicians specialized in clinical immunology, internal medicine, infectious diseases, psychiatrists and a sociologist accepted or not the candidate in the “ANRS HIV-uninfected volunteers network”. They were counseled regarding the uncertainty of efficacy of the trial vaccine and the need to avoid HIV infection and they did not receive financial incentive. Women were required to use effective contraception and have a negative pregnancy test prior to each vaccine injection.

### Interventions

Subjects were randomized to receive, at weeks 0, 4 and 12, the LIPO-4 vaccine injected intradermally (0.1 ml) or intramuscularly (0.5 ml) into the deltoid region of the nondominant arm. Solicited adverse events included the following local and systemic reactions: pain, erythema, induration, nodule, vesicle, papule, bulla and/or edema at the injection site; fever (at least 37.5°C), headache, malaise, nausea, diarrhea, rash, myalgia and/or arthralgia. Participants were seen 3 days and 2 weeks after each vaccination for safety assessment. Vaccination-site induration was measured 3 days postinjection. Information on adverse events was collected throughout the trial. Adverse events were codified for their relationship to the study product (none, possibly, probably or certainly related) and assigned a severity grade. Immune responses were assessed at weeks 0 (before injection), 2, 6, 14 and 24.

### Study Vaccine

**Figure 1:**
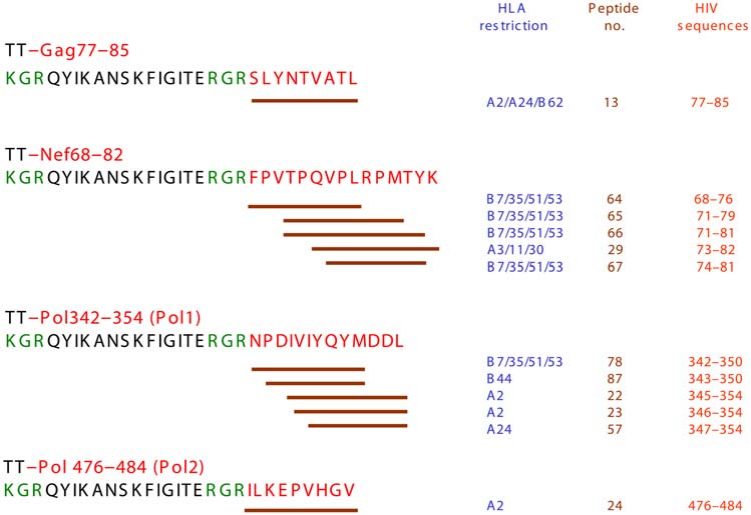
Composition of the LIPO-4 vaccine. The amino acid sequences correspond to the four antigenic segments of HIV proteins (red) covalently linked to the T-helper–stimulating epitope of tetanus toxin (TT, black) and linker residues (green). The lines under the HIV sequences represent 12 previously reported CD8^+^ T-cell–stimulating epitopes (peptide numbers in brown); the HLA class-I molecules (A or B in blue) restricting the T-cell responses are also indicated.

LIPO-4 vaccine contains four HIV sequences (Gag77–85, Pol342–354, Pol476–484 and Nef68–82), covalently linked to a T-helper–stimulating tetanus toxin (TT) peptide, TT830–843 (Figure 1). To synthesize these monopalmitoyl peptides, a Boc–Lys–(Fmoc)–OH group was incorporated at the N-terminal. Next, on-resin palmitylation was performed as previously described [30], as were purification, solubilization and formulation (mixed micelles) of the lipopeptide vaccines [2]. Each lipopeptide was synthesized by Avecia LifeScience Molecules (Northwich, England) under GMP conditions, purified to ≥90%, with identity controlled by amino-acid–composition analysis after total acid hydrolysis and molecular mass determination. The final formulation was subjected to sterilizing filtration under GMP conditions (Sterilyo, Saint-Amand-les-Eaux, France), and distributed into individual vials, lyophilized and stored under nitrogen. Each vaccine dose was a lyophilized mixture of the four lipopeptides that was reconstituted with 0.5 ml of a 5% isotonic glucose solution and injected either intradermally (0.1 ml, 100 µg of each peptide) or intramuscularly (0.5 ml, 500 µg of each peptide).

### Objectives

The primary objective was to determine whether intradermal versus intramuscular LIPO-4 vaccine injection resulted in significantly different percentages of participants experiencing any grade 2 or higher adverse event, possibly, probably or certainly related to the vaccination. Secondary endpoints were the evaluation of T-cell immune responses.

### Outcomes

Safety Assessment. All subjects were observed for 30 minutes following vaccine administration to check for immediate local and/or systemic reactions. A self-monitoring diary card was given to all subjects to record any local and/or systemic reaction occurring during the post-injection period and any adverse event occurring during the entire study period. Clinical evaluations were performed at days 3 and 14 post-injection, where the study staff reviewed the diary card and followed up on any adverse event that had occurred since injection. All local and systemic adverse events were recorded, regardless of severity. The adverse event severity-grading scale was defined as follows: mild (grade 1) with no limitation of activities and no medical intervention; moderate (grade 2) with mild-to-moderate limitation of activities and no or minimal medical intervention; severe (grade 3) with marked limitation of activities and medical intervention required; and potentially life-threatening (grade 4). Local symptoms occurring at injection-site (e.g., erythema, induration or edema) were categorized as grade 1 if <15×15 cm; grade 2 if ≥15×15 cm; grade 3 for ulceration, superinfection or superficial phlebitis; and grade 4 for skin necrosis . Physicians graded severity, using self-monitoring diary cards filled by the volunteer. All events were coded in MedDRA terms and then linked to MedDRA system-organ class and preferred terms for reporting. The Events Validation Committee reviewed all events occurring during the study for severity and relationship to LIPO-4. Stopping rules were prespecified for safety concerns, such as grade 3 or 4 adverse events.

### Assessment of Immunogenicity

#### Long and Short Peptides

Short overlapping peptides spanning the four lipopeptide sequences and known to be optimal CD8^+^ T-lymphocyte–stimulating epitopes were synthesized by NeoMPS (Strasbourg, France) and tested in ELISpot interferon-γ (IFNγ) assays. The compositions of the 12 peptides used are shown in Figure 1. These sequences cover the range of HLA class-I alleles expressed by volunteers. The TT peptide (QYIKANSKFIGITELKK) known as a helper epitope was used in proliferation assays.

### IFNγ Secretion by CD8^+^ T Cells Detected by ELISpot Assay

A one-step stimulation strategy (cultured ELISpot) was used to amplify the CD8^+^ T-cell responses before ELISpot assay [14]. Briefly, 4×10^6^ fresh peripheral blood mononuclear cells (PBMC) were incubated for 2 hours at 37°C in complete medium (RPMI-1640 supplemented with 10% human AB serum) containing a maximum of six CD8^+^ T-cell–stimulating epitopes (2 µg of each/ml). PBMC collected before and after each vaccination were tested for their abilities to secrete IFNγ in response to several HLA class I-restricted peptides. All the HIV-1 epitopes contained in LIPO-4 were tested independently of the volunteer's HLA haplotype. A positive control, including peptides derived from Epstein-Barr virus (EBV), influenza virus and cytomegalovirus (CMV) was also used. Positive spots were detected using an AID ELISpot reader system (AID Autoimmun Diagnostika, Strassberg, Germany). Negative controls were PBMC incubated in medium alone. To be assessable, a given cell line had to have one positive pool of viral peptides (CMV, EBV, influenza virus), or, if none of the viral peptides generated positive spots for a given subject, the phytohemagglutinin (PHA; 1 µg/ml; 25,000 cells/well) response had to be positive. The test of an HIV or viral pool was assessable when the mean of triplicate wells exceeded 100 spot-forming units (SFU)/10^6^ PBMC. The positive ELISpot threshold was the mean of triplicate wells three-fold higher than the negative control. The response to HIV peptides was never the only positive finding. Peptide-specific T-cell responses were not considered positive if they were detected before vaccination.

### Anti-Tetanus Toxin Peptide-Induced CD4^+^ T-Cell Proliferation

The proliferation of each subject's fresh PBMC during 7 days of incubation in complete medium with phytohemagglutinin (PHA; 1 µg/ml), purified protein derivative (1 µg/ml), TT peptide (1 µg/ml) and staphylococcal enterotoxin B (0.1 µg/ml) was determined. [^3^H]Thymidine uptake was expressed as a stimulation index: mean test counts/minute (cpm) of quadruplicate wells/mean background cpm. An index ≥3 was considered positive.

Due to their structure, lipopeptides are known to induce a CD8^+ ^T-cell response, although they also elicit a CD4^+ ^T-cell response [9], [13]. The frequency and duration of the CD8^+ ^T-cell response are influenced by the presence of CD4^+ ^T-cell response. In the present work, both CD8 (against short HIV peptides) and CD4 (against TT830-843) response were investigated.

### Sample size

The null hypothesis was that the intradermal and intramuscular routes would each have a 45% possibly, probably or certainly vaccination-related grade 2 or higher adverse event rate, excluding local pain, i.e., that observed in an ANRS pilot trial using LIPO-4 vaccine (unpublished data). To detect a between-arm difference of 45 versus 80% in a two group continuity corrected chi square test with 80% power and a two-sided significance level of 0.05, 35 patients per arm suffice.

### Randomization—Sequence generation

Randomization was stratified by the participant's HLA-type, able to induce CD8 immune responses against 0–1 peptide, 2–5 peptides or 6–11 peptides, respectively. Randomization lists were generated with a block size of 4 using the statistical program SAS (SAS system for Windows version 8; SAS, Cary, North Carolina, United States). The three lists were generated by the trial statistician, who had no involvement in enrolment, follow-up, or assessment of participants.

### Randomization—Allocation Concealment

Individuals were randomized by computer-generated lists, which were maintained centrally so no center knew the treatment allocation of any participant prior to randomization.

### Randomization—Implementation

A central coordinating office was responsible for validation of participant eligibility, randomization, data collection, and monitoring. Once the screening process was completed, and in order of enrolment, each participant was assigned to the intradermal or intramuscular route by a centralized process, according to computer-generated random lists. The result of individual randomization was faxed and e-mailed by the central office to the trial center.

### Blinding

In this open-label study regarding safety assessment, all laboratory analyses were performed by individuals blinded to administration route assignments. Clinical staff and volunteers were aware of route assignments.

### Statistical Analysis

The primary outcome was the proportion of subjects experiencing any grade 2 adverse event possibly, probably or certainly vaccination-related, injection-site pain excluded (as specified in the protocol). The proportion including injection-site pain was also analyzed. CD4^+^ T-cell proliferation and CD8^+^ T-cell IFNγ secretion were analyzed based on a priori assessable assays at each time, independently of the vaccination route. Primary and secondary endpoints were analyzed based on intention-to-treat (using the same definitions of assessable as for immunological data) with Fisher's exact test with small numbers of expected frequencies (<5) or chi-square test for percentages and 95% exact confidence intervals, using the statistical program SAS.

## Results

### Participants

**Figure 2:**
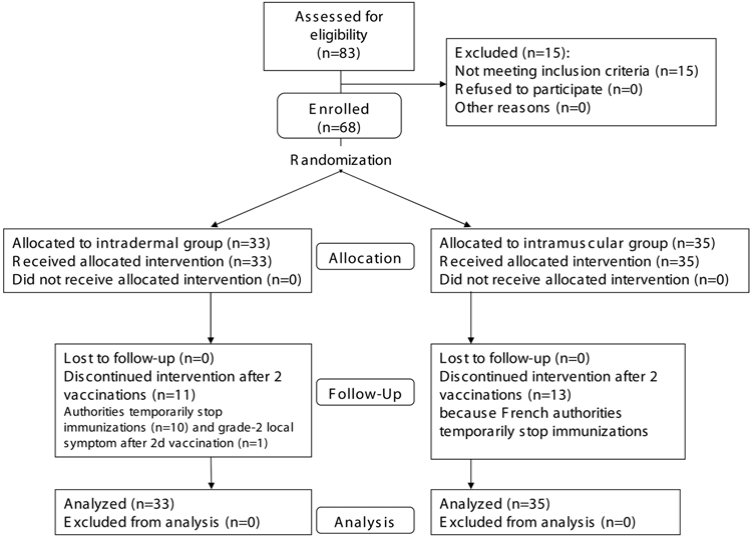
ANRS VAC16 Participant flow chart.

The first enrollment was on 17 August 2004, and the last study visit was on 28 June 2005. Sixty-eight subjects were enrolled and randomly assigned to one of the two treatment arms: 33 received intradermal injections and 35 intramuscular injections (Figure 2). Their demographic characteristics are given in Table 1. HLA types, able to induce CD8^+^ T-cell responses against 0–1 peptide, 2–5 peptides and 6–11 peptides, were present in 13 (19%), 21 (31%), and 34 (50%) of these subjects, respectively. All 68 subjects, 33 in the intradermal group and 35 in the intramuscular group, received the first two vaccinations and 44, 22 in each group, received the third. One volunteer experienced a grade 2 local symptom after the second intradermal vaccination and did not receive the third. Vaccinations were halted for 23 volunteers because a severe adverse event occurring in a LIPO-5 vaccine trial [31]. In this other study, one volunteer developed neurological symptoms 6 weeks after the second LIPO-5 immunization. The diagnosis of myelitis was retained. This trial conducted by the NIH in the United States has been suspended and surveillance of the volunteers is maintained. This event led the French authorities to stop all such immunizations temporarily. Although a meta-analysis of lipopeptide trials conducted during that period concluded that lipopeptide-vaccine safety was acceptable [29], the interval was too long to resume LIPO-4 vaccinations and to reach 70 planned inclusions. Subjects who received 2 vaccinations were followed for safety assessment and immune responses according to the protocol until the end of the study and thus, data for all 68 subjects were available for reactogenicity and immunogenicity analyses.

**Table 1:** Demographic characteristics of trial participants as a function of vaccination route.

Characteristic	Intradermal (N = 33)	Intramuscular (N = 35)	Total (N = 68)
Sex, no. (%)			
Male	28 (85)	23 (66)	51 (75)
Female	5 (15)	12 (34)	17 (25)
Age, years, no. (%)			
21–30	2 (6)	2 (6)	4 (6)
31–40	9 (27)	7 (20)	16 (24)
41–50	16 (48)	14 (40)	30 (44)
51–55	6 (18)	12 (34)	18 (26)
Median [range]	44 [28–56]	48 [27–56]	47 [27–56]
Vaccinations received, no. (%)			
Day 0	33 (100)	35 (100)	68 (100)
Week 4	33 (100)	35 (100)	68 (100)
Week 12	22 (67)	22 (63)	44 (65)

### Safety

No grade-3 or -4 adverse events were reported during the study (Table 2). Thirty-three (95% confidence interval [CI], 18–52) and 17% (7–34) of intradermally and intramuscularly injected participants, respectively, experienced a grade 2 adverse event possibly, probably or certainly vaccination-associated, excluding injection-site pain (P = 0.12). These percentages, without excluding injection-site pain, were 33 (18–52) and 31 (17–49) for intradermally and intramuscularly injected participants, respectively (P = 0.87). Injection-site pain was significantly less frequent after intradermal injections (27% (13–45) in intradermal group vs 80% (63–91) in intramuscular group; P<0.0001), but induration and pruritus were more common. All resolved without sequelae. In this small study, incidence of adverse events (grade-2 or higher, possibly, probably or certainly related, local pain excluded) was 5/24 (21% (7–42)) in subjects who received 2 injections and 12/44 (27% (15–43)) for subjects who received 3 injections; detailed analysis also revealed that local events were as frequent after the first, second, and third injections (6% (2–14), 15% (7–25) and 9% (3–22) of subjects after 1, 2 and 3 injections, respectively) and systemic reactions were more reported after the first injection (12% (5–22), 3% (0–10) and 2% (0–12) of subjects after 1, 2 and 3 injections, respectively). Only one intramuscularly injected volunteer developed a vaccine-related fever (38.5°C). The frequencies of reported systemic vaccine-related adverse events (myalgia, headache, nausea, asthenia and diarrhea) were comparable for the two groups.

**Table 2:** Frequencies, by maximum severity, of local symptoms and systemic reactions possibly, probably or certainly related to vaccination, as a function of immunization route.

	Intradermal (N = 33)	Intramuscular (N = 35)
All Reactions		
Any	33 (100)	34 (97)
Grade 2^a^	11 (33)	11 (31)
Grade 2 (pain excluded)^b^	11 (33)	6 (17)
Local Symptoms		
Any	33 (100)	32 (91)
Grade 2	8 (24)	7 (20)
Injection-site pain		
Any	9 (27)	28 (80)
Grade 2	0	6 (17)
Injection-site erythema		
Any	32 (97)	26 (74)
Grade 2	3 (9)	0
Injection-site induration		
Any	29 (88)	10 (29)
Grade 2	1 (3)	0
Injection-site pruritus		
Any	24 (73)	4 (11)
Grade 2	5 (15)	0
Injection-site edema		
Any	4 (12)	4 (11)
Grade 2	1 (3)	1 (3)
Other local symptoms		
Any	14 (42)	9 (26)
Grade 2^c^	2 (6)	0
Systemic Reactions		
Any	17 (52)	18 (51)
Grade 2	5 (15)	5 (14)
Myalgia		
Grade 1	3 (9)	4 (11)
Headache		
Any	3 (9)	4 (11)
Grade 2	0	1 (3)
Nausea		
Any	2 (6)	4 (11)
Grade 2	1 (3)	1 (3)
Asthenia		
Any	3 (9)	3 (9)
Grade 2	2 (6)	1 (3)
Diarrhea		
Any	1 (3)	2 (6)
Grade 2	1 (3)	0
Arthralgia		
Grade 1	2 (6)	0
Other systemic symptoms		
Any	6 (18)	10 (29)
Grade 2^d^	1 (3)	3 (9)

Tabulated values are numbers (percentages) of volunteers experiencing at least one reaction after any vaccination. Percentages do not add up to 100 because some volunteers had >1 symptom and/or reaction.

All reactions resolved without sequelae within a median of 56 hours (interquartile range 9–304 hours).

a 95% confidence interval in intradermal (ID) arm:18 to 52% and in intramuscular (IM) arm: 17 to 49%; ID vs. IM P = 0.87.

b 95% confidence interval in ID arm:18 to 52% and in IM arm: 7 to 34%; ID vs. IM P = 0.12.

c Other grade-2 local symptoms in the ID arm: local myalgia, injection-site vesicles.

d Other grade-2 systemic reactions in the IM arm: conjunctivitis, fatigue and rhinitis; in the intradermal arm: flu-like syndrome.

### Immunogenicity

#### Induction of HIV-Specific CD8^+^ T-Cell Responses

**Figure 3:**
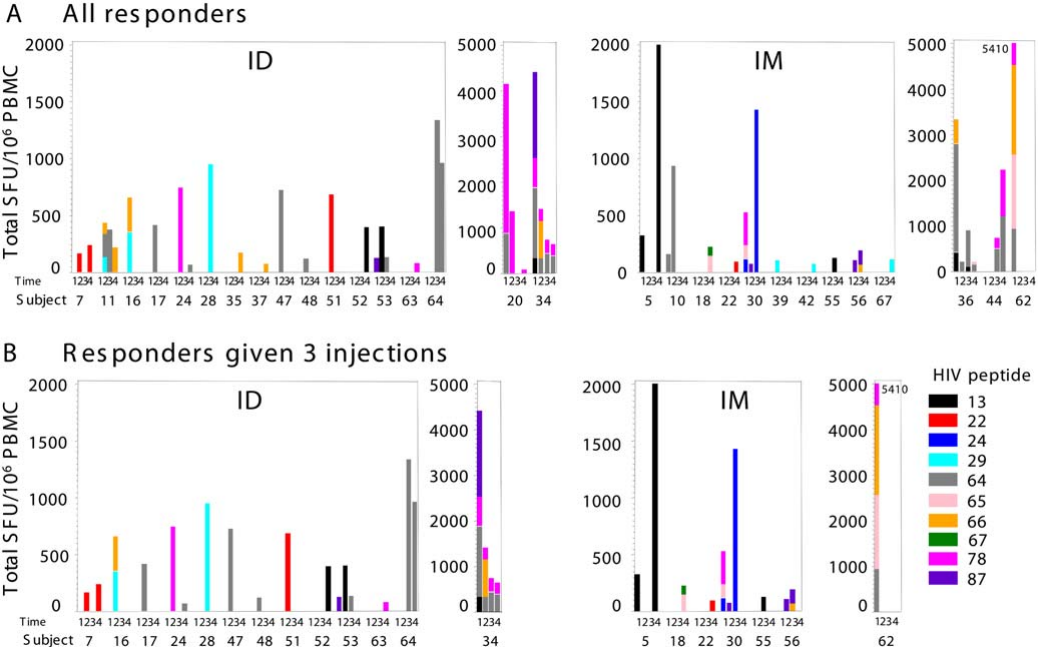
CD8^+^ T-cell responses (ELISpot IFNγ) to HIV peptides as a function of vaccination route, expressed as individual response. Panel A, all responders (N = 68), and Panel B, responders given 3 injections (N = 44°. For each responder, the magnitude of the response against individual HIV peptides, expressed as the total spot-forming units per million peripheral blood mononuclear cells (SFU/10^6^ PBMC), evaluated at times 1–4 corresponding to weeks 2, 6, 14 and 24.

**Figure 4:**
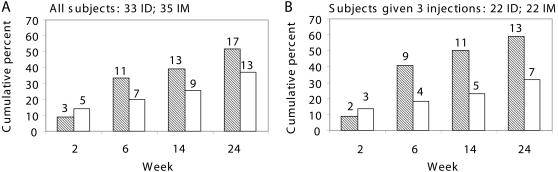
CD8^+^ T-cell responses (ELISpot IFNγ) to HIV peptides as a function of vaccination route, expressed as cumulative percentages. Panel A, all volunteers (N = 68), and Panel B, volunteers who received 3 injections (N = 44). The numbers of volunteers who had mounted responses to at least one HIV peptide at weeks 2, 6, 14, 24 are given above the columns: after intradermal (ID, crosshatched) or intramuscular (IM, empty) injections.

Few responses were seen before vaccination (5 and 2 subjects in intradermal and intramuscular groups respectively, against 1 peptide in 6 cases, and 2 peptides in 1 case) and in most cases, these responses were no more detected after vaccination excepted in one case. Specific cellular responses mounted by PBMC from 17/33 intradermally vaccinated subjects were positive: 11, 3 and 3 generated CD8^+^ T cells against one, two or more peptides, respectively (Figure 3). Thirteen of the 35 intramuscular vaccine recipients had HIV-specific IFNγ secretion by their CD8^+^ T cells generated against one, two or more peptides, for 8, 3 and 2 of them, respectively. Cumulative percentages (95% CI) of CD8^+^ T cells secreting IFNγ in response to at least one HIV peptide were at weeks 2, 6, 14 and 24, respectively: 9 (2–25), 33 (18–52), 39 (23–58) and 52 (34–69) for intradermally injected volunteers, and 14 (5–31), 20 (8–37), 26 (12–43) and 37 (21–55), for intramuscularly injected volunteers. None of the comparisons between routes were significant at the times tested (P = 0.71, 0.21, 0.23, and 0.23 at weeks 2, 6, 14, and 24, respectively). For subjects who received three intradermal or intramuscular injections, the respective cumulative numbers and percentages at weeks 2, 6, 14 and 24 are reported in Figure 4. Thus, intradermal immunization elicited CD8^+^ T-cell responses similar to responses after intramuscular injections at every time tested.

Ten CD8^+^ T-cell–stimulating HIV peptides, among the 12 sequences spanning the vaccine's four lipopeptides, were recognized by T cells from vaccinated volunteers: peptide 24 by one intramuscularly vaccinated subject; peptide 13 by six subjects (three per group); peptides 22, 78 and 87, by twelve subjects (seven and five subjects from the intradermal and intramuscular arms, respectively); peptides 29, 64, 65, 66 and 67, by twenty-three subjects (13 and 10 subjects immunized intradermally and intramuscularly, respectively).

Lipopeptides TT–Nef and TT–Pol1, which contain several epitopes unlike lipopeptides TT–Gag and TT–Pol2, were able to induce most IFNγ-secreting CD8^+^ T-cell responses. Unsurprisingly, HLA-B7 supertype (HLA-B7/-35/-51)-restricted epitopes were preferentially recognized, reflecting their high representation (5/12 epitopes) in LIPO-4.

### CD4^+^ T-Cell Proliferation in Response to Tetanus Toxin Helper Epitope

**Figure 5:**
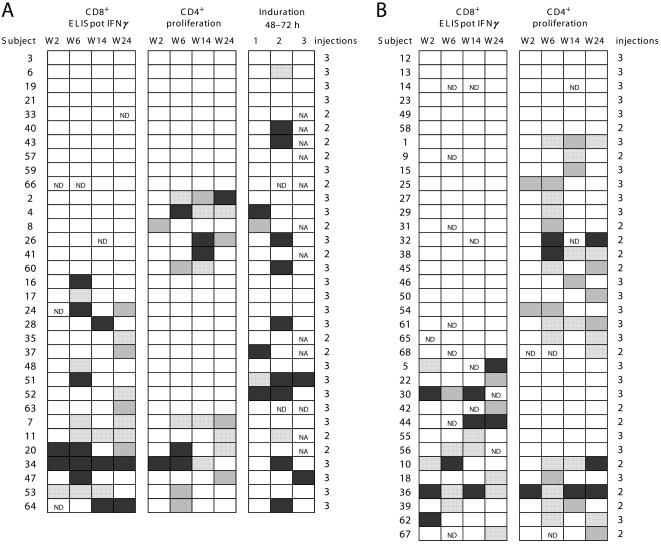
Summary of responses induced in vaccinated volunteers after 1, 2 or 3 intradermal (Panel A, N = 33) or intramuscular (Panel B, N = 35) injections. Responses were evaluated at weeks 2, 6, 14 and 24 as follows: CD8^+^ T-cell responses to HIV peptides, assessed with ELISpot IFNγ (total SFU/10^6^ PBMC defined as total SFU of positive responses minus the background: nonresponder empty; <100 crosshatched; 100–<500 solid squares; 500+ dotted); CD4^+^ T-cell proliferation against TT peptide (stimulation index: <3 empty; 3–<5 crosshatched; 5–<10 dotted; >10 solid) and induration present 48–72 hours postinjection (diameter (mm): none open; <5 crosshatched; 5–<10 dotted; >10 solid). Subjects in each panel are ordered as follows: CD8^+^ T-cell nonresponders (ELISpot IFNγ), CD4^+^ T-cell responders (proliferation) and, finally, CD4^+^ T-cell responders. Injections were given at weeks 0 and 4 to all 68 subjects and also at week 12 to 44 subjects ND, not done; NA, not applicable.

Proliferative responses of PBMC from 13/33 (39%; 95% CI, 23–58) intradermally immunized volunteers were mostly obtained after at least two LIPO-4 injections (two, seven and four subjects responded after one, two or three injections, respectively) (Figure 5). Stimulation indexes ranged from 3 to >10, with nine subjects having stimulation indexes >5 and six with indexes >10. Proliferative responses were mounted by CD4^+^ T-cells from 22/35 (63%; 95% CI, 45–79) volunteers injected intramuscularly (22/35 for intramuscular versus 13/33 for intradermal group, P = 0.053), with only three responses after a single injection. Thirteen subjects had stimulation indexes >5. Response intensities, measured by stimulation indexes (figure 5), did not differ between groups at any time point. Thus, intramuscular LIPO-4 injection preferentially elicited CD4^+^ T-cell responses to TT epitope.

## Discussion

Strategies to improve vaccine immunogenicity include increasing antigen content and/or the number of immunizations, adding adjuvant and/or alternative immunization routes. The efficacy of vaccine delivery to the dermal compartment, one of the body's most immunocompetent sites, is probably attributable to contact between deposited antigen and abundant professional antigen-presenting cells. Recent results describe lipopeptide penetration into dendritic cells and, once internalized, their processing via the cross-presentation pathway into peptides that are presented to CD8^+^ T lymphocytes [20].

Herein, the outcome of intradermal antigen delivery was evaluated in terms of safety, CD4^+^ and CD8^+^ T-cell immune responses, and injection-site reactions after each immunization, in comparison to classical intramuscular injection. As in previous clinical trials evaluating HIV lipopeptides [29], our LIPO-4 vaccine was well tolerated. No grade 3 or 4 reactions were observed. Systemic adverse reactions after vaccination occurred at similar frequencies in both groups. Local reactions (induration, pruritus) were more frequent in intradermally vaccinated subjects, but injection-site pain was more frequent after intramuscular immunization. Similar findings were obtained in other trials evaluating intradermal injection [24].

The optimal way to measure antigen-specific T cells remains a matter of debate, especially since recent observations suggest that different assays might measure different T-cell responses. Although the detection of ex vivo IFNγ-producing cells using the ELISpot assay might be useful for identifying immunogenic vaccines, it may be less informative for the identification of a protective immune response [32], [33]. Previous evaluations of vaccine responses were based on CTL chromium-release assays using in vitro-enriched T cells. That approach, by favoring the amplification of dividing T cells, enabled resting central memory T cells to differentiate into effector cells. In accordance with that rationale, we, like others [14], [32], [34], developed a cultured ELISpot assay using well-defined CD8^+^ T-cell–stimulating epitopes. To examine the kinetics of the T-cell responses, we assessed immune responses after every LIPO-4 immunization. The study of cumulative response, which allows evaluation after each immunization, is a sensitive approach even in the case of a transient response which could be enhanced after booster injections [14].

Our findings indicate that HIV-specific T-cell responses were induced in vaccinated subjects. CD8^+^ T-cell IFNγ secretion in response to at least one HIV peptide at weeks 2, 6, 14 and 24, expressed as cumulative percentages, was, respectively, 9, 33, 39 and 52, for intradermally injected volunteers and 14, 20, 26 and 37, for intramuscularly injected volunteers. In contrast, the respective percentages of TT peptide-specific CD4^+^ T-cell proliferation were 6, 27, 33 and 39 (intradermal arm) and 9, 46, 54 and 63 (intramuscular arm). To our knowledge, these are the first data suggesting that the immunization route might affect the CD4^+^ and CD8^+^ T-cell phenotypes.

Primary immunization encounters an immune system naive to the vaccine antigen. When the antigen is readministered in booster shots, antigen-specific, cellular delayed-type hypersensitivity reactions are triggered. Therefore, we scored skin responses during the first 72 hours after antigen administration. The nature of skin responses is not completely understood: no obvious relationship was observed with lymphocyte proliferation, CD8^+^ induction, delayed hypersensitivity responses and/or intensity or durability of the skin reactions, as shown figure 5. In terms of response breadth and in contrast to our results obtained in the SIV–macaque model using a LIPO-5 formulation injected intradermally, we could not establish any difference between intradermal and intramuscular routes concerning immune response induction. This finding probably indicates that LIPO-4, because it contains only 12 potential epitopes, is not suitable to measuring such parameters. Intradermal immunization efficacy was reported in human clinical studies on rabies, hepatitis B and, more recently, influenza [21]–[23]. In most of those trials, intradermal injections required less antigen than intramuscular immunization to elicit an equivalent antibody response. In the two studies comparing the two routes for influenza vaccination, intradermal injection with 40 or 20% of the standard intramuscular dose induced humoral immunogenicity comparable to that of the full intramuscular dose [24], [25]. None of those studies evaluated T-cell–response induction after intradermal injection. Because of the conflicting data and awaiting new studies, these vaccines are still intramuscularly administered.

In conclusion, our results demonstrate that intradermally injected HIV lipopeptides induce complementary CD4^+^ and CD8^+^ T-cell responses. Moreover, intradermal injection of 20% of the intramuscular dose of LIPO-4 vaccine was at least as immunogenic as the full intramuscular dose, and would be a valid dose-sparing strategy, although the study was not powered to demonstrate non-inferiority. However, reduced intramuscular doses have not yet been evaluated and a study comparing 3 doses of HIV lipopeptides versus placebo in non-infected volunteers is currently ongoing (ClinicalTrial.gov identifier: NCT00121758). Our observations also suggest that combining intradermal and intramuscular vaccination routes might be a new approach to improve induction of cellular immunity and warrants further evaluation.

## Supporting Information

Protocol S1 Trial protocol.
(0.55 MB DOC)

Checklist S1 CONSORT checklist.
(0.05 MB DOC)
