# Supplementary material for: Cellular Immune Responses Induced with Dose-Sparing Intradermal Administration of HIV Vaccine to HIV-Uninfected Volunteers in the ANRS VAC16 Trial
Source: PLoS One. 2007 Aug 22;2(8):e725. doi: 10.1371/journal.pone.0000725 (PMC1942115; doi:10.1371/journal.pone.0000725)
Supplement: Protocol S1 — Trial protocol. (0.55 MB DOC) [file pone.0000725.s001.doc]

Essai vaccinal de phase I-B comparant la tolérance et l'immunogénicité de deux voies d’administration (intramusculaire et intradermique) de lipopeptides (LPVIH1)

spécifiques du virus VIH-1 chez des volontaires non infectés par le VIH

PROTOCOLE D’ETUDE CLINIQUE DE PHASE IB SANS BENEFICE INDIVIDUEL DIRECT

ANRS VAC 16

**Promoteur** Agence Nationale de Recherche sur le Sida (ANRS)

101, rue de Tolbiac, 75013 Paris, France

contact : Dr. Véronique Rieux

Tel : 01.53.94.60.15 Fax : 01.53.94.60.03

E-mail : véronique.rieux@anrs.fr

**Coordination scientifique** Pr. Dominique Salmon

Hôpital Cochin, Centre Cochin-Pasteur d’Essais Vaccinaux

27, rue du Faubourg Saint-Jacques, 75679 Paris Cedex 14, France.

Tél : 01.58.41.21.29 Fax : 01.43.26.88.92

E-mail : dominique.salmon@cch.ap-hop-paris.fr

**Investigateur coordonnateur** Dr. Odile Launay

Hôpital Cochin, Centre Cochin-Pasteur d’Essais Vaccinaux

27, rue du Faubourg Saint-Jacques, 75679 Paris Cedex 14, France.

Tél : 01 44.07.15.41 Fax : 01.40.46.93.08

E-mail : odile.launay@cch.ap-hop-paris.fr

**Co-investigateur** Dr. Benjamin Silbermann

Hôpital Cochin, Centre Cochin-Pasteur d’Essais Vaccinaux

27, rue du Faubourg Saint-Jacques, 75679 Paris Cedex 14, France.

Tél : 01 58.41.19.11 Fax : 01.40.46.93.08

E-mail : benjamin.silbermann@cch.ap-hop-paris.fr

**Chef de projet du Centre** Corinne Desaint

**coordinateur** Hôpital Cochin, Centre Cochin-Pasteur, d’Essais Vaccinaux

27, rue du Faubourg Saint-Jacques, 75679 Paris Cedex 14, France.

Tél : 01.56.24.14.75 Fax : 01.40.46.93.08

E-mail : corinne.desaint@cch.ap-hop-paris.fr

**Immunologistes** Hanne Gahery

Département d’Immunologie de l’Institut Cochin

22, rue Méchain, 75014 Paris, France.

Tél  01.43.21.04.80 / 01.40.51.65.33 Fax : 01.40.48.83.52 / 01.40.51.65.35

[gahery@cochin.inserm.fr](mailto:guillet@cochin.inserm.fr)

**Méthodologiste et** Christine Durier

**statisticienne** SC 10, INSERM, Hôpital Paul Brousse,

16, avenue Paul Vaillant Couturier, 94807 Villejuif, France.

Tél : 01.45.59.51.56 Fax : 01.45.59.51.80

E-mail: c.durier@vjf.inserm.fr

**Pharmacienne coordinatrice** Corinne Guérin

Pharmacie/Secteur des essais cliniques, Hôpital Cochin

27, rue du faubourg Saint-Jacques, 75 679 PARIS cedex 14

Tél : 01.58.41.23.09 Fax : 01.58.41.23.12

E-mail: corinne.guerin@cch.ap-hop-paris.f

**Version n°** 3.1

**Date** 02/03/04

# Page de signatures

ESSAI VAC 16

Accord sur la version n° 3.1 du / 03 / 2004 approuvée par le CCPPRB de la Pitié Salpétrière

**Promoteur :**

Agence Nationale de Recherche sur le SIDA

101, rue de Tolbiac,

75013 PARIS

représentée par le Pr. Michel KAZATCHKINE

Pr. Michel KAZATCHKINE

Date :

Signature :

**Investigateur Coordonnateur**

Dr. Odile Launay

Centre Cochin-Pasteur d’Essais Vaccinaux

Hôpital Cochin

27, rue du Faubourg Saint-Jacques

75014 Paris

Dr. Odile LAUNAY

Date :

Signature :

**Pharmacologue**

Dr. Bernard Rouveix

Service de Pharmacologie

Hôpital Cochin

27, rue du Faubourg Saint-Jacques

75014 Paris

Dr. Bernard ROUVEIX

Date :

Signature

# Résumé

Promoteur ANRS

**Titre de l’essai** Essai vaccinal de phase IB comparant la tolérance et l'immunogénicité de deux voies d’administration (intramusculaire et intradermique) de lipopeptides (LPVIH1) spécifiques du virus VIH-1 chez des volontaires non infectés par le VIH.

**Code de l’essai** ANRS VAC 16

**Phase clinique** IB

**Investigateur** Dr. Odile Launay

**coordonnateur** Hôpital Cochin, Centre Cochin-Pasteur d’Essais Vaccinaux,

27 rue du faubourg Saint Jacques, 75679 Paris Cedex 14

**Co-investigateur** Dr. Benjamin Silbermann

Hôpital Cochin, Centre Cochin-Pasteur d’Essais Vaccinaux,

27 rue du faubourg Saint Jacques, 75679 Paris Cedex 14

**Centres de l’essai** - Centre Cochin-Pasteur d’Essais Vaccinaux, Hôpital Cochin, Paris (centre coordonnateur)

- Service des Maladies Infectieuses et Tropicales, Hôpital Tenon, Paris

- Service d’Immunologie Clinique, HEGP, Paris

- Service d’Hématologie, Hôpital Ste Marguerite, Marseille

- Service des Maladies Infectieuses et Tropicales, Hôpital Purpan, Toulouse

- Services des Maladies Infectieuses et Tropicales, Hôpital de Nantes, Nantes

**Produit testé** LPVIH1 est un mélange de 4 lipopeptides constitués de peptides de VIH1-LAI : Gag (aa 77-85), Pol (aa 342-354 et 476-484) et Nef (aa 68-82) associés chacun de façon colinéaire à un épitope T helper de la toxine tétanique (TT) 830-843 branché en N-terminal à un acide gras monopalmitique.

**Objectif principal** Evaluer la tolérance clinique (locale et générale) et biologique de LPVIH1 administré par voie intradermique et la comparer à celle de l’administration par voie intramusculaire, chez des volontaires non infectés par le VIH.

**Objectif secondaire** Comparer l'immunogénicité cellulaire de LPVIH1 administré selon ces deux voies

Sous-étude physiopathologique**:** Etudier la réponse immunitaire locale pour 6 des sujets ayant reçu LPVIH1 par voie intradermique.

**Méthodologie** Essai multicentrique, sans bénéfice individuel direct, randomisé, stratifié, ouvert

Schéma de l'essai

| Groupe | Voie d’administration | Dose de LPVIH1  (dose totale) | Sujets  (Total 70) | Schéma d'immunisation en semaines | | | |
| --- | --- | --- | --- | --- | --- | --- | --- |
|  |  |  |  | 0 | 4 | 12 | 24§ |
| A | intramusculaire | 500µg  (2000µg) | 35 | LPVIH1 | LPVIH1 | LPVIH1 |  |
| B | intradermique | 100µg  (400µg) | 35 (dont 6 §) | LPVIH1 | LPVIH1 | LPVIH1 | LPVIH1§ |

§ : sujets participant à la sous-étude physiopathologique et ayant une biopsie cutanée à S24+48H

**Taille prévue de** 70 sujets

**l’échantillon** Les sujets sont répartis en 3 strates suivant leur typage HLA (HLA permettant de tester 0 à 1 peptide VIH-1 ; 2 à 5 peptides, 6 à 11 peptides).

**Période d’essai prévue** mai 2004 à juin 2005

**Durée du suivi** 32 semaines pour tous les volontaires (soit 6 semaines pour la pré-inclusion, 26 semaines de suivi à partir de l’inclusion)

**Critères d’inclusion** *Critères cliniques*

*sujets âgés de 21 à 55 ans.

*volontaires du réseau ANRS.

*femmes qui ne devront pas être enceintes ou allaitantes et devront accepter de poursuivre ou d'entreprendre, pendant toute la durée de l'essai, un moyen de contraception considéré comme efficace par l’investigateur, ou devront présenter une stérilité documentée ou être ménopausées.

*sujets présentant un examen clinique normal

*sujets affiliés à un régime de la Sécurité Sociale ou bénéficiaires d’une assurance maladie.

*sujets ayant signé le consentement éclairé.

*sujets pouvant recevoir le produit à l’essai indifféremment par l’une des 2 voies d’administration.

*Critères biologiques*

*absence d’anomalies cliniquement significatives aux tests biologiques standards pouvant gêner l’interprétation des résultats de tolérance.

*sérologies VIH-1 et -2, virus hépatite B, C, HTLV-1 et syphilis négatives.

*Hb> 12,5g/dl pour les femmes et > 13,5g/dl pour les hommes

*férritinémie normale

**Critères de**

**non-inclusion** *sujets avec un risque identifiable de contracter une infection VIH d'après les entretiens de recrutement initial

*sujets ayant participé à un autre essai clinique relatif à l'infection à VIH ou concernant un immuno-modulateur, participant à un autre essai sans bénéfice individuel direct ou étant dans la phase d'exclusion d'un essai clinique relatif à un autre produit.

*sujets ayant eu une vaccination dans le mois précédant la première injection ou nécessitant une vaccination pendant la durée de l’essai.

*sujets ayant fait un don de sang dans les 2 mois précédant la visite de pré-inclusion.

*sujets ayant des antécédents d'allergie alimentaire de l'adulte documentée, de syndrome de Lyell, de Stevens-Johnson, d'asthme grave décompensé.

*sujets ayant un eczéma généralisé actif.

*sujets ayant un urticaire chronique évolutif en cours.

*sujets ayant eu une uvéite ou ayant un risque de développer une uvéite (des maladies auto-immunes, syphilis, maladie de Lyme, mycobactérioses évolutives, sarcoïdose).

*sujets suivant un traitement immuno-modulateur au long cours (interférons ou autres cytokines ou immunostimulants) ou l'ayant suivi dans les six mois précédant l'essai (chimiothérapie, corticothérapie, radiothérapie)

*sujets ayant un traitement médicamenteux en cours susceptible de modifier la réponse immunitaire (AINS) et/ou
 responsable de perturbations biologiques pouvant gêner l'interprétation des résultats de tolérance.

*sujets ayant eu dans leur passé une affection médicale grave évolutive ou des antécédents de maladies auto-immunes (syndrome de Bechet, spondylarthrite ankylosante, polyarthrite rhumatoïde, maladie de Bowen, psoriasis, syndrome de Reiter, connectivite, maladie neurologique démyélinisante, colite inflammatoire) ou ayant laissé des séquelles cliniques et/ou biologiques persistantes.

*sujets ayant reçu une transfusion de sang total ou des dérivés sanguins plasmatiques dans les 6 mois précédant l'essai

*sujets ayant été traités par des hormones hypophysaires d'extraction.

*sujets présentant des conditions psychosociologiques et géographiques incompatibles avec l'essai (population
 instable, mobilité géographique)

**Produit à l’essai** LPVIH1 (mélange des 4 lipopeptides)

**Forme s**uspension injectable

**Dose** 600 µg de chaque lipopeptide (600 µg x 4) soit 2400 µg dans 0,6 ml de solution glucosée à 5% .

**Lot n°** BT358038A

**Voies** - Voie intramusculaire dans le muscle deltoïde

**d’administration** - Voie intradermique dans la face externe du bras (pour la sous-étude physiopathologique, l’injection se fera à la face interne du bras)

**Dose administrée** 0,5 ml soit 500µg de chaque peptide VIH-1 (au total, 2000µg solubilisés dans une solution glucosée à 5% pour la voie intramusculaire ).

0,1 ml soit 100µg de chaque peptide VIH-1 (au total, 400µg solubilisés dans une solution glucosée à 5% pour la voie intradermique)

**Critères d’évaluation**

***critère principal*** • Evènements cliniques (locaux ou généraux) et/ou biologiques de degré ≥ 2 (à l’exception des douleurs au point d’injection)

[Les événements indésirables de grade 3 ou 4 liés au produit seront recensés en temps réel et la survenue d’un événement de grade 4 et/ou la survenue chez au moins 3 volontaires d’événements locaux et/ou généraux de grade 3, dans le bras intradermique, entraînera l’arrêt de l’essai.]

***critères secondaires*** • Evènements cliniques (locaux ou généraux) et/ou biologiques tous degré confondus

• Réponses CD4+ prolifératives sur sang frais sans déplétion.

• Capacité des CD8+ sanguins périphériques à sécréter de l’INF-γ en présence des peptides et des épitopes présents dans le mélange LPVIH1 (tests ELISPOT INF-γ)

• Capacité des CD8+ sanguins tissulaires à synthétiser de l’INF-γ (sous-étude physiopathologique)

Méthodes statistiques

• L’évaluation de la tolérance sera effectuée sur toute la durée de l’étude, principalement au décours des vaccinations. L’immunogénicité sera évaluée en prenant en compte les réponses à S2, S6, S14 et la durabilité de la réponse induite sera évaluée à S24.

• Une analyse descriptive classique sera effectuée avec calculs de fréquences, de médianes, moyennes et leurs intervalles de confiance.

• Les méthodes statistiques utilisées compareront les groupes à temps fixes (tests du Chi-2 et Fisher exact pour les variables dichotomiques, tests paramétriques et non-paramétriques pour les variables quantitatives). On utilisera des tests bilatéraux au seuil 5%.

•Une taille d'échantillon de 35 sujets par groupe est justifiée par la limite de confiance du taux de survenue d’évènements indésirables de degré ≥3 et la comparaison des taux de survenue d’événements de grade ≥2 (douleurs au point d’injection exclues). L’immunogénicité (réponse des cellules T CD8+) sera évaluée uniquement chez les volontaires dont le typage HLA laisse prévoir une réponse à au moins deux peptides (80% des volontaires environ).

Schéma synoptique VAC 16

| **Visites (n°)** | **V1** | **V2** | **V3** | **V4** | **V5** | **V6** | **V7** | **V8** | **V9§** | **V10§** |
| --- | --- | --- | --- | --- | --- | --- | --- | --- | --- | --- |
| **Date (S: semaine; J: jour)** | **S-6** | **S0** | **S2** | **S4** | **S6** | **S12** | **S14** | **S24** | **S24+J2** | **S24+J12** |
| Intervalle (jours) |  | ±7j | ±3j | ±7j | ±3j | ±7j | ±3j | ±14j | ±0j | ±3j |
| **Examen clinique** | **✓** | **✓** | **✓** | **✓** | **✓** | **✓** | **✓** | **✓** | **✓** | **✓** |
| NFS/plaquettes, CD4/CD8, lymphocytes B totaux | ✓ | ✓ | ✓ | ✓ | ✓ | ✓ | ✓ | ✓ |  |  |
| TP/TCA/VS | ✓ |  |  |  |  |  |  |  |  |  |
| Urée/Créatininémie | ✓ |  | ✓ |  | ✓ |  | ✓ | ✓ |  |  |
| Ionogramme sanguin, calcémie, phosphorémie, glycémie, protéines totales | ✓ |  |  |  |  |  |  |  |  |  |
| ASAT/ALAT/phosphatases alcalines, CPK, bilirubine totale, | ✓ |  |  |  |  |  |  | ✓ |  |  |
| GammaGT |  |  |  |  |  |  |  |  |  |  |
| ferritine | ✓ |  |  |  |  | ✓ |  | ✓ |  |  |
| Dosage pondéral Ig (Ig G, A, M) | ✓ |  |  |  |  |  | ✓ |  |  |  |
| Dosage du complément |  | ✓ |  | ✓ |  | ✓ |  | ✓§ |  |  |
| Auto-anticorps (ADN, noyaux, mitochondries, muscle lisse), | ✓ |  |  |  |  |  |  | ✓ |  |  |
| Typage HLA (classes I (A et B) et II (DR, DQ)) | ✓ |  |  |  |  |  |  |  |  |  |
| Sérologie tétanique | ✓ |  |  |  |  |  |  | ✓ |  |  |
| Sérologie VIH-1 (ELISA et WESTERN-BLOT VIH-1 et -2) | ✓ |  |  |  |  |  |  | ✓ |  |  |
| Sérologie VIH (ELISA et WB VIH-1 ssi ELISA positif) |  |  | ✓ |  | ✓ |  | ✓ |  |  |  |
| Ag P24* |  |  |  |  |  |  |  |  |  |  |
| Sérologies VHC, TPHA/VDRL**, Ag Hbs**, Ac Hbs**,Ac Hbc**,HTLV-1 | ✓ |  |  |  |  |  |  |  |  |  |
| Béta-HCG plasmatiques | ✓ |  |  |  |  |  |  | ✓ |  |  |
| Immunité cellulaire (poche de 100 ml de sang) | ✓ | ✓ | ✓ |  | ✓ |  | ✓ | ✓ ou ✓§ | ✓§ |  |
| Sérothèque | ✓ | ✓ | ✓ | ✓ | ✓ | ✓ | ✓ | ✓ |  |  |
| Plasmathèque | ✓ |  | ✓ |  | ✓ |  | ✓ | ✓ |  |  |
| Test de grossesse urinaire |  | ✓ |  | ✓ |  | ✓ |  | ✓§ |  |  |
| Bandelette urinaire | ✓ | ✓ | ✓ | ✓ | ✓ | ✓ | ✓ | ✓ |  |  |
| **Vaccination** |  | **1** |  | **2** |  | **3** |  | **4§** |  |  |
| Surveillance immédiate (30 min.pour IM et ID) |  | ✓ |  | ✓ |  | ✓ |  |  |  |  |
| Tolérance Locale & Systémique |  | ✓ | ✓ | ✓ | ✓ | ✓ | ✓ | ✓ | ✓§ | ✓ |
| Biopsie |  |  |  |  |  |  |  |  | ✓§ |  |
| Remise carnet d’auto-surveillance |  | ✓ (1) |  | ✓(2) |  | ✓(3) |  | ✓(4)§ |  |  |
| Report carnet d’auto-surveillance sur CRF |  |  |  | ✓(1) |  | ✓(2) |  | ✓(3) |  | ✓(4)§ |
| Volume de sang prélevé Femme (ml) | 229 | 131 | 146 | 31 | 146 | 36 | 156 | 161/116§ | 50§ |  |
| Volume de sang prélevé cumulé Femme (ml) | 229 | 360 | 506 | 537 | 683 | 719 | 875 | 1036/991§ | 1036§ |  |
| Volume de sang prélevé Homme (ml) | 224 | 136 | 146 | 31 | 146 | 36 | 156 | 156/111§ | 50§ |  |
| Volume de sang prélevé cumulé Homme (ml) | 224 | 355 | 501 | 532 | 678 | 714 | 870 | 1026/981§ | 1026§ |  |

*chez les volontaires présentant une bande positive en protéine d’enveloppe au WB VIH-1 et/ou VIH-2 au prélèvement précédent, ** si pas de résultat inférieur à 6 mois.

§ : pour les sujets participant à la sous-étude physiopathologique (prélèvement immunologique S24=50ml et S24+48H=50ml)

Abréviations et symboles utilisés

**ADN** : Acide désoxyribonucléique

**ALAT** : Alanine aminotransférase

**ANRS** : Agence Nationale de Recherches sur le SIDA

**ASAT** : Aspartate aminotransférase

β**-HCG** : β Human Chorionotrophic Growth hormon

**Ca** : Calcium

**CCPPRB** : Comité Consultatif de Protection des Personnes dans la Recherche Biomédicale

**CD4, CD8** : Lymphocytes T CD4 ou T CD8 (cluster differenciation)

**Cl** : Chlore

**CMH** : Complexe majeur d'histocompatibilité

**CPCC** : Chef de Projet du Centre Coordinateur

**CPK** : Créatinine Phosphokinase

**CRF** : Case Report Form ou cahier d'observation

**CRO** : Contract Research Organization

**CTL** : Cytotoxic T lymphocytes

**DCI** : Dénomination Commune Internationale

**EBV** : Epstein-Barr virus

**EIG** : Evénement indésirable grave

**ELISA** : Enzyme linked immunosorbent assay

**Gamma GT** : Gamma glutamyl transferase

**gp120, 160, 41-MN** : glycoprotéine d'enveloppe du VIH1, isolat MN

**HLA** : Human leukocyte antigens

**HTLV-1 :** Human T cell Leukemia/lymphoma Virus de type 1

**ICH** : International Conference on Harmonisation

**ID** : Intradermique

**IgG, A, M** : Immunoglobulines de types G, A, M

**IM** : Intramusculaire

**J** : jour

**K** : Potassium

**LPVIH1** : mélange de 4 lipopeptides dont les séquences sont issues de VIH1

**Na** : Sodium

**NaCl** : Chlorure de Sodium

**PBMC:** Peripheral blood mononuclear cells

**Ph** : Phosphore

**PCR** : Polymerase Chain Reaction

**RT** : Reverse Transcriptase

**S** : Semaine

**SHIV** : Simian Hybrid immunodeficiency virus

**SIDA** : Syndrome d'immunodéficience acquise

**SIV** : Simian immunodeficiency virus

**VHC** : virus de l’hépatite C

**VIH** : (HIV) virus de l'Immunodéficience humaine (SIDA)

**VIH1/VIH2** : (HIV1/HIV2) virus de type 1/2 du SIDA

**VS** : Vitesse de sédimentation

TABLE DES MATIERES

**Page de signatures** [**2**](#__RefHeading___Toc66168040)

Résumé [3](#__RefHeading___Toc66168041)

1. Justification de l’étude [9](#__RefHeading___Toc66168042)

1.1.Epidémiologie [10](#__RefHeading___Toc66168043)

1.2. Positionnement de l’étude [10](#__RefHeading___Toc66168044)

1.2.1. Etat de la recherche vaccinale contre le VIH [10](#__RefHeading___Toc66168045)

1.2.2. Les lipopeptides [11](#__RefHeading___Toc66168046)

1.2.2.1. Structures des lipopeptides du VIH [12](#__RefHeading___Toc66168047)

1.2.2.2. Tolérance des lipopeptides chez l’animal [12](#__RefHeading___Toc66168048)

1.2.2.3. Expérience clinique avec les lipopeptides du VIH [13](#__RefHeading___Toc66168049)

1.2.3 Voies d’administration des lipopeptides [16](#__RefHeading___Toc66168050)

1.2.3.1.Avantages et inconvénients des différentes voies d’injection (18) [16](#__RefHeading___Toc66168051)

1.2.3.2.Voies d’injection utilisées pour l’administration des lipopeptides [17](#__RefHeading___Toc66168052)

1.2.4. Choix du type de lipopeptides et des doses de lipopeptides LPVIH1 pour l’essai. [18](#__RefHeading___Toc66168053)

2. objectifs de l’essai [19](#__RefHeading___Toc66168054)

2.1. Objectif principal [19](#__RefHeading___Toc66168055)

2.2. Objectifs secondaires [19](#__RefHeading___Toc66168056)

3. Schema de l’essai et methodologie [19](#__RefHeading___Toc66168057)

3.1. Schéma de l’essai [19](#__RefHeading___Toc66168058)

3.1.1. Schéma de l’essai [19](#__RefHeading___Toc66168059)

3.1.2. Procédures d’attribution des bras d’injection [20](#__RefHeading___Toc66168060)

3.2. Population de l’essai [21](#__RefHeading___Toc66168061)

3.2.1. Critères d’inclusion [21](#__RefHeading___Toc66168062)

3.2.2. Critères de non-inclusion [22](#__RefHeading___Toc66168063)

3.2.3. Attribution des numéros d’identification des sujets [23](#__RefHeading___Toc66168064)

3.2.4. Test de grossesse [23](#__RefHeading___Toc66168065)

3.2.5. Thérapies antérieures et concomitantes [24](#__RefHeading___Toc66168066)

3.3. Bénéfices pour les sujets/ risques potentiels [24](#__RefHeading___Toc66168067)

3.3.1. Bénéfices pour les volontaires [24](#__RefHeading___Toc66168068)

3.3.2. Risques potentiels [24](#__RefHeading___Toc66168069)

3.4. Arrêt prématuré de l’essai [26](#__RefHeading___Toc66168070)

3.5. Calendrier de l’essai [26](#__RefHeading___Toc66168071)

3.6. Schéma des vaccinations et plan des prélèvements [27](#__RefHeading___Toc66168072)

4.1. Caractéristiques du mélange de lipopeptides LPVIH1 [28](#__RefHeading___Toc66168073)

4.1.1. Description des lipopeptides (LPVIH1 ) [28](#__RefHeading___Toc66168074)

4.1.2. Composition des lipopeptides [28](#__RefHeading___Toc66168075)

4.2. Etiquetage et conditionnement [29](#__RefHeading___Toc66168076)

4.3. Conditions d’expédition et de stockage [29](#__RefHeading___Toc66168077)

4.4. Préparation et précautions d'utilisation des lipopeptides [29](#__RefHeading___Toc66168078)

4.5. Comptabilité des produits [30](#__RefHeading___Toc66168079)

4.6. Retour des produits inutilisés et/ ou entamés [30](#__RefHeading___Toc66168080)

5. organisation [31](#__RefHeading___Toc66168081)

5.1. Centres et personnels impliqués dans l’essai [31](#__RefHeading___Toc66168082)

5.2. Déroulement des visites [31](#__RefHeading___Toc66168083)

5.2.1. Visite de pré-inclusion [31](#__RefHeading___Toc66168084)

5.2.2. Randomisation [32](#__RefHeading___Toc66168085)

5.2.3. Participation à la sous-étude [33](#__RefHeading___Toc66168086)

5.2.3.1. Critères pour l’inclusion dans la sous-étude [33](#__RefHeading___Toc66168087)

5.2.3.2. Participation à la sous-étude [33](#__RefHeading___Toc66168088)

5.2.4. Visites d’inclusion et de vaccinations [34](#__RefHeading___Toc66168089)

5.2.5. Visites de suivi [35](#__RefHeading___Toc66168090)

5.2.6. Visites concernant la sous-étude [36](#__RefHeading___Toc66168091)

5.2.6.1. visite de vaccination [36](#__RefHeading___Toc66168092)

5.2.6.2. visite de la biopsie [36](#__RefHeading___Toc66168093)

5.2.6.3. visite de suivi [37](#__RefHeading___Toc66168094)

5.3. Procédures pour l’obtention et le traitement des échantillons [37](#__RefHeading___Toc66168095)

5.3.1. Prélèvement des échantillons de sang [37](#__RefHeading___Toc66168096)

5.3.2. Acheminement et préparation des échantillons en vue de l’analyse immunologique cellulaire périphérique et locale. [38](#__RefHeading___Toc66168097)

5.3.2.1. Acheminement [38](#__RefHeading___Toc66168098)

5.3.2.2. Préparation des cellules mononucléées au laboratoire d’Immunologie [38](#__RefHeading___Toc66168099)

5.3.3. Préparation de la sérothèque et de la plasmathèque [39](#__RefHeading___Toc66168100)

5.3.4. Préparation des échantillons de la biopsie en vue de l’analyse immunologique. [40](#__RefHeading___Toc66168101)

5.4. Cahiers d’observations et recueil des données [40](#__RefHeading___Toc66168102)

5.5. Carnets d’auto-surveillance [40](#__RefHeading___Toc66168103)

5.6. Conditions de sortie d’essai [41](#__RefHeading___Toc66168104)

5.6.1. Arrêt du traitement [41](#__RefHeading___Toc66168105)

5.6.2. Abandons [42](#__RefHeading___Toc66168106)

5.7. Procédures en cas de perdu de vue [42](#__RefHeading___Toc66168107)

5.8. Classification de fin d’essai [42](#__RefHeading___Toc66168108)

5.9. Monitoring, audit et archivage [43](#__RefHeading___Toc66168109)

5.9.1. Organisation générale [43](#__RefHeading___Toc66168110)

5.9.2. Visites de monitoring [43](#__RefHeading___Toc66168111)

5.9.3. Consignes et cahiers d’observations [44](#__RefHeading___Toc66168112)

5.9.4. Gestion des données [44](#__RefHeading___Toc66168113)

5.9.5. Audit, Inspection [45](#__RefHeading___Toc66168114)

5.9.6. Archivage [45](#__RefHeading___Toc66168115)

6. evenements indesirables [47](#__RefHeading___Toc66168116)

6.1. Définition et déclaration d’un événement indésirable grave [47](#__RefHeading___Toc66168117)

6.1.1. Définition [47](#__RefHeading___Toc66168118)

6.1.2. Déclaration des événements indésirables graves [48](#__RefHeading___Toc66168119)

6. 2. Conduite à tenir en cas d’événement indésirable [49](#__RefHeading___Toc66168120)

6. 3. Conduite à tenir en cas de survenue de grossesse [50](#__RefHeading___Toc66168121)

7. CRITERES D’EVALUATION [51](#__RefHeading___Toc66168122)

7.1. Critère principal d'évaluation [51](#__RefHeading___Toc66168123)

7.1.1. Définition [51](#__RefHeading___Toc66168124)

7.1.2. Paramètres de tolérance à mesurer [51](#__RefHeading___Toc66168125)

7.1.2.1 Tolérance clinique locale [51](#__RefHeading___Toc66168126)

7.1.2.2. Tolérance clinique générale [51](#__RefHeading___Toc66168127)

7.1.2.3. Surveillance clinique immédiate [52](#__RefHeading___Toc66168128)

7.1.2.4. Tolérance biologique [52](#__RefHeading___Toc66168129)

7.2.Critères secondaires d’évaluation [52](#__RefHeading___Toc66168130)

7.2.1. Définition [52](#__RefHeading___Toc66168131)

7.2.2. Méthode de mesure des critères secondaires [52](#__RefHeading___Toc66168132)

8. methodes statistiques et analyse des donnees [54](#__RefHeading___Toc66168133)

8.1. Détermination de la taille de l’échantillon [54](#__RefHeading___Toc66168134)

8.2. Randomisation et stratification [55](#__RefHeading___Toc66168135)

8.3.1. Populations incluses dans les analyses [56](#__RefHeading___Toc66168136)

8.3.2. Analyse du critère principal : tolérance [56](#__RefHeading___Toc66168137)

8.3.3. Analyse des critères secondaires : immunogénicité [56](#__RefHeading___Toc66168138)

8.4. Méthodologie statistique [57](#__RefHeading___Toc66168139)

8.4.1. Tolérance [57](#__RefHeading___Toc66168140)

8.4.1.1. Tolérance locale [57](#__RefHeading___Toc66168141)

8.4.1.2. Tolérance générale [57](#__RefHeading___Toc66168142)

8.4.1.3. Signes vitaux [57](#__RefHeading___Toc66168143)

8.4.1.4. Tolérance biologique [58](#__RefHeading___Toc66168144)

8.4.2. Immunogénicité [58](#__RefHeading___Toc66168145)

8.4.2.1. Abord qualitatif [58](#__RefHeading___Toc66168146)

8.4.2.2. Abord semi-quantitatif [58](#__RefHeading___Toc66168147)

8.4.2.3. Abord quantitatif [58](#__RefHeading___Toc66168148)

9. comite de surveillance [60](#__RefHeading___Toc66168149)

9.1. Comité scientifique [60](#__RefHeading___Toc66168150)

9.2. Comité indépendant [60](#__RefHeading___Toc66168151)

10. ethique et aspect reglementaire [60](#__RefHeading___Toc66168152)

10.1. Aspects éthiques et réglementaires [60](#__RefHeading___Toc66168153)

10.2. Amendement au protocole [61](#__RefHeading___Toc66168154)

10.3. Confidentialité des données [61](#__RefHeading___Toc66168155)

10.4. Assurance [61](#__RefHeading___Toc66168156)

11. confidentialite et indemnisation [62](#__RefHeading___Toc66168157)

11.1. Confidentialité [62](#__RefHeading___Toc66168158)

11.1.1. Confidentialité des données [62](#__RefHeading___Toc66168159)

11.1.2. Confidentialité des dossiers des volontaires [62](#__RefHeading___Toc66168160)

11.2. Indemnisation des sujets [62](#__RefHeading___Toc66168161)

12. REGLES DE PUBLICATION ET DE CESSION DES DONNEES [62](#__RefHeading___Toc66168162)

13. references bibliographiques [63](#__RefHeading___Toc66168163)

14. annexes [65](#__RefHeading___Toc66168164)

# 1. Justification de l’étude

## 1.1.Epidémiologie

Les estimations les plus récentes estiment qu’environ 40[34-46] millions de personnes sont infectées par le VIH dans le monde, 94% d’entre elles vivant dans les pays en développement (1). Durant l’année 2003, 5[4,2-5,8] millions de personnes ont été infectées par le virus et l’infection a été responsable de 3[2,5-3,5] millions de décès. Depuis le début de l’épidémie, 23 millions de personnes sont décédés du SIDA. L’infection par le VIH est la première cause de décès en Afrique (20,6% des décès sont liés au SIDA) et la quatrième cause de décès à l’échelon mondial. Alors qu’en Afrique, la prévalence de l’infection est croissante (au Bostwana, la séro-prévalence du VIH est proche de 40%), l’épidémie s’étend en Europe de l’Est, en Russie mais également en Asie en particulier en Inde, en Thaïlande, au Cambodge, en Chine et plus récemment au Viet Nam. Dans les pays industrialisés, l’introduction des traitements anti-rétroviraux a permis d’améliorer la durée de vie des patients mais leur efficacité et leur tolérance à moyen et long terme sont inconnues. Dans ces conditions, la mise au point d’un vaccin préventif contre l’infection par le VIH est plus que jamais prioritaire pour parvenir à contrôler l’épidémie.

## 1.2. Positionnement de l’étude

### 1.2.1. Etat de la recherche vaccinale contre le VIH

Depuis l’identification du VIH en 1984, la recherche vaccinale s’est heurtée à de nombreuses difficultés. Les premiers travaux se sont intéressés à induire une immunité humorale anti-VIH par la synthèse d’anticorps neutralisants (immunisation par des antigènes d’enveloppe du VIH (gp160, gp120) sous la forme de virus tué ou de protéines d’enveloppe recombinantes), mécanisme de protection de beaucoup de vaccins viraux actuellement commercialisés comme le vaccin contre la polio ou le virus de l’hépatite B. Cette approche s’est vue rapidement limitée par la grande variabilité et/ou l’inaccessibilité des protéines d’enveloppe comportant les sites de liaison aux anticorps neutralisants et la faible immunogénicité des régions conservées (2). Cependant des résultats récents obtenus sur le modèle macaque d’infection par un virus chimérique SHIV (virus simian exprimant les glycoprotéines d’enveloppe VIH-1) ont relancé l’intérêt de l’immunité humorale en démontrant que l’infection virale pouvait être évitée par la perfusion préalable d’anticorps neutralisants (3,4). Les travaux en cours s’intéressent à l’identification des immunogènes permettant l’obtention d’anticorps neutralisants à « large spectre » et pour ce faire à l’étude de la structure tri-dimensionnelle de l’enveloppe du VIH et des complexes formés lors de la fixation du VIH à la surface des CD4.

Le rôle des lymphocytes CD8+ dans le contrôle de l’infection par le VIH a rapidement été mis en évidence. *In vitro*, ils ont la capacité d’empêcher la réplication du VIH dans les cellules T CD4+. *In vivo*, l’apparition de lymphocytes T cytotoxiques (CTL) spécifiques du VIH précède le contrôle précoce et partiel de la réplication virale. Enfin, dans certaines situations cliniques des sujets exposés au VIH et non infectés (prostituées africaines, enfants nés de mères infectées par le VIH ou usagers de drogues), la présence de CTL CD8+ spécifiques du VIH est la seule trace d’une exposition au VIH : l’absence de virus chez ces sujets implique que les CTL sont capables d’empêcher la dissémination du virus dans l’organisme et d’en assurer l’éradication. Un vaccin efficace devrait donc être capable d’assurer au moins une réponse cellulaire CD8 spécifique mais également CD4 pour permettre la fonctionnalité des CTL (5). Le rôle de la réponse immunitaire cellulaire induite par la vaccination (lymphocytes T CD4+ et CD8+) a été montré chez le macaque dans des modèles d’infection par SHIV. L’immunisation des macaques par des vaccins ADN ou recombinants viraux (Modified Vaccinia Ankara, MVA, adénovirus) permet le maintien d’une charge virale plus basse que chez les animaux non vaccinés et le retard au développement de la maladie SIDA (6,7). Chez l’Homme, différents candidat-vaccins sont en cours d’évaluation chez les volontaires sains : les vaccins recombinants viraux (ALVAC-VIH, MVA, Adenovirus), les vaccins ADN et les lipopeptides du VIH seuls ou associés à un antigène T (toxine tétanique). Ces vaccins sont dirigés contre les protéines internes du VIH (gag, pol et nef) et devraient donc en cas d’exposition au VIH contrôler le niveau de la virémie sans pouvoir empêcher l’infection. Les premiers résultats chez l’homme mettent en évidence une bonne tolérance de ces produits et leur capacité à induire une réponse immunitaire cellulaire (lymphocytes T CD4+ et CD8+) spécifique du VIH chez environ 50% des volontaires.

### 1.2.2. Les lipopeptides

Les lipopeptides sont constitués de peptides synthétiques auxquels sont liées de façon covalente une ou plusieurs chaînes lipidiques. Ces constructions synthétiques permettent d’accroître l’immunogénicité des peptides synthétiques, en particulier la réponse cellulaire T cytotoxique, mais également T helper. La synthèse peptidique est maîtrisée et peut être optimisée. Elle permet de sélectionner les régions immunodominantes. Les lipopeptides ont montré une stabilité vis à vis de la chaleur et de la lumière dans des conditions adéquates et leur biodégradation est totale. Enfin, contrairement aux vecteurs viraux qui provoquent des réponses immunes dirigées contre le vecteur limitant la possibilité de rappels, les lipopeptides ne déclenchent pas ce type de phénomènes. On peut donc les utiliser sans perte de leur pouvoir antigénique. La capacité des lipopeptides à induire une réponse cellulaire a été montrée chez l’Homme dans le domaine des maladies infectieuses avec le modèle de l’hépatite B, de la syphilis et du VIH (8-13).

#### 1.2.2.1. Structures des lipopeptides du VIH

Quatre préparations lipopeptidiques monopalmitoylées (LIPO-5, LIPO-6 et LIPO-6T, LIPO-4 ou LPHIV1) ont été construites et testées chez l'Homme dans des essais de phase I-II. (tableau 1)

Tableau 1 : Structure des lipopeptides.

| Lipopeptides | Séquences peptidiques | Type de branchement à la chaîne lipidique |
| --- | --- | --- |
| LIPO-5 | Gag (aa 17-35 ; aa 253-284)  Pol (aa 325-355)  Nef (aa 66-97 ; aa 116-145) | C-terminal |
| LIPO-6 | Gag (aa 183-214 ; aa 253-284)  Nef (aa 66-97 ; aa 117-147 ; aa 182-205)  Env (V3, aa 303-335) | C-terminal |
| LIPO-6T | Gag (aa 17-35 ; aa 253-284)  Pol (aa 325-355)  Nef (aa 66-97 ; aa 116-145)  Toxine tétanique (TT 830-846) | C-terminal |
| LIPO-4 ou LPHIV1 | Gag (aa 77-85)  Pol-RT (aa 342-354), pol (aa 476-484)  Nef (aa 68-82)  Toxine tétanique (TT 830-843) | N-terminal |

#### 1.2.2.2. Tolérance des lipopeptides chez l’animal

Les études de toxicologie ont été effectuées chez la souris, le rat et le lapin avec les lipopeptides LPHIV1 et LIPO-6T (brochure investigateur).

#### 1.2.2.3. Expérience clinique avec les lipopeptides du VIH

Plusieurs études cliniques de phase I-II utilisant les lipopeptides du VIH, seuls ou en combinaison avec des vaccins recombinants viraux, ont été réalisées chez le volontaire et chez le patient infecté par le VIH traité par anti-rétroviraux. Elles sont résumées dans le

tableau 2.

Tableau 2 : Etudes cliniques utilisant les lipopeptides (LP) du VIH.

| Type de lipopeptides | Séquences peptidiques | ALVAC-VIH | Essais | Statut VIH | Nombre de sujets ayant reçu des LP | Statut |
| --- | --- | --- | --- | --- | --- | --- |
| LIPO-4 ou LPVIH1 | . Gag (aa 77-85)  . Pol (aa 476-484), Pol-RT  (aa 342-354)  . Nef (aa 68-82)  . Tetanus Toxin (TT 830-843) | - | ANRS VAC12 | VIH- | 15 | Terminé |
| LIPO-5 | . Gag (aa 17-35, aa 253-284)  . Pol (aa 325-355)  . Nef (aa 66-97 ; aa 116-145) | - | ANRS VAC10 | VIH- | 14 | Terminé |
| LIPO-6 | . Gag (aa 183-214 ; aa 253-284)  . Nef (aa 66-9; aa 117-147;  aa 182-205)  . Env (V3, aa 303-335) | -  vCP205/ vCP300  - | ANRSVAC04  ANRSVAC09  LIPTHERA | VIH-  VIH-  VIH+ | 28  8/15  24 | Terminé  Terminé  Terminé (11) |
| LIPO-6T | . Gag (aa 17-35 ; aa 253-284)  . Pol (aa 325-355)  . Nef (aa 66-97 ; aa 116-145)  . Tetanus Toxin (TT 830-846) | /vCP1452  -/vCP1452  vCP1433  vCP1433 | ANRS VAC10  ANRSVAC17*  ANRS093  ANRS095 | VIH-  VIH-  VIH+  VIH+ | 14/13  12/19  33  15 | Terminé  Terminé  Terminé (12)  Terminé  (13) |

*13 des 31 volontaires de VAC17 avaient déjà reçu des lipopeptides lors de leur participation à l’essai VAC10.

Dans tous ces essais cliniques, les lipopeptides du VIH ont été administrés par voie intramusculaire.

##### *1.2.2.3.1. Principales données d’immunogénicité des lipopeptides chez le volontaire sain non infecté par le VIH.*

L’essai ANRS VAC04 a évalué chez 28 volontaires non infectés par le VIH, les différents paramètres de la réponse immunitaire obtenus après immunisation par LIPO-6, mélange de 6 lipopeptides différents (3 lipopeptides contenant des fragments peptidiques d’une trentaine d’acides aminés dérivés de la protéine Nef, 2 fragments peptidiques de Gag et 1 fragment peptidique de l’Env, voir Tableau 2). Cette étude a montré la possibilité d’induire des réponses B et T multispécifiques du VIH. Les sujets vaccinés développent des anticorps capables de reconnaître les protéines du VIH correspondant aux fragments lipidiques Nef et Gag. Après 3 injections du candidat-vaccin LIPO-6, une réponse anticorps a été obtenue chez 25 (89%) des 28 volontaires. Une réponse cellulaire T CD4^+^ et CD8^+^ spécifique a été obtenue. Avec 3 injections du candidat-vaccin, une réponse proliférative de type CD4^+^ a été mise en évidence chez 79% des volontaires vaccinés et 54% des individus présentent une réponse CD8^+^ significative (14,15). Après une 4^ème^ injection, une augmentation significative de la fréquence d'individus répondeurs a été observée. Enfin, il a été montré que plus de 60% des individus ont maintenu une réponse à long terme (16).

Dans l’essai Biovector Therapeutics VAC12, 15 volontaires non infectés par le VIH ont été immunisés par LIPO-4 ou LPVIH1, mélange de 4 lipopeptides (1 lipopeptide contenant 1 fragment lipidique de Gag, 1 fragment lipidique de Pol, 1 fragment lipidique de Pol-RT et 1 fragment lipidique de Nef) en synthèse colinéaire avec un épitope T-CD4^+^ dit “ universel ” (peptide dérivé de la toxine tétanique ou peptide TT). Le peptide TT 830-843 est un épitope T capable de se lier à une grande variété de classe-II. Les résultats de la réponse immunitaire obtenue sont en cours d’analyse. La majorité des volontaires a été capable d'induire une réponse T-CD4+ puisque plus de 80% des volontaires vaccinés sont répondeurs. L'évaluation de la réponse T-CD8^+^ a été réalisée en utilisant les PBMC des volontaires vaccinés présentant un HLA compatible avec les peptides T-CD8^+^ inclus dans les lipopeptides. Ainsi, seules les données issues de 12 des 15 volontaires ont pu être exploités pour leur réponse T CD8+ induite. Trois volontaires présentaient un HLA ne permettant pas d'associer les épitopes T-CD8+ présents dans la préparation vaccinale. Après une injection du candidat-vaccin, 58% des volontaires présentent une réponse T CD8+ anti-VIH spécifique induite par le vaccin. Après 3 injections, le pourcentage de volontaires positifs pour cette réponse passe à 67%. Chez les individus ayant un typage HLA-A2 ou B7/B35, il a été montré que plus de 70% de ces sujets vaccinés ont été capables d'induire une réponse T-CD8+ anti-VIH après immunisation par LIPO-4. Parmi les volontaires de VAC12, 40% des sujets ont présenté au moins 2 fois une réponse positive au même peptide.

Les données d’immunogénicité des autres essais sont en cours d’acquisition et d’interprétation.

##### *1.2.2.3.2. Tolérance des lipopeptides chez l’Homme*

Jusqu’à présent, 197 volontaires ont participé à un essai utilisant des préparations lipopeptidiques, 125 volontaires et 72 patients infectés par le VIH avec au total 670 injections de lipopeptides ont été administrées. Il faut d’emblée noter la difficulté d’interprétation des réactions générales dans la mesure ou certains sujets ont reçu préalablement une ou plusieurs injections de vaccin viral recombinant de type ALVAC-VIH. Les données de tolérance ont fait l’objet d’un rapport (données non publiées). Les problèmes d’intolérance locale sont marqués par la survenue quasi-constante de douleurs au point d’injection avec la survenue d’une douleur de grade sévère chez environ 3% des patients. Les effets secondaires généraux sont habituellement peu sévères. Six effets secondaires généraux de grade III ou IV ont été observés: il s’agissait d’une fièvre dans 2 cas, d’arthralgie dans 1 cas, d’une uvéite dans 2 cas (2 volontaires ayant reçu préalablement des injections de l’ALVAC-VIH et des lipopeptides adjuvantés par le QS-21 ) et d’une asthénie dans 1 cas.

LIPO-4 (LPHIV1) a été administré dans l’essai VAC12. Dans cet essai, 15 volontaires ont été inclus, recevant chacun 3 injections du produit par voie intramusculaire. La tolérance a été évaluée à partir d’une échelle constituée de 3 grades : léger/ bénin, modéré et sévère. Les résultats de tolérance clinique et biologique de cet essai sont actuellement en cours d’analyse. Les premiers résultats montrent que la tolérance vis à vis des lipopeptides LPVIH-1 est satisfaisante. En effet aucun événement indésirable grave n’a été relaté. Parmi les 5 événements de grade sévère (voir tableau 3), seuls 2 événements ayant concernés deux sujets sont possiblement liés au produit. Parmi les 58 événements de grade modéré, seuls 34 événements, concernant 12 sujets (voir tableau 3), sont liés au produit (possible ou certain). Vingt cinq de ces 33 événements sont des douleurs au point d’injection et concernent 12 sujets. Toutefois aucune de ces douleurs n’est de grade sévère. Si l’on fait exception des douleurs au point d’injection, 8 événements de grade modéré et 3 de grade sévère ont été observés chez 7 volontaires .

Tableau 3 : Descriptif des événements, liés au produit (grade modéré ou sévère) observés dans l’essai VAC12.

| Nature de l’événement | **Grade modéré** | | **Grade sévère** | |
| --- | --- | --- | --- | --- |
|  | Nb événements | Nb de sujets | Nb événements | Nb de sujets |
| Céphalée |  |  | 1 | 1 |
| Arthralgie (épaule, hanche temporo-mandibulaire) | 5 | 4 | 1 | 1 |
| Douleur au point d’injection | 25 | 12 |  |  |
| Erythème | 1 | 1 |  |  |
| Contracture | 1 | 1 |  |  |
| Prurit | 1 | 1 |  |  |
| Lipothymie | 1 | 1 |  |  |
| **Total** | **34** | **12** | **2** | **2** |

### 1.2.3 Voies d’administration des lipopeptides

#### 1.2.3.1.Avantages et inconvénients des différentes voies d’injection (18)

La voie d’injection utilisée pour l’administration d’un vaccin va influer sur les épitopes reconnus par le système immunitaire et la réponse immunitaire elle-même, qu’elle soit de type cellulaire et/ou humorale (119).

La voie intramusculaire est la voie la plus fréquemment utilisée pour l’administration des vaccins. Elle est particulièrement recommandée pour les vaccins comportant un adjuvant qui, lorsqu’ils sont administrés par voie sous-cutanée ou intradermique sont responsables de réactions locales (irritation, induration, inflammation, granulome). Elle permet l’administration de volumes importants. En revanche, le muscle comportant peu de cellules immunitaires (cellules dendritiques, macrophages ou lymphocytes) n’est pas considéré comme un lieu optimal de présentation de l’antigène. Enfin, cette voie d’administration ne peut être utilisée en cas d’anomalies de la coagulation en raison du risque important de survenue d’un hématome au point d’injection.

La voie intradermique est particulièrement intéressante pour l’administration des vaccins. Le derme et l’épiderme constituent une barrière entre le milieu extérieur et l’hôte et intervient directement dans les défenses contre les agents pathogènes en contact avec la peau. En effet, le système immunitaire est bien représenté à la fois dans le derme et dans l’épiderme. Le dépôt des antigènes vaccinaux dans le derme permet un accès privilégié aux cellules présentatrices d’antigènes et aux zones d’échanges vasculaires et lymphatiques déterminantes pour la circulation des cellules immunocompétentes (20-22). Dès 1930, sont publiés des résultats mettant en évidence que de petites doses de vaccin contre la typhoïde administrées par voie intradermique assurent une meilleure immunogénicité et une meilleure tolérance que de plus fortes doses administrées par voie sous-cutanée. Plus récemment, il a été montré que la vaccination antirabique préventive ou curative pouvait utiliser des doses 5 à 10 fois plus faibles par voie intradermique par rapport à la voie d’injection classique intramusculaire, ce qui est d’une importance économique majeure dans les pays d’endémie rabique (23-24). De même plusieurs études ont mis en évidence l’efficacité de faibles doses de vaccin recombinant contre l’hépatite B administrées par voie intradermique (25-27). L’aiguille utilisée pour les injections par voie intradermique est de petite taille permettant une meilleure acceptabilité de l’injection et limitant les sensations douloureuses au moment de l’injection et dans les jours suivant l’injection. Une plus large utilisation de la voie intradermique, justifiée par une meilleure efficacité, est limitée par les difficultés pratiques qui peuvent être rencontrées dans certains cas. Cependant, des travaux récents s’intéressent au développement de nouveaux systèmes d’injection intradermique qui devraient faciliter ce mode d’administration (28).

#### 1.2.3.2.Voies d’injection utilisées pour l’administration des lipopeptides

Jusqu’à présent, les lipopeptides spécifiques du VIH testés dans le cadre du vaccin préventif ont toujours été administrés par voie intra-musculaire chez l’homme. Il faut cependant noter que la voie intra-dermique a été utilisée chez le macaque avec une tolérance locale et générale excellente. Des lipopeptides de *Trepanoma pallidum* ont été administrés par voie intra-dermique chez l’homme avec une bonne tolérance locale et générale. Dans cette étude, l’étude de la réponse locale par la méthode des « blisters » a permis de mettre en évidence une réponse immune cellulaire à la fois innée et adaptative (10).

### 1.2.4. Choix du type de lipopeptides et des doses de lipopeptides LPVIH1 pour l’essai.

Les lipopeptides choisis pour l’essai sont LIPO-4 ou LPVH1 comportant 4 séquences peptidiques du VIH : Gag (aa 77-85), Pol (aa 476-484), Pol-RT (aa 342-354) et Nef (aa 68-82) sur lesquelles est branché de façon colinéaire un fragment peptidique de la toxine tétanique (TT 830-843) qui a pour but de favoriser la réponse CD4. Ce candidat-vaccin pourrait être par ailleurs être utilisé dans le cadre de la vaccinothérapie chez des patients infectés par le VIH car il comporte peu d’épitopes CD4 du VIH et aurait donc l’avantage d’être peu stimulant sur la réponse VIH CD4+. Il permet de plus une analyse immunologique fine en terme de nombre de peptides répondeurs, de nombre de réponses et de la durée de la réponse car les différents épitopes sont bien définis.

Ces lipopeptides ont été utilisés dans l’essai Biovector Therapeutics ANRS VAC12 et les résultats sont en cours d’interprétation. Ils ont été injectés dans cet essai par voie intramusculaire à la dose de 500µg par peptide soit 2000µg au total dans un volume total de 0,5ml. Il a été décidé de garder cette posologie pour les volontaires qui recevront le candidat-vaccin par voie intramusculaire. A partir des données disponibles dans la littérature, l’hypothèse d’une immunogénicité accrue d’un facteur 5 en cas d’injection du candidat-vaccin par voie intradermique a été considérée. Cela implique alors d’injecter 100µg de chacun des peptides soit 500µg au total. Si on garde la même dilution que pour la voie intramusculaire, le volume à injecter est alors de 0,1ml, volume habituellement injecté par voie intradermique. Garder la même dilution pour les 2 voies d’injection présente l’avantage de conserver la même formation micellaire du produit pour les administrations par voie IM et ID.

# 2. objectifs de l’essai

## 2.1. Objectif principal

L’objectif principal de cet essai est d’évaluer la tolérance clinique (locale et générale) et biologique de LPVIH1 administré par voie intradermique et de la comparer à la voie intramusculaire chez des volontaires non infectés par le VIH.

## 2.2. Objectifs secondaires

Les objectifs secondaires sont :

- de comparer l’immunogénicité cellulaire de LPVIH1 administré par voie intramusculaire ou intradermique.

- d’explorer sur un sous-groupe de 6 volontaires la réponse immunitaire locale observée après immunisation par LPVIH1 administré par voie intradermique.

# 3. Schema de l’essai et methodologie

## 3.1. Schéma de l’essai

### 3.1.1. Schéma de l’essai

Il s’agit d’un essai multicentrique de phase IB sans bénéfice individuel direct, randomisé, stratifié et ouvert en deux groupes.

Tableau 3 : Schéma des injections de l’essai VAC16.

| Groupe | Voie d’administration | Dose de LPVIH1  (dose totale) | Sujets  (Total = 70) | Schéma d'immunisation en semaines | | | |
| --- | --- | --- | --- | --- | --- | --- | --- |
|  |  |  |  | 0 | 4 | 12 | 24§ |
| A | intramusculaire | 500µg  (2000µg) | 35 | LPVIH1 | LPVIH1 | LPVIH1 |  |
| B | intradermique | 100µg  (400µg) | 35 (dont 6§) | LPVIH1 | LPVIH1 | LPVIH1 | LPVIH1§ |

§ : pour les 6 sujets participant à la sous-étude.

Dans le bras d’administration intradermique, la survenue d’un événement de grade 4 ou la survenue chez au moins 3 volontaires d’événements locaux (au site d’injection) et/ou généraux de grade 3, pouvant être liés au produit, sera recensée en temps réel et entraînera l’arrêt de l’essai. De même la survenue chez un des sujets d’un événement de grade 3 entraîne chez ce sujet l’arrêt définitif des immunisations.

Chaque sujet recevra 3 injections de LPVIH1 (à S0, S4 et S12). Les injections seront administrées par voie intramusculaire (n=35) ou intradermique (n=35). Les volontaires seront ensuite suivis jusque S24+12J.

Dans la sous-étude physiopathologique de la réponse immunitaire locale pour les volontaires ayant reçu le candidat-vaccin par voie intradermique, 6 sujets recevront une quatrième injection à S24 et une biopsie cutanée sera réalisée 48H après pour évaluer la réponse des lymphocytes CD8+ en ELISPOT-IFN-γ.

Dans la mesure du possible, les injections seront réalisées par le même investigateur pendant toute la durée de l’essai.

### 3.1.2. Procédures d’attribution des bras d’injection

Les sujets seront randomisés dans l’un des deux groupes de l’essai en fonction de la voie d’injection. Une stratification est prévue selon que le typage HLA (réalisé à S-6) est susceptible d’induire une réponse immune CD8 à 0-1 peptide, à 2-5 peptides ou à 6-11 peptides (voir tableaux ci-dessous). Une répartition homogène des 3 strates dans les différents bras est nécessaire pour l'étude de l'immunogénicité.

Une liste de randomisation par strate sera préparée par la statisticienne de l’étude.

Nombre de peptides reconnus par molécule HLA.

| **HLA** | **PEPTIDES POTENTIELS** |
| --- | --- |
| A2 | **4** |
| A3 ou A11 | **1** |
| A24 | **2** |
| B7 ou B35 | **6** |
| B44 | **1** |

**Exemple :**

| **HLA combiné** | **PEPTIDES POTENTIELS** |
| --- | --- |
| A2 et B7 ou B53 | **10** |
| A2,A24 B7 | **11** |
| A3/A11 | **1** |
| Autres HLA non cités | **0** |

| **Strate*** |  |
| --- | --- |
| I ( de 0 à 1 peptide) | Non exploitable pour l’immunologie |
| II (2 à 5 peptides) | Immunologie exploitable |
| III (6 à 11 peptides) | Immunologie exploitable |

* Pour la répartition des volontaires dans les 3 strates, la validation sera faite par H. Gahéry-Ségard.

## 3.2. Population de l’essai

Les volontaires sont recrutés depuis juin 93 par l'Agence Nationale de Recherches sur le SIDA (ANRS) dans la population générale après annonces publiques de demande de candidatures aux essais cliniques faites par voies de presse et radiophonique. Les sujets volontaires auront suivi le processus de recrutement mis en place par l'ANRS et auront satisfait aux entretiens de sélection médicale et médico-psychologique (consultation médicale et bilan biologique complet, information et consentement écrit de participation à ce réseau de volontaires, motivations et évaluation des risques de contamination ou d'abandon de l'essai). Le processus de sélection élaboré par l'ANRS en vue de constituer un réseau de volontaires désireux de participer à des essais cliniques vaccinaux contre le VIH a été approuvé par le CCPPRB de Paris-Cochin.

### 3.2.1. Critères d’inclusion

Les sujets inclus dans l'essai sont des :

- sujets âgés de 21 à 55 ans.

- volontaires du réseau ANRS.

- femmes qui ne devront pas être enceintes ou allaitantes et devront accepter de poursuivre ou d'entreprendre, pendant toute la durée de l'essai, un moyen de contraception considéré comme efficace par l’investigateur, ou devront présenter une stérilité documentée ou être ménopausées.

- sujets présentant un examen clinique normal.

- sujets affiliés à un régime de la Sécurité Sociale ou bénéficiaires d’une assurance-maladie.

- sujets ayant signé le consentement éclairé.

- sujets pouvant recevoir le produit à l’essai indifféremment par l’une des deux voies d’administration.

### 3.2.2. Critères de non-inclusion

Les sujets qui ne pourront être inclus dans l'essai sont ceux :

- avec un risque identifiable de contracter une infection VIH d'après les entretiens de recrutement initial.

- ayant participé à un autre essai clinique relatif à l'infection à VIH ou concernant un immuno-modulateur, participant à un autre essai sans bénéfice individuel direct ou étant dans la phase d'exclusion d'un essai clinique relatif à un autre produit.

- sujets ayant eu une vaccination dans le mois précédant la première injection ou nécessitant une vaccination pendant la durée de l’essai.
- sujets ayant fait un don de sang dans les 2 mois précédant la visite de pré-inclusion.
- ayant des antécédents d'allergie alimentaire de l'adulte documentée, de syndrome de Lyell, de Stevens-Johnson, d'asthme grave décompensé.
- ayant un eczéma généralisé actif
- ayant un urticaire chronique évolutif en cours.
- sujets ayant eu une uvéite ou ayant un risque de développement d’une uvéite et présentant des maladies auto-immunes, des maladies infectieuses (syphillis, maladie de Lyme et mycobactérioses évolutives) ou la sarcoïdose.

- suivant un traitement immuno-modulateur au long cours (interférons ou autres cytokines ou immunostimulants) ou l'ayant suivi dans les 6 mois précédant l'essai (chimiothérapie, corticothérapie, radiothérapie)

- ayant un traitement médicamenteux en cours susceptible de modifier la réponse immunitaire (AINS) et/ou responsable de perturbations biologiques pouvant gêner l'interprétation des résultats de tolérance.

- ayant une affection médicale grave évolutive ou des antécédents de maladies auto-immunes (syndrome de Bechet, spondylarthrite ankylosante, polyarthrite rhumatoïde, maladie de Bowen, psoriasis et syndrome de Reiter, connectivite, maladie neurologique démyélinisante, colite inflammatoire…) ou ayant laissé des séquelles cliniques et/ou biologiques persistantes

- ayant reçu une transfusion de sang total ou des dérivés sanguins plasmatiques dans les 6 mois précédant l'essai

- ayant été traités par des hormones hypophysaires d'extraction
- ayant montré des anomalies cliniquement significatives aux tests biologiques standards, pouvant gêner l'interprétation des résultats de tolérance
- ayant une réponse positive aux tests spécifiques VIH (ELISA, WB et antigènémie p24) et aux tests sérologiques VHB, VHC, HTLV-1 et syphilis.
- Ayant un taux d’Hb > 12,5 g/dl pour les femmes et > 13,5g/dl pour les hommes
- Ayant une férritinémie en dehors de la normale.
- présentant des conditions psychosociologiques et géographiques incompatibles avec l'essai (population instable, mobilité géographique)

### 3.2.3. Attribution des numéros d’identification des sujets

A la semaine S-6, un code d'identification de 5 lettres construit à partir des initiales des nom et prénom (3 et 2 lettres respectivement) est attribué au sujet.

A S0, après vérification des critères cliniques d'éligibilité, les sujets recevront un numéro d’inclusion (numéro attribué de manière séquentielle sous la forme « 0YY »-« 16xx » , 0YY est le numéro du centre (voir tableau 4), 16 est le numéro de l'essai ANRS et xx (de 01 à 70) est le numéro du dossier du volontaire) suivi de leur code d'identification.

La randomisation se fera au plus tôt 5 jours ouvrés, avant la visite S0, par la CRO après que l’investigateur lui ait adressé par fax les feuillets du cahier d’observation de la visite de pré-inclusion ainsi que les résultats biologiques. Parallèlement, l’investigateur faxera les résultats du typage HLA au Dr. H. Gahery de l’U567 afin de valider l’appartenance du volontaire à une strate donnée.

| **Numéro du centre** | **Centre** |
| --- | --- |
| **049** | Hôpital Tenon, Paris |
| **054** | HEGP, Paris |
| **056** | Hôpital Cochin, Paris |
| **072** | Hôpital Sainte Marguerite, Marseille |
| **076** | Hôpital de Nantes, Nantes |
| **085** | Hôpital Purpan, Toulouse |

Tableau 4 : Attribution des numéros des centres

### 3.2.4. Test de grossesse

Toutes les femmes en âge de procréer devront présenter un test de grossesse négatif lors de la visite de pré-inclusion à S-6 (β-HCG plasmatique). Ces femmes devront ensuite accepter de poursuivre ou d'entreprendre, pendant toute la durée de l'essai, un moyen de contraception considéré comme efficace par l’investigateur.

### 3.2.5. Thérapies antérieures et concomitantes

Tout traitement médicamenteux pris par un sujet à l'inclusion et au cours de l’essai devra être mentionné sur le cahier d’observation. La DCI (dénomination commune internationale), la dose journalière totale, la voie d'administration, les dates de début et de dernier jour de la prise, ainsi que l’indication pour laquelle ce traitement a été prescrit devront être reportées sur le cahier d’observation et dans les documents source.

Aucun traitement au long cours ayant une activité immuno-modulatrice n'est admis pour la poursuite de la vaccination. L'arrêt de la vaccination chez des sujets ayant pris un tel traitement fera l'objet d'une discussion entre les investigateurs et l’investigateur coordonnateur. Toute prescription doit être retranscrite dans le cahier d’observation et documentée dans le document source.

Si un autre vaccin que celui de l’étude est administré durant l’essai, l'investigateur devra noter le nom commercial, le numéro de lot et la date d’administration dans le cahier d’observation à l’endroit prévu à cet effet ainsi que dans le document source. L’arrêt de la vaccination sera discutée entre l’investigateur et l’investigateur coordonnateur.

## 3.3. Bénéfices pour les sujets/ risques potentiels

### 3.3.1. Bénéfices pour les volontaires

Cet essai clinique est sans bénéfice individuel direct pour le volontaire. Il n'existe aucune garantie que les préparations vaccinales administrées au volontaire induisent une réponse immunitaire protectrice contre l'infection par le VIH-1. Il est donc essentiel que le volontaire vacciné continue d'éviter tout comportement à risque susceptible d'entraîner une contamination par le VIH.

### 3.3.2. Risques potentiels

Certains volontaires ayant reçu des préparations vaccinales ont développé une séropositivité au test ELISA, test couramment utilisé dans le dépistage des individus pour l'infection à VIH. Cette séropositivité peut persister plusieurs années. Ceci peut représenter un risque social d'apparaître "séropositif", mais aussi un risque psychologique lié à cette séropositivité vaccinale. Cependant, il est possible très facilement de différencier cette "séropositivité" vaccinale de la séropositivité de l'infection à VIH-1, par un test spécifique (Western Blot), prouvant ainsi que la séropositivité est le résultat de la vaccination et non de l'infection à VIH-1. De même, la mise en évidence du VIH lui même (« charge virale ») par technique de biologie moléculaire (PCR) est dans ce cas négative. Les lipopeptides LPVIH1 ne contenant pas de peptide d’enveloppe, cette éventualité est peu probable. Dans l’essai VAC 12, aucun des 15 volontaires ayant reçu LPVIH1, n’a présenté une séropositivité au test ELISA dans les suites de l’essai.

Afin d’éviter tout désagrément social au volontaire, il lui sera remis une carte plastifiée (style carte de club) portant au recto sa photographie et son numéro d'identification dans l'essai (code sous la forme « 0YY »-« 16xx » : 0YY est le numéro de centre, 16 est le numéro de l'essai ANRS, xx (de 01 à 70) est le numéro du dossier du volontaire, et au verso, par exemple dans le cas du centre 056, les mentions "RESEAU-VOLONTAIRES, Hôpital Cochin - ANRS, 27, rue du Faubourg St. Jacques, 75679 Paris Cedex 14, numéro de téléphone répondeur téléphonique 24 h / 24 et un message personnalisé en fonction du médecin de garde de 9 h à 17 h". Il figurera aussi le logo de l'ANRS sans mention des acronymes et la formule bilingue Français/Anglais "En cas d'urgence médicale, contacter le numéro et/ou l'adresse indiqués". Cette procédure permettra au médecin d'obtenir les informations confidentielles relatives au sujet avec l'accord de celui-ci. L’investigateur sera informé le plus rapidement possible.

Il est concevable de façon théorique comme dans toute vaccination, que la réponse immunitaire engendre des phénomènes biologiques d'auto-immunité. Cependant, dans aucun des essais cliniques français ou nord-américains (plusieurs centaines de volontaires à l'heure actuelle) il n'a été observé de maladies auto-immunes déclenchées par la vaccination anti-VIH.

De même, il existe un risque théorique que le vaccin puisse faciliter l’infection en cas d’exposition au VIH. Enfin, on ne peut pas exclure que les sujets, ayant reçu le candidat-vaccin à l’essai, aient dans l’avenir une réponse modifiée lors d’une vaccination par un vaccin anti-VIH efficace.

Dans le bras d’administration intradermique, la survenue d’un événement de grade 4 ou la survenue chez au moins 3 volontaires d’événements locaux (au site d’injection) et/ou généraux de grade 3, pouvant être liés au produit, sera recensée en temps réel et entraînera l’arrêt de l’essai. De même la survenue chez un des sujets d’un événement de grade 3 entraîne chez ce sujet l’arrêt définitif des immunisations.

Le déroulement de cet essai réclame de nombreuses prises de sang, soit un total de 8 prélèvements (ou de 9 prélèvements pour les sujets participant à la sous-étude) de S-6 à S24+12J sur les 32 semaines de l'essai. Les quantités prélevées n'excèdent pas un total de 1036 ml et 1026 ml sur les 32 semaines de l’essai respectivement pour les femmes et les hommes (schéma synoptique, p5) qu’ils participent ou non à la sous-étude. Le dosage de la ferritine est prévue lors de la pré-inclusion dans l’essai et une supplémentation par fer sera proposée si nécessaire afin de remplir les conditions d’inclusion. Dans le cas où une supplémentation serait nécessaire, une deuxième visite de pré-inclusion sera réalisée, après 3 mois de traitement avec un bilan biologique allégé comprenant l’hématologie, la biochimie dont la ferritine. Ce bilan supplémentaire augmentera le volume de sang prélevé de 18 ml pour les hommes et de 23 ml pour les femmes. La ferritinémie sera suivie tout au long de l’étude afin de proposer une supplémentation par fer éventuelle.

En fin d'essai, il sera rappelé explicitement au sujet que celui-ci ne pourra pas participer à un autre essai clinique de recherche biomédicale pendant une période d'exclusion de 4 mois à dater de la fin du présent essai. Ils seront également informés du fait qu’ils ne pourront pas faire de dons de sang, de tissu ou de moelle durant l’essai puis pendant la période d’exclusion et jusqu'à négativation de leurs tests ELISA VIH et Western blot VIH 1et 2 (sauf pour les bandes existantes avant toute vaccination).

## 3.4. Arrêt prématuré de l’essai

L'essai pourra être interrompu :

1. en cas de survenue, dans le bras d’administration intradermique, d’un événement de grade 4 ou la survenue chez au moins 3 volontaires d’événements locaux (au site d’injection) et/ou généraux de grade 3, pouvant être liés au produit,
2. pour des raisons administratives
3. dans le cas où l'on disposerait de nouvelles données sur le produit à l'essai,
4. sur le conseil du promoteur, des investigateurs, du comité indépendant et/ou du CCPPRB.

Si l'essai est arrêté prématurément ou suspendu, le promoteur devra informer les investigateurs des centres, les Autorités Réglementaires et l’investigateur coordonnateur devra aviser le CCPPRB de la raison de l'arrêt ou de la suspension.

## 3.5. Calendrier de l’essai

**Pré-inclusions** : mai- octobre 2004

**Inclusions** : juin-novembre 2004

**Durée de l’essai** : 32 semaines à partir de la pré-inclusion

**Fin de l'essai** (dernière visite de suivi du dernier sujet) : fin juin 2005

**Analyse statistique à la fin de l’essai**: Christine DURIER (SC10, Hôpital Paul Brousse)

**Rapport clinique et immunologique final** : CRO

## 3.6. Schéma des vaccinations et plan des prélèvements

Voir schéma synoptique p5.

**4. produits de l’essai**

## 4.1. Caractéristiques du mélange de lipopeptides LPVIH1

### 4.1.1. Description des lipopeptides (LPVIH1 )

Le vaccin est constitué des quatre lipopeptides: gag 77-85, pol-RT 342-354, pol 476-484, nef 68-82. Ceux-ci correspondent à des séquences exprimées dans les protéines *gag*, *pol* et *nef* du virus VIH-1 de sous-type LAI. Ces peptides sont associés chacun de façon colinéaire à un épitope T helper de la toxine tétanique (TT) 830-843 branché en N-terminal à un acide gras monopalmitique. Les quatre lipopeptides ont été obtenus par synthèse chimique et présentent individuellement une pureté supérieure ou égale à 74%, les 26% restants sont des produits secondaires de séquences très proches des peptides (délétions, modifications) ou des résidus de réactifs de production (eau, TFA, acétonitrile, chloride…), non dangereux pour les volontaires aux doses retrouvées.

### 4.1.2. Composition des lipopeptides

Chaque dose vaccinale de LPVIH1 se présente en flacon qui contient le mélange lyophilisé des 4 lipopeptides. Les doses administrées, selon le groupe de randomisation, sont les suivantes:

Principes actifs ( LPVIH1) :

| **lipopeptides** | **Mélange lipopeptidique** | |
| --- | --- | --- |
|  | **Groupe A** | **Groupe B** |
| gag 77-85  pol-RT 342-354  pol 476-484  nef 68-82 | 500 µg  500 µg  500 µg  500 µg | 100µg  100µg  100µg  100µg |
| Dose totale | 2000µg | 400µg |
| N° de lot | BT358038A | BT358038A |

Les doses vaccinales seront reconstituées avec une solution glucosée à 5%.

## 4.2. Etiquetage et conditionnement

Tous les produits auront une double étiquette détachable où apparaissent le nom du produit et les mentions légales. Cette étiquette autocollante est à coller sur le document source.

| Utilisation sous stricte surveillance médicale:  Médicament pour essai clinique. | **LPVIH1**  Essai ANRS VAC16 1 flacon 1 dose  Voie intramusculaire ou intradermique  Conserver à –20°C  Promoteur: ANRS , 101 rue de Tolbiac 75 013 PARIS  Tél : 01.53.94.60.00.  Fournisseur: | Lot : BT358038A  Date de réanalyse : 01/2003 |
| --- | --- | --- |

## 4.3. Conditions d’expédition et de stockage

Le stock initial des lipopeptides LPVIH1 est conservé en dessous de -20°C (congélateur) à la pharmacie coordinatrice de l’hôpital Cochin.

Un envoi des produits sera prévu pour chaque centre avant le début de l’essai et les produits seront alors stockés dans les mêmes conditions à la pharmacie de l’hôpital du centre.

A la réception des produits, le pharmacien vérifiera qu’il n’y a pas eu de problème de rupture de la chaîne du froid pendant le transport. En cas de problème, elle devra avertir le chef de projet immédiatement. Le pharmacien du centre investigateur datera et signera l’accusé de réception. L’original sera gardé pour archivage à la pharmacie et une copie sera renvoyée à la pharmacie coordinatrice et au chef de projet du centre coordonnateur de l’étude.

La température du congélateur devra être vérifiée et documentée quotidiennement sur le formulaire correspondant (Cf. modalités pratiques cliniques) pendant tout l'essai.

La solution glucosée à 5% (ampoules) sera stockée à température ambiante dans le service de chaque centre.

## 4.4. Préparation et précautions d'utilisation des lipopeptides

La technicienne/infirmière, munie d’une ordonnance « produit », en duplicata, ira chercher le produit à la pharmacie. Le vaccin (2 mg de mélange équipondéral de chacun des quatre lipopeptides) est à reconstituer extemporanément dans 0,6 mL de solution glucosée à 5%. Au moment de l’administration, on ne sortira pour la séance vaccinale, qu’un seul flacon LPVIH1 et qu’une ampoule de solution glucosée à 5%.

La reconstitution extemporanée s'effectue dans les conditions habituelles d'asepsie :

- nettoyer à l’alcool avec une gaze stérile le bouchon du flacon de lipopeptides et de diluant (prendre soin de ne laisser aucune trace d’alcool sur le bouchon). Injecter lentement à l’aide d’une seringue pré-remplie, 0,6 ml d’une solution glucosée à 5%, dans le flacon de lipopeptides.

- homogénéiser le flacon ainsi reconstitué doucement par retournement manuel une dizaine de fois avant utilisation.

-reprendre 0,5ml de produit ainsi reconstitué dans une seringue de 2 ml montée sur une aiguille stérile (23G5/8) pour les administrations par voie IM et 0,1 ml de produit dans une seringue de 1 ml montée sur une aiguille stérile (26 GA3/8) pour les administrations par voie ID.

- placer sur un plateau stérile et injecter dans les 5 minutes 0,5 ml pour les administrations par voie IM et 0,1 ml pour les administrations par voie ID.

## 4.5. Comptabilité des produits

Les produits devront être stockés dans un endroit sûr (congélateur avec température contrôlée, et alarme en cas de défaillance). La responsable de la gestion des produits, remplira la fiche de comptabilité des produits lors de la dispensation des flacons de produit pour chaque sujet.

Parallèlement, l’investigateur remplira le « formulaire de dispensation et de réconciliation des produits » inséré dans les modalités pratiques cliniques, après chaque administration.

## 4.6. Retour des produits inutilisés et/ ou entamés

Les produits inutilisés pendant l’essai resteront sur le lieu de stockage, la chaîne du froid devant être maintenue et documentée .

Les produits, amenés sur le lieu prévu de l’administration, mais non injectés seront renvoyés, sur le lieu de stockage du centre (pharmacie). La décongélation du produit sera précisément documentée sur l’ordonnance « produit » conservée au centre et sur la copie destinée à la pharmacie ainsi que sur l’emballage du produit. L’ensemble des produits, restant sur le lieu de stockage de chaque centre, seront rapatriés en toute fin d’étude vers la pharmacie coordinatrice, après vérification par l’ARC de la CRO de la comptabilité. Le rapatriement se fera avec le formulaire « retour des produits non utilisés».

Les produits entamés seront jetés dans les poubelles pour produits biologiques du centre et détruits selon la procédure de destruction de l’Hôpital correspondant.

# 5. organisation

## 5.1. Centres et personnels impliqués dans l’essai

Il s’agit d’un essai multicentrique avec 6 centres cliniques incluant des volontaires. Ces centres travailleront en collaboration avec la pharmacie de leur hôpital. Les analyses immunologiques seront réalisées dans le laboratoire d’immunologie cellulaire du Département d’Immunologie de l’Institut Cochin. L’étude sera supervisée par un chef de projet localisé au centre coordinateur, le Centre Cochin-Pasteur d’essais vaccinaux, situé au pavillon Saint Louis de l’hôpital Cochin. La gestion des données, le monitoring et data management seront assurés par une CRO. L’analyse statistique sera réalisée par Christine Durier (SC 10, INSERM). L’ensemble des centres et des personnes impliquées dans cet essai est présenté dans l’annexe 7.

## 5.2. Déroulement des visites

### 5.2.1. Visite de pré-inclusion

La visite de pré-inclusion a lieu à S-6 (1 mois et demi avant la première vaccination). L'investigateur propose l'étude au sujet et lui demande une lecture approfondie de la note d'information sur l'essai et du consentement éclairé tripliqué. Il répond à toutes les questions du sujet et s’assure de sa compréhension. Il fait signer le consentement éclairé au sujet (l'original étant conservé par l'investigateur, une copie étant remise au sujet et une autre conservée, sous enveloppe scellée, par le promoteur).

Il procède alors à :

- - la vérification des critères d'inclusion et d'exclusion
- - un interrogatoire précis sur les antécédents médicaux et vaccinaux,
- - la vérification des rappels de vaccinations usuelles,
- - un examen clinique
- - la notification de la mairie de naissance uniquement sur la fiche « liste de screening et d’inclusion des sujets de l’étude » archivée dans le dossier investigateur,
- - des prélèvements de sang et d'urines pour bilan biologique et immunologique de pré-inclusion (voir Tableau synoptique p5)
- - la vérification de la prise par tout sujet féminin en âge de procréer d'une contraception efficace tout le long de l'essai,
- - l'attribution au sujet d'un code d'identification à 5 lettres.
- - l'inscription sur le fichier national des volontaires.

Deuxième visite de pré-inclusion :

Pour les sujets ayant une Hb anormale ou une ferritinémie basse lors de la pré-inclusion, une deuxième visite de «pré-inclusion » leur sera proposée après 3 mois de traitement par Tardyferon®. Lors de cette visite sera effectué un prélèvement sanguin sur lequel seront réalisés les examens biologiques suivants :

- NFS, plaquettes
- TP/TCA , VS
- Urée, créatinine, ionogramme, calcémie, phosphorémie, glycémie, protéines totales, ASAT/ALAT, phosphatases alcalines, CPK, bilirubine totale, gamma GT, ferritine
- β-HCG pour les femmes.

### 5.2.2. Randomisation

La statisticienne du SC10 génère 3 listes de randomisation (1/strate, fonction du typage HLA) comprenant un numéro d'inclusion du volontaire (1601 à 1670) et le groupe de randomisation.

La procédure de randomisation des volontaires de l'étude est la suivante:

• l’investigateur envoie par fax :

- à la CRO une copie des feuillets du CRF correspondant à la visite de pré-inclusion et des résultats biologiques rendus anonymes (dont le typage HLA) correspondants dès réception de ceux-ci

- au Dr. H. Gahery du laboratoire U567 (fax : 01.40.48.83.52), les résultats du typage HLA qui confirmera, par fax, à la CRO la strate attribuée au volontaire.

• la CRO contrôle les critères d'admissibilité au plus tôt 5 jours ouvrés avant la visite de S0, cela est effectué au moyen d’un programme informatisé adapté aux critères définis dans le protocole avec une revue préalable des critères d'inclusion et de non-inclusion par l'ARC senior et/ou le Chef de Projet de la CRO,

• la CRO, en tenant compte de la strate à laquelle appartient le volontaire, transmet le numéro d'inclusion du volontaire selon l'ordre chronologique d'inclusion, au CPCC et au centre investigateur (clinicien) par fax au plus tôt 5 jours ouvrés avant la visite S0. Sur ce fax, la CRO précisera, s’il s’agit d’un sujet randomisé dans le groupe ID, si la sous-étude peut être proposée au sujet (strates 2 et 3).

Tout problème pouvant donner lieu à un refus de randomisation doit être discuté avec le CPCC. Toute décision de refus de randomisation doit être argumentée par écrit. Tous les documents relatifs à la randomisation sont classés par la CRO et consultables en cas de besoin et notamment: copie des fax/courriers aux investigateurs et liste de randomisation.

### 5.2.3. Participation à la sous-étude

#### 5.2.3.1. Critères pour l’inclusion dans la sous-étude

Il sera proposé aux sujets randomisés dans le groupe d’administration par voie ID de participer à la sous-étude. Plusieurs critères seront préalablement vérifiés à la visite S24:

- l’investigateur ne proposera l’étude que s’il estime que la tolérance a été bonne (aucun événement de grade 3) au cours des premières injections.
- l’investigateur devra s’assurer que le sujet appartient aux strates 2 ou 3
- le sujet devra avoir donner son accord et signer le consentement après avoir pris connaissance de la lettre d’information.

#### 5.2.3.2. Participation à la sous-étude

- L’investigateur proposera la sous-étude aux sujets inclus dans le groupe d’administration par voie ID lors de la visite S12 et lui remettra la lettre d’information N°2 afin que le volontaire lui fasse part de sa décision à la visite S14.

- L’investigateur devra prévenir, par fax, entre les visites S14 et S20 et si et seulement si les feuillets du CRF concernant les visites S-4 à S14 inclus ont été transmis à la CRO, qu’il prévoit d’inclure un sujet dans la sous-étude en précisant le numéro et le code identifiant du sujet, le numéro de centre, la date prévue pour la visite S24 et S24+2J et si ces dates ont été approuvées par le laboratoire de H. Gahery.

- La CRO devra donner, dès réception de l’ensemble des documents, l’accord pour la participation du sujet à la sous-étude. En cas de désaccord, soit par nécessité de compléments d’information soit parce que le nombre des 6 sujets aura été atteint, la CRO devra immédiatement prévenir l’investigateur par fax.

- Lorsque le nombre de 6 sujets inclus dans la sous-étude est atteint, la CRO devra avertir immédiatement tous les centres par fax.

- A la fin de la visite d’inclusion d’un sujet dans la sous-étude (S24), l’investigateur devra prévenir la CRO et le laboratoire de H. Gahery, en spécifiant le numéro de centre, le code identifiant du sujet et son numéro d’inclusion initial, la date réelle de l’inclusion à la sous-étude et la date programmée pour la visite S24+48H.

### 5.2.4. Visites d’inclusion et de vaccinations

Trois visites donneront lieu à l’administration de vaccin, aux dates S0 (±7j), S4 (±7j), S12 (±7j). Pour les sujets participant à la sous-étude, une quatrième visite avec injection intradermique est prévue à S24 (±14j).

Avant l'injection (visite d’inclusion à S0 uniquement):

- vérification des critères d’inclusion et d’exclusion dans l’étude, (cf. §3.2.1 et 3.2.2.)
- remise d'un thermomètre et d'une réglette et explication de leur utilisation.
- attribution au sujet d’un numéro d’inclusion
- remise de la carte ANRS

Avant l'injection (toutes visites de vaccination):

- examen clinique complet (pression artérielle, fréquence cardiaque [après 10 min de repos], température buccale)
- vérification des critères autorisant la poursuite de la vaccination : test urinaire de grossesse négatif le jour de l'injection, examen clinique normal et bilan biologique sanguin et urinaire ne montrant pas d'anomalie grave.
- prélèvements de sang et d'urines (voir Tableau synoptique p5)
- dispensation de la dose vaccinale par la pharmacie

L’injection :

- L’injection par voie IM de la préparation vaccinale se fera dans la région deltoïde.
- L’injection par voie ID dans la face externe du bras, perpendiculairement à la peau. L’injection devra être réalisée sur une surface où la peau ne présente pas une forte pilosité, ni de cicatrice.

**→** Pour l’administration par voie ID, la conduite à tenir sera la suivante :

- si l’administration a échoué mais que le produit n’a pas pénétré dans l’organisme, on retire rapidement l’aiguille et on recommence l’administration par voie intradermique dans le bras opposé. L’investigateur devra alors documenter dans le dossier source que l’injection a du être recommencée. Dans ce contexte, l’investigateur doit donc conserver le flacon contenant la dilution du produit dans le cas où il devrait refaire l’injection.
- L’investigateur devra noter sur le CRF la présence ou non de papule. (la présence ou non d’une papule ne présage en rien de la réponse immune).

En cas de contre-indication temporaire à la vaccination (affection intercurrente : syndrôme grippal …, médicaments associés contre-indiqués dans l’essai) ou de contre-temps de la part du sujet, la vaccination pourra être retardée. Si le délai est supérieur à 7 jours, l’investigateur devra discuter au cas par cas avec l’investigateur coordonnateur de l’essai. Si un sujet manque une séance de vaccination, on reprend le calendrier vaccinal là où on s’était arrêté après accord de l’investigateur coordonnateur de l’essai. Aucun sujet ne sera écarté de l'analyse statistique et tout événement sera consigné dans le CRF.

Après l'injection (Toutes visites de vaccination):

- le volontaire reste au repos pendant 30 minutes.
- examen clinique local et général pratiqué 30 min après l'injection pour les voies IM et ID avec prise de la pression artérielle, de la fréquence cardiaque et de la température buccale.
- remise d'un carnet d'auto-surveillance que le volontaire remplira après chaque injection
- prises de la température buccale à nouveau par le sujet le soir à 18 h puis matin et soir pendant les 2 jours suivant l'injection, et le matin du troisième jour ou lors d'une sensation fébrile; les températures enregistrées seront notées par le volontaire sur le carnet d'auto-surveillance.
- rappel au sujet de la date de la prochaine visite et de la nécessité d'une contraception efficace pour les femmes en âge d’avoir des enfants.
- Prescription d’antalgiques en cas de douleurs.

### 5.2.5. Visites de suivi

Cinq visites de suivi sont prévues, aux dates S2 (±3j), S6 (±3j), S14 (±3j), S24 (±14j) comportant chacune:

- un examen clinique avec prise de la pression artérielle, de la fréquence cardiaque (après 10 min de repos) et de la température buccale
- des prélèvements de sang et d'urines (voir Tableau synoptique p5)
- une vérification des données recueillies par le sujet sur son carnet d'auto-surveillance depuis la visite précédente et retranscription de ces données sur le cahier d'observation
- une recherche des EIs pouvant être survenus depuis la visite précédente.
- rappel au sujet de la date de la prochaine visite et de la nécessité d'une contraception efficace pour les femmes en âge d’avoir des enfants.
- Une attention toute particulière devra être apportée en cas de survenue de manifestation à type d’arthralgie, en particulier de localisation temporo-mandibulaire. Dans ce cas, des explorations complémentaires comportant le dosage du complément (C3, C4, CH50), la sérologie anti-tétanique (à partir du sérum) et une recherche de protéinurie devront être effectuées en temps réel lors de l’apparition de la symptomatologie clinique et à la disparition des signes cliniques.
- Une visite supplémentaire de suivi à S24+12J permettra de rendre les résultats des examens biologiques et de renseigner le volontaire quant à son statut sérologique vis à vis du VIH.

### 5.2.6. Visites concernant la sous-étude

#### 5.2.6.1. visite de vaccination

Cette visite (S24) se déroulera comme les autres visites de vaccination (cf §5.5.3.) mis à part les points suivants:

- l’investigateur aura préalablement vérifié que le sujet appartient à la strate 2 ou 3 et que la tolérance aux premières injections était bonne puis proposé la sous-étude au sujet lors de la visite S12. A la visite S24, l’investigateur rediscutera de la sous-étude avec le sujet et fera signer le deuxième consentement en triplicata, une fois que le sujet l’aura comprise et acceptée.
- un prélèvement d’urine sera effectué, chez les femmes, afin de réaliser un test de grossesse urinaire avant l’injection.
- l’injection par voie intradermique se fera à la face **interne** du bras.

Si le produit n’est pas injecté de façon strictement intradermique, c’est à dire qu’il n’a pas pénétré dans l’organisme, l’investigateur pourra refaire l’injection à la face interne du bras opposé. L’investigateur devra donc conserver le flacon de dilution du produit au cas où il devrait refaire l’injection. Toutefois l’investigateur devra le documenter dans le dossier source.

- l’investigateur remettra au sujet le carnet d’auto-surveillance N°4.

#### 5.2.6.2. visite de la biopsie

Cette visite a lieu 48H après la quatrième injection (S24+2J). La biopsie sera réalisée sur le site même de l’injection comme suit:

- La biopsie sera réalisée dans des conditions de stérilité maximum sur une zone inflammatoire si elle existe ou sinon sur la zone d’injection.
- Bien nettoyer la peau à la chlorhexidine, faire une anesthésie locale avec 1 ml de xylocaïne adrénalinée à 1% (aiguille orange).
- Attendre quelques minutes avant de désinfecter à nouveau (chlorhexidine) puis prélever au punch de 4 mm une carotte de peau. Déposer la biopsie dans une compresse stérile imbibée de sérum physiologique stérile dans un pot stérile (de type stérilin ou pot à ECBU) sur lequel, une étiquette identifiant le volontaire (code) aura été apposée.
- Recoudre ensuite la peau avec un fil Ethicrin 4.0.
- Nettoyage de la peau tous les jours au savon de marseille.

#### 5.2.6.3. visite de suivi

Une visite de suivi, à S24+12J, au cours de laquelle sont prévus :

- un examen clinique
- le retrait du fil du point de suture
- la récupération du dernier carnet d’auto-surveillance (N°4)
- le rendu des résultats des examens biologiques

## 5.3. Procédures pour l’obtention et le traitement des échantillons

### 5.3.1. Prélèvement des échantillons de sang

Des échantillons de sang sont collectés aux différentes visites pour permettre la réalisation sur site des examens biologiques standards (hématologie, biochimie, tests d'auto-immunité ...) et des sérologies virales. Vingt millilitres de sang seront prélevés dans des tubes secs à chaque visite pour la sérothèque et la sérologie tétanique. Dix autres millilitres de sang seront prélevés dans des tubes EDTA aux visites S-6, S2, S6, S14 et S24 pour la constitution d’une plasmathèque.. Une charge virale pourra le cas échéant être évaluée rétrospectivement sur plasma congelé. Des volumes de 100 ml de sang total sont collectés et adressés par coursier à l’U567 à l'hôpital Saint Vincent de Paul, aux visites S-6, S0, S2, S6, S14 et S24 pour permettre l'isolement des cellules mononucléées du sang périphérique. Pour les sujets participant à la sous-étude un volume de 50 ml sera prélevé à S24 et à S24+J2. Ces PBMC (lymphocytes B et T, macrophages et cellules dendritiques) permettent l'évaluation de l’immunité cellulaire spécifique.

En cas de Western Blot positif à une protéine d’enveloppe, et dès réception de ce résultat, une antigénémie p24 sera réalisée rétrospectivement sur un tube de la sérothèque.

### 5.3.2. Acheminement et préparation des échantillons en vue de l’analyse immunologique cellulaire périphérique et locale.

#### 5.3.2.1. Acheminement

Les tubes de sang frais devront être placés dans des boîtes isothermes avec du coton pour maintenir une température de 20°C. Le transport de la boite jusqu’au Département d’Immunologie de l’Institut Cochin devra respecter les normes en vigueur pour le transport de sang. A chaque boîte seront joints un bon de transport ainsi que l’ordonnance destinée au Département d’Immunologie.

Le prélèvement devra arriver le matin avant midi à l’attention du Dr.Hanne Gahery/ Département d’Immunologie de l’Institut Cochin en évitant le vendredi. Le laboratoire ne pourra recevoir qu’un nombre limité de prélèvements par jour, c’est pourquoi une copie du planning général des volontaires de chacun des centres devra être envoyée au laboratoire pour faciliter la gestion des prélèvements. En cas de modification de la date d’un rendez-vous, contacter le laboratoire d’Immunologie pour vérifier que cela est possible. Le délai maximal pour la conservation des cellules étant de 24 heures, les sujets des centres localisés en province devront impérativement être prélevés en fin d’après midi notamment pour les centres pour lesquels le transport ne pourrait être réalisé dans la matinée.

A cette fin, il faudra donc organiser les horaires de rendez-vous en fonction de l’organisme de transport et de la durée du transport.

Le transport de la biopsie (à S24+48H) se fera en même temps que la poche de sang, prélevée lors de cette même visite, et se fera dans les mêmes conditions que citées ci-dessus.

#### 5.3.2.2. Préparation des cellules mononucléées au laboratoire d’Immunologie

Les cellules sont séparées des autres constituants sanguins par gradient de densité grâce à une solution dite "Ficoll". Le sang est dilué au demi dans de l’eau physiologique, puis déposé très délicatement sur la solution de Ficoll. Les tubes sont centrifugés à 400 g pendant 20 minutes à 20°C sans frein. L'anneau de globules blancs mononucléés est aspiré avec précaution, lavé et centrifugé pendant 15 minutes à 300 g afin d'éliminer le Ficoll. Le culot est ensuite resuspendu dans du milieu adéquat. Les cellules obtenues peuvent alors être utilisées immédiatement ou congelées pour une utilisation ultérieure, typage HLA (S-6), constitution de lignées cellulaires B-EBV (S-6), immunité cellulaire spécifique (S0, S2, S6, S14, S24 et S24+2J).

### 5.3.3. Préparation de la sérothèque et de la plasmathèque

##### 5.3.3.1. Centrifugation

- Les prélèvements sanguins destinés à la sérothèque seront laissés à température ambiante entre 30 minutes et 2 heures puis ils seront centrifugés pendant 10 minutes à 2000 T/min.

- Les tubes de sang (prélevés sur EDTA) destinés à la plasmathèque seront aussitôt centrifugés pendant 5 minutes à 2000T/min à 4°C.

##### 5.3.3.2. Aliquotage

Les tubes destinés soit à la sérothèque soit à la plasmathèque seront aliquotés et stockés selon le même schéma toutefois, le plasma devra respecter une chaîne du froid à 4°C (dans la glace) jusqu’à sa congélation à –80°C.

La manipulation d'un tube lors de l'aliquotage doit être réalisée sujet par sujet afin d'éviter tout risque d'inversion. Aussi, la séquence de l'aliquotage sera la suivante:

- Après centrifugation, la personne responsable de l'aliquotage ne prend dans la centrifugeuse que les tubes correspondant à un même sujet afin de les aliquoter.
- Elle dispose alors sur un portoir uniquement le nombre de tubes Nunc nécessaires à l'aliquotage et colle les étiquettes complétées dessus.
- Elle réalise l'aliquotage sur l’ensemble du volume de plasma ou de serum disponible (donc le nombre de tubes sera variable). Des aliquots de 900µl seront réalisés pour la sérothèque et de 500µl pour la plasmathèque.
- Elle élimine alors les tubes Nunc déjà étiquetés qui n'auraient pas été remplis par manque de sérum ou de plasma.
- Elle complète la liste récapitulative soit de la sérothèque soit de la plasmathèque (numéro et initiales du sujet, date du prélèvement, nombre d'aliquots réalisé, date et heure d'aliquotage) pour le sujet correspondant.
- Elle indique des commentaires éventuels sur la qualité des prélèvements (hémolysés, contaminés ...).
- Le tube suivant de sang ne peut être sorti de la centrifugeuse qu'une fois toutes ces étapes terminées.

##### 5.3.3.3. Conditions de stockage

Les aliquots devront être congelés à -20°C ou –80°C pour la sérothèque et la plasmathèque. La température devra être vérifiée et documentée sur le formulaire correspondant (Cf. modalités pratiques laboratoire) pendant tout l'essai.

##### 5.3.3.4. Conditions de transport

Après la fin de l’étude, la sérothèque et la plasmathèque seront rapatriées de chacun des centres vers un site commun de conservation par un transporteur garantissant les bonnes conditions de sécurité et de maintien de la chaîne du froid. Chaque envoi d’échantillons sera accompagné de la liste récapitulative des prélèvements correspondants ainsi que du bordereau d’envoi. Une copie de ces documents devant être archivée dans le dossier investigateur du centre.

### 5.3.4. Préparation des échantillons de la biopsie en vue de l’analyse immunologique.

La biopsie sera découpée en 2 ou 3 fragments de façon stérile avec un bistouri. Chaque petit morceau sera déposé dans un godet d’une plaque 24 puits avec 2 ml de milieu RPMI-SAB 10%. 100 UI/ml d’IL-2 seront ajoutés à J3, J7 puis 1 fois par semaine. Les cellules seront observées et comptées 2 fois par semaine à partir de la deuxième ou troisième semaine. Lorsqu’un nombre suffisant de cellules est obtenu, les cellules seront utilisées pour la réalisation d’un ELISPOT IFN-γ et d’un phénotypage lymphocytaire CD3/CD56, CD4/CD8, TRα/β,γ/δ.

## 5.4. Cahiers d’observations et recueil des données

Toutes les informations seront notées par l'investigateur ou une personne désignée par l’investigateur dans les cahiers d'observations fournis. Cette personne désignée sera inscrite sur le formulaire « délégation des tâches ». Les cahiers d'observations seront remplis au stylo noir et signés par l'investigateur. Toute donnée manquante devra être justifiée. Les données incorrectes devront être barrées par un seul trait. Les corrections devront être paraphées et datées par l'investigateur. Le fluide correcteur ne doit pas être utilisé.

## 5.5. Carnets d’auto-surveillance

Un carnet d'auto-surveillance sera remis à chaque sujet lors de chaque visite de vaccination (3 carnets ou 4 carnets pour les sujets participant à la sous-étude/ voir schéma synoptique p5). Ils permettront de noter les réactions locales et événements systémiques survenus jusqu'à la visite suivante. L'évaluation des réactions locales consistera en la surveillance du site d'injection (une réglette millimétrée sera fournie à cet usage). L'évaluation des événements systémiques consistera en la prise de température orale (un thermomètre électronique sera fourni pour l’essai) et le report des réactions comme les maux de tête, les douleurs musculaires, etc. Il sera précisé dessus d’utiliser en première intention un antalgique de niveau I (paracétamol, et/ou salicylés ou AINS) pour le traitement les céphalées et/ou les douleurs. L’utilisation d’antalgiques de niveau 2 utilisés à tort entraînerait la déclaration des céphalées en événement de grade 3. Ces données journalières seront recueillies à chaque visite, discutées avec le sujet et transcrites dans le CRF. Toute consultation médicale ou hospitalisation, ainsi que les traitements reçus seront également notés et transcrits dans le CRF.

## 5.6. Conditions de sortie d’essai

### 5.6.1. Arrêt du traitement

La poursuite de la série de vaccinations chez les sujets se fera après examen détaillé des données de tolérance clinique et biologique relatives à la précédente injection et après discussion avec l’investigateur coordonnateur. L'investigateur devra interrompre la série de vaccinations en cas d'apparition en cours d'essai d'un événement représentant une contre-indication définitive:

- - la survenue d'une grossesse,
- - un comportement à risque vis à vis de l’infection par le VIH,
- - la contamination par le VIH-1 ou le VIH-2,
- - la survenue d'un événement indésirable grave ou sévère pouvant être lié au produit de l’essai,
- - l’apparition d'un des critères de non-inclusion (cf § 3.2.2.).

Les sujets sortis d’essai après inclusion et vaccination (quelque soit le nombre de vaccinations reçues) auront un suivi allégé c’est à dire la visite deux semaines après la dernière injection et à S24. Lors de ces visites, un examen clinique des volontaires est réalisé et une prise de sang est effectuée selon les modalités de la visite décrite dans le protocole (poche de sang comprise).

### 5.6.2. Abandons

A tout moment, un sujet peut décider de ne plus participer à l'essai. Dans ce cas, l'investigateur devra s'assurer que la raison de l'abandon n'est pas liée à la survenue d'un Evénement Indésirable. La cause des abandons en cours d'essai sera précisée dans le cahier d'observations dans la partie "Bilan de fin d'essai" et notifiée dans le document source.

## 5.7. Procédures en cas de perdu de vue

Tout sujet qui ne se présente pas à une visite prévue et qui ne donne pas de nouvelles devra faire l'objet d'une recherche approfondie avant d’être considéré comme perdu de vue. Des efforts seront entrepris pour limiter au maximum le nombre de sujets perdus de vue. Dans la mesure du possible, les causes amenant à considérer un sujet perdu de vue seront recherchées, documentées et rapportées dans le CRF et dans le document source.

## 5.8. Classification de fin d’essai

Définitions:

Arrêt par décision de l’investigateur : une observation est notée "Arrêt par décision de l’investigateur" lorsque l’investigateur décide de retirer le sujet de l’essai pour raison médicale (sécurité du sujet en cause), raison personnelle (il estime que le sujet n’est pas à même de poursuivre), ...

Abandon : une observation est notée "Abandon" lorsqu’un sujet inclus dans l’essai décide, de sa propre initiative, de ne pas poursuivre dans l’essai

Perdu de vue : le sujet n’a pas été retrouvé malgré les recherches de l’investigateur

Décès.

## 5.9. Monitoring, audit et archivage

### 5.9.1. Organisation générale

Le chef de projet de la CRO assure le monitoring de l’essai auprès des investigateurs pour le compte de l’ANRS (promoteur de l’essai). Celui-ci prendra contact régulièrement avec l’investigateur, pour accéder aux données consignées dans les cahiers d’observations.

Toutes les informations recueillies au cours de l’essai doivent être soigneusement colligées afin de permettre l’exploitation des résultats.

La responsabilité du moniteur est de vérifier régulièrement l’adhésion au protocole et le remplissage correct des cahiers d’observations.

L’investigateur accepte de coopérer avec le moniteur afin de résoudre les problèmes éventuels soulevés par ce dernier à l’issue de chaque visite de monitoring.

### 5.9.2. Visites de monitoring

La fréquence des visites de monitoring est établie comme suit :

Une visite de Mise en Place dans le mois qui suit la réception de la totalité du matériel de l’essai. L’investigateur ne pourra inclure son premier volontaire qu’après que le moniteur ait effectué cette visite.

Une première visite de monitoring le plus rapidement possible après la première inclusion dans chaque centre dans un délai maximal de 15 jours.

Des visites de monitoring dont le nombre sera adapté en fonction du recrutement du centre.

Au moins un appel téléphonique entre deux visites

Une visite de clôture à la fin de l’essai.

Dans le cas où l’investigateur ne procéderait à aucune inclusion, les visites de monitoring pourront être plus espacées et/ou remplacées par un contact téléphonique.

Le monitoring consistera en :

a- Vérification des documents sources :

L’investigateur s’engage à mettre les documents sources relatifs au volontaire à la disposition du moniteur lors des visites de monitoring. Celui-ci vérifiera chaque fois que nécessaire, les données consignées dans les cahiers d’observations par rapport aux documents sources.

b- Vérification pour tous les cahiers d’observations :

- de l’existence de tous les volontaires inclus en s’assurant qu’il y ait un dossier médical pour chaque volontaire dans lequel figurent :

- le nom et prénom, le sexe, la date de naissance et la mairie de naissance

- la mention de la pré-inclusion dans l’essai et la date de pré-inclusion.

- de l’existence d’un consentement informé, signé pour chaque volontaire et du deuxième consentement pour les sujets participant à la sous-étude.
- des critères d’inclusion et d’exclusion.
- des événements indésirables en particulier pour la voie d’administration intradermique. La validation des événements indésirables de grade supérieur ou égal à 3 sera réalisée en temps réel afin de discuter de l’interruption éventuelle des inclusions.

c- Vérification des résultats biologiques archivés en cas d'incohérence

### 5.9.3. Consignes et cahiers d’observations

Un ensemble de consignes sera rédigé par la CRO et communiqué aux médecins investigateurs. Ces consignes figureront dans le cahier d’observations en regard de chaque page à compléter.

Un cahier d’observations sera attribué à chaque volontaire, identifié par son numéro et son code d’identification uniquement. Les fiches auto-copiantes en triplicata seront remplies à partir du dossier clinique par le médecin investigateur ou son représentant nommément désigné au fur et à mesure des visites. Le troisième feuillet restera dans le CRF qui sera conservé par l’investigateur. Les 2 feuillets détachables, accompagnés de copies des résultats d’examens cliniques ou biologiques rendus anonymes par le code et le numéro du volontaire, seront récupérés par l’ARC en charge de l’essai. Le premier feuillet (original) sera donné au promoteur en fin d’essai et le second servira au codage et à la saisie des données. Par ailleurs, les fiches décrivant un événement indésirable grave ou une progression immunologique ou clinique seront faxées au département pharmacovigilance de l’ANRS aussitôt après la survenue ou la découverte de l’événement.

### 5.9.4. Gestion des données

Après recueil et validation, les données cliniques consignées sur les cahiers d'observations et/ou les autres résultats d'analyse des échantillons sérologiques ou biologiques prévus au protocole seront intégrés dans le système interne de gestion des données cliniques. Pour tout jeu de données, un contrôle de qualité et la mise en oeuvre de programmes de vérification logique et/ou de contrôle de cohérence des données seront appliqués systématiquement afin de détecter erreurs ou omissions. Après intégration de toutes les corrections sur l'ensemble complet des données, la base de données correspondante sera verrouillée et sauvegardée avant exploitation pour analyse statistique. Toutes les étapes de ce processus seront contrôlées par la mise en place individualisée de mots de passe confidentiels et/ou de sauvegardes régulières afin de maintenir un accès approprié aux bases de données et de garantir leur intégrité.

### 5.9.5. Audit, Inspection

Afin d’évaluer la qualité de l’essai et conformément aux Bonnes Pratiques Cliniques, chaque investigateur s’engage à accepter :

L’audit éventuel de l’essai par le promoteur directement ou avec son autorisation exprès par d’autres organismes.

L’inspection de l’essai par les Autorités de Santé qui y sont autorisées.

### 5.9.6. Archivage

Les médecins investigateurs archiveront et conserveront dans un dossier investigateur pendant au moins 15 ans après la fin de l'étude les documents suivants relatifs à l'étude :

- version actualisée du protocole, des annexes et amendements éventuels.

- cahiers d'observations.

- consentements éclairés des volontaires inclus*.

- le registre des volontaires (liste confidentielle des volontaires de l’essai qui fait le lien entre le volontaire, son identification dans l’essai et son dossier hospitalier)*.

- correspondance relative à l'essai.

(*) : Ces documents sont archivés dans une enveloppe scellée de façon à préserver la confidentialité durant toute la période d’archivage.

Les dossiers sources des volontaires devront également rester accessibles pendant tout ce temps.

Par ailleurs, la CRO devra retourner au promoteur les documents relatifs à l’essai notamment, les consentements éclairés des volontaires qui sont stockés dans une enveloppe scellée mentionnant le code 5 lettres du volontaire et son numéro d’inclusion. Le promoteur archivera et conservera tous les documents relatifs à l’essai conformément aux recommandations des Bonnes Pratiques Cliniques. Ces documents (protocole, amendements, documentation, approbations et tous les autres documents concernant l’essai y compris les données sur les événements indésirables, le rapport final, les certificats d’audits) doivent toujours figurer dans le dossier de référence de l’essai.

# 6. evenements indesirables

Tout événement indésirable grave sera déclaré au service de pharmacovigilance de l’ANRS à l’aide des feuillets figurant dans le CRF.

## 6.1. Définition et déclaration d’un événement indésirable grave

### 6.1.1. Définition

**Evénement indésirable (EI) :** Un événement indésirable désigne toute manifestation nocive et non recherchée, subie par une personne participant à une recherche biomédicale, quelle que soit la cause de cette manifestation.

**Evénement indésirable grave (EIG)**: Un événement indésirable grave est un événement susceptible d’être dû à la recherche et provoquant :

- un décès

- une menace vitale immédiate

- une incapacité ou une invalidité cliniquement significative, temporaire ou permanente

- toute anomalie biologique de grade III ou IV (échelle ANRS)

- une hospitalisation ou une prolongation d’hospitalisation

- une anomalie congénitale

Il s’agit de tout événement dont la survenue ne peut raisonnablement être attribuée à une cause indépendante des conditions de la recherche. D’autres cas d’événements indésirables ne répondant pas à la définition de gravité pourront faire l’objet d’une déclaration immédiate. Il s’agit d’événements potentiellement graves selon le jugement de l’investigateur ou d’événements indésirables inattendus.

**Evénement indésirable inattendu**: événement indésirable non défini en nature, fréquence et sévérité dans la brochure investigateur du produit à l’essai.

**Relation de causalité**: L’évaluation de la relation de causalité entre le traitement et l’événement indésirable grave d’une part et entre la recherche et l’événement grave d’autre part, sera faite initialement par l’investigateur sur la fiche de déclaration : **0** : sans relation, **1** : relation possible, **2** : relation probable, **3** : relation certaine.

### 6.1.2. Déclaration des événements indésirables graves

Dispositions réglementaires : afin de respecter la réglementation en vigueur concernant la déclaration des événements indésirables graves aux Autorités de Santé, l’investigateur s’engage à documenter l’événement, à respecter les délais de notification, à fournir toutes les informations nécessaires à l’analyse de cet événement.

L’investigateur adresse *dès qu’il en a connaissance* par fax à la CRO les feuillets de déclaration et la documentation de l’événement ainsi que la relation de causalité avec le vaccin. La CRO devra en informer immédiatement le promoteur (ANRS : Dr. Annie Métro ; fax : 01.53.94.60.02)

Le promoteur s’engage à informer, *dès qu’il en a connaissance*, l’Agence Française de Sécurité Sanitaire des Produits de Santé en leur déclarant tout événement grave susceptible d’être lié au produit ou dû à la recherche ; le promoteur s’engage également à informer les Autorités de Santé de tout arrêt prématuré de la recherche en indiquant le motif de cet arrêt.

**En cours d’essai**: Tout événement indésirable grave doit être déclaré à la CRO par l’investigateur quelle que soit sa relation de causalité avec le(s) traitement(s) de l’essai ou la recherche, *dès qu’il a connaissance* des 5 éléments suivants : identification du protocole, du volontaire, de l’investigateur, de la nature de l’EIG et du traitement suspect.

Afin de permettre à la CRO de déclarer *immédiatement* l’événement au promoteur, l’investigateur doit adresser **immédiatement pour les décès et en cas de menace vitale, dans les 24heures pour les autres** , par tout moyen à sa disposition [téléphone suivi d’une confirmation écrite, télécopie, courrier électronique], les documents cités ci-dessous à la CRO (Tél : - Fax : ).

- la fiche de recueil initial de l’événement indésirable grave (annexe 5), située en annexe du cahier d’observation,

- les copies des résultats de laboratoire ou des comptes rendus d’examens ou d’hospitalisation renseignant l’événement grave, sans omettre de rendre les documents anonymes et d’inscrire le numéro et le code du volontaire.

Puis l’investigateur remplira au plus tôt, dans les 5 jours ouvrables ou 7 jours calendaires la fiche de déclaration complémentaire de l’EIG (annexe 5) située en annexe du cahier d’observation. Ce document sera signé par l’investigateur et envoyé par fax ou courrier urgent à la CRO qui fera suivre ce document au promoteur.

**Après la fin de l’essai** : Tout EIG susceptible d’être lié à la recherche et survenant dans les 30 jours après la fin de l’essai pour le volontaire doit être déclaré selon la même procédure et les mêmes délais que les EIG survenant en cours d’essai.

Tout EIG susceptible d’être lié à la recherche de l’essai doit être déclaré par l’investigateur au promoteur quel que soit son délai de survenue. Il s’agit par exemple des événements graves pouvant apparaître à grande distance de l’exposition au traitement tels que les cancers, ou même les événements survenants sur la génération suivant celle exposée.

## 6. 2. Conduite à tenir en cas d’événement indésirable

- Une attention toute particulière devra être apportée dans le cas d’apparition d’arthralgie. En effet, en cas de survenue de manifestations évoquant une maladie sérique (en particulier en cas d’arthralgies temporo-mandibulaires), des explorations complémentaires comportant la recherche de complexes immuns circulants, le dosage du complément (C3, C4, CH50) et une recherche de protéinurie devront être effectuées en temps réel concomitamment aux signes cliniques et après disparition de la symptomatologie clinique.
- tous les événements (cliniques et biologiques) de grade égal ou supérieur à 3 pouvant être liés au produit seront rapportés en temps réel et déclarés soit :

- en EIG s’il s’agit d’un événement de grade 4 ou d’un événement biologique de grade 3. Cette déclaration sera faite à la CRO selon la procédure décrite dans le chapitre 6. - à l’aide de la fiche « Déclaration des événements locaux ou généraux de grade 3 pouvant être lié au produit » s’il s’agit d’événements cliniques de grade 3 (locaux ou généraux) sur laquelle la date de survenue, l’intensité, la nature de l’événement et l’action entreprise seront décrites (annexe 9). Cette fiche sera faxée à la CRO qui sera chargée de la faire suivre le plus rapidement possible au chef de projet du centre coordonnateur et faxée à l’investigateur coordonnateur qui constitue avec le co-investigateur le comité de validation saisi pour arrêter l’essai si 3 volontaires ont été concernés par ce type d’événement. Le sujet, qui aura présenté un événement de grade 3, ne devra plus être immunisé mais il effectuera les visites à S+J14 suivant la dernière immunisation reçue et à S24 conformément à celles décrites dans le protocole (poche de sang comprise).

## 6. 3. Conduite à tenir en cas de survenue de grossesse

Dès connaissance de la grossesse, l’investigateur en charge du suivi du volontaire adresse par fax au chef de projet de la CRO la fiche standard de recueil des données initiales de la grossesse (annexe 6) qui lui a été remise lors de la visite de mise en place de l’essai dans le dossier investigateur. La CRO transmettra le plus rapidement cette fiche à la pharmacovigilance de l’ANRS. La maternité prévue pour l’accouchement doit être précisée sur cette feuille de recueil. Toute grossesse détectée lors de la visite de pré-inclusion sera un critère de non inclusion et un critère de sortie d’essai si elle est détectée en cours d’essai.

A l’issue de la grossesse, le service de pharmacovigilance de l’ANRS – 101, rue de Tolbiac – 75 013 Paris – envoie au médecin de la maternité une fiche standard de recueil (Annexe 6) des données finales concernant la grossesse qu’il devra compléter et renvoyer à la pharmacovigilance de l’ANRS.

# 7. CRITERES D’EVALUATION

## 7.1. Critère principal d'évaluation

### 7.1.1. Définition

Le critère principal d’évaluation est la comparaison du nombre d’événements cliniques (locaux et systémiques) et biologiques de grade > 2 (à l’exception des douleurs au point d’injection), survenant après l’injection de LPVIH1, selon les 2 voies d’administration: intramusculaire ou intradermique.

### 7.1.2. Paramètres de tolérance à mesurer

#### 7.1.2.1 Tolérance clinique locale

Les critères d'évaluation de tolérance locale sont appréciés par l'examen clinique local, l'interrogatoire du sujet sur les sensations perçues au lieu d'injection et par les notifications du sujet sur son carnet d'auto-surveillance les 3 jours suivant l’injection. Toutes les réactions locales au point d’injection sont recueillies sur le cahier d'observation. Les réactions locales (érythème, induration, douleur, œdème et nodule) seront prélistées dans le carnet d'auto-surveillance et recueillies systématiquement pendant trois jours après chaque immunisation, puis lors de leur survenue jusqu'à la visite suivante :

On évaluera leur taille, mesurée en cm, et leur intensité (échelle ANRS, annexe 4) .

#### 7.1.2.2. Tolérance clinique générale

Les critères d'évaluation de tolérance clinique générale sont appréciés par l'examen clinique (comportant la mesure de la pression artérielle systolique et diastolique, de la fréquence cardiaque et de la température buccale effectuée 30 minutes après l’injection), l'interrogatoire du sujet sur les sensations générales perçues après l'injection et par les notifications du sujet sur le carnet d'auto-surveillance. Toutes les données sont recueillies sur le cahier d'observation. Les réactions générales, clairement définies, dans le carnet d'auto-surveillance seront recueillies systématiquement pendant cinq jours après chaque immunisation, puis lors de leur survenue jusqu'à la visite suivante. Sera notée systématiquement la survenue des événements suivants : éruption cutanée, asthénie, fièvre, hypotension, céphalées, nausées, vomissements, diarrhée, myalgies, arthralgies. L’intensité de ces événements sera évaluée grâce à l’échelle ANRS (annexe 4). La température sera mesurée grâce à un thermomètre et tout épisode fébrile sera à noter sur le carnet d’auto-surveillance même si cet épisode ne persiste pas plus de 12H.

#### 7.1.2.3. Surveillance clinique immédiate

Un examen clinique local et général sera pratiqué dans les 30 minutes après chaque injection réalisée quelque soit la voie d’administration. Il comportera un examen de la zone d'injection, une prise unique de la pression artérielle après 10 minutes de repos (pressions systoliques et diastoliques exprimées en mmHg), de la fréquence cardiaque (exprimée en battements par minute) et de la température buccale.

#### 7.1.2.4. Tolérance biologique

Les critères d'évaluation de tolérance biologique sont appréciés par l'analyse comparative des résultats des examens biologiques effectués en début d’essai, à J14 après chaque injection de vaccin soit à S2, S6, S14, ainsi qu’à S24. (cf tableau synoptique p5)

## 7.2.Critères secondaires d’évaluation

### 7.2.1. Définition

Les critères secondaires d'évaluation sont les événements cliniques (locaux et généraux) et/ou biologiques de degré 1 et 2, la réponse CD4 proliférative au vaccin et la capacité des lymphocytes T CD8+ à sécréter de l’IFN-γ en présence de peptides spécifiques (tests ELISPOT IFN-γ) dont l'évaluation sera faite à S2, S6 et S14.

### 7.2.2. Méthode de mesure des critères secondaires

 L'étude de la **réponse proliférative des lymphocytes T helper** sera effectuée sur cellules fraîches par le laboratoire d’immunologie du Département d’Immunologie de l’Institut Cochin (Jean Gérard Guillet - Hôpital COCHIN). Les résultats sont exprimés en coups par minute (cpm), cpm nets (test-témoin) dont le seuil de positivité est > 3000 cpm nets et en index de stimulation (IS) rapport test/témoin dont le seuil de positivité est ≥ 3.

 L’étude **des cellules CD8+** sera réalisée rétrospectivement sur une population de PBMC congelés. Après une infection virale (non chronique) et une vaccination, la fréquence des lymphocytes T CD8+ spécifiques de l’antigène est souvent trop faible pour permettre l’analyse directe par des méthodes classiques comme le test de CRT (Chrome Release Test). Des expansions cellulaires sont nécessaires ; les lymphocytes T sont restimulés plusieurs fois in vitro (de 3 à 5 fois) en présence de l’antigène. Ce procédé peut induire un biais quantitatif ou qualitatif sur la réponse recherchée. Un test sensible a donc été mis en place, l’ELISPOT (enzyme-linked-immunospot) qui détecte jusqu’à une cellule spécifique sécrétant de l’IFN-γ. Cette technique permet d’évaluer de faibles fréquences de lymphocytes CD8+ spécifiques, *ex vivo* sans restimulation à partir de cellules mononucléées isolées du sang. Pour valider de faibles fréquences de lymphocytes T spécifiques, il est possible de faire une seule stimulation *in vitro* en présence de l’antigène ou du peptide optimum, ce qui permet d’amplifier les effecteurs circulants « mémoires » et de confirmer un résultat *ex vivo*. Seront étudiées les réponses des lymphocytes T CD8+, *ex vivo* sans restimulation et *in vitro* après une seule stimulation. Pour l’établissement des lignées, les cellules congelées sont stimulées en direct par des pools de petits peptides (7 maximum) ou avec des pools de peptides témoins (Influenza, EBV, …). Chaque peptide CD8+ spécifique est incubé à 1 µg/ml. De l’IL-2 recombinante humaine est ajoutée à J3 et J7. L’ELISPOT est effectué à J12. Une lecture automatisée est utilisée.

Le critère retenu est un critère dichotomique en positif/négatif de réponse au candidat vaccin (capacité des CD8+ à sécréter de l’IFN-γ en présence des peptides présents dans le mélange LIPO-4 par ELISPOT IFN-γ; le sujet est considéré **répondeur** s’il présente une réponse dirigée **contre au moins un peptide, à deux temps différents** pour le même peptide.

La détermination des réponses sera effectuée au moins par la méthode visuelle et par une méthode statistique objective, qui sera sélectionnée parmi les méthodes en cours de développement, y compris dans le groupe HVTN (HIV Vaccine Trials Network) »

# 8. methodes statistiques et analyse des donnees

## 8.1. Détermination de la taille de l’échantillon

Dans cette première étude d’administration de lipopeptides spécifiques du VIH-1 par voie intradermique, l’objectif principal de l’essai est d’évaluer la tolérance de l’administration des lipopeptides spécifiques du VIH-1 par voie intradermique et la comparer à la voie intramusculaire. Les autres objectifs sont de comparer les 2 voies d’administration (intramusculaire et intradermique) en terme d’immunogénicité car on recherche une efficacité plus grande avec une dose plus faible de vaccin. L’étude est comparative, les sujets étant randomisés selon le ratio 1 :1 entre la voie d’administration intradermique et la voie intramusculaire.Le nombre de sujets à inclure est de 35 sujets par groupe, défini à partir des considérations suivantes :

- puisqu’il s’agit de la première étude évaluant l’injection par voie intradermique, les événements indésirables de grade 3 ou 4 seront recensés en temps réel et l’attitude suivante sera adoptée : la survenue d’un événement de grade 4 ou chez au moins 3 volontaires, d’événements locaux (au site d’injection) et/ou généraux de grade 3 entraînera l’arrêt de l’essai. Avec 35 sujets par groupe de vaccination, si l’on observe 2 événements indésirables de degré ≥ 3, la borne supérieure de l’intervalle de confiance à 95% de la proportion d’évènements de degré ≥ 3 est supérieure à 19% (selon la loi binomiale).
- la tolérance des 2 voies d’injection sera comparée sur l’ensemble des événements indésirables (EI) de grade supérieur ou égal à 2 pouvant être liés au produit. Les douleurs immédiates et de courte durée au point d’injection étant extrêmement fréquentes (douleurs de degré 2 survenant chez 12/15 volontaires de VAC12) sont exclues du critère principal. Dans l’essai VAC12, en voie IM, 7/15 (46%) volontaires ont présenté au moins 1 événement indésirable de grade sup. ou égal à 2 pouvant être lié au produit, douleurs exclues. Avec l’hypothèse que le pourcentage de survenue des EI de grade ≥2 dans la voie IM est de 45%, avec 35 sujets dans chaque groupe, on aura une puissance supérieure à 80% pour mettre en évidence un pourcentage de survenue d’EI de grade sup. ou égal à 2 de 80% dans la voie ID (risque de 1^ère^ espèce bilatéral à 5%). Une analyse en tenant compte des douleurs sera également réalisée.
- Pour l’étude de la réponse immunitaire, mesurée par la réponse proliférative des lymphocytes T helper aux antigènes du VIH , une taille d’échantillon de 35 sujets par groupe permettrait de mettre en évidence une différence entre les voies d’administration de 35% sur les pourcentages de répondeurs (seuil de signification alpha 0.05 (test de Fisher bilatéral), puissance 80%).
- Il est prévu d’évaluer la réponse des cellules CD8+ uniquement chez les volontaires dont le typage HLA laisse prévoir une réponse à plus de 4 peptides . Dans l’essai VAC12, 25% des volontaires (3/15) sont exclus pour cette raison de l’analyse des cellules CD8+. En ce qui concerne la capacité des CD8+ à sécréter l’INF-γ en présence de peptides et épitopes LPVIH1 par ELISPOT INF-γ, la proportion de répondeurs à au moins 1 peptide dans le groupe « voie intramusculaire » est d’environ 75% et la proportion de répondeurs à 1 peptide au moins deux fois, est de l’ordre de 40% (5/12). Avec 30 sujets évaluables par groupe, une différence de 35% en faveur de la voie intradermique sur ce dernier critère pourrait être mise en évidence (puissance proche de 80%, risque de 1^ère^ espèce bilatéral à 5%). Au cas où la différence d’efficacité ne serait pas significative par manque de puissance, on sera capable d’estimer l’écart entre les taux de réponse des 2 voies d’administration (différence entre les pourcentages de réponses assortie de son intervalle de confiance) et de préciser la borne inférieure de la différence d’efficacité en défaveur de la nouvelle voie d’administration.

## 8.2. Randomisation et stratification

L’étude est un essai randomisé dans lequel les volontaires seront répartis entre les deux voies d’administration de manière aléatoire. Le Service Commun 10 préparera les listes de randomisation et mettra en place la procédure de randomisation afin de préserver le secret de l'assignation dans les groupes vis-à-vis des cliniciens responsables de l'évaluation des volontaires à leur inclusion.

Etant donné que la voie d’administration différencie les groupes de traitement, l’insu sur la tolérance ne peut être assuré. En revanche, l’insu sur l’évaluation de la réponse immunitaire sera assuré.

Une stratification est prévue pour équilibrer l'attribution des vaccins dans les sous-groupes de volontaires présentant les mêmes caractéristiques HLA vis-à-vis du nombre de peptides LPVIH1 testés : les volontaires seront divisés en trois strates avant d’être randomisés (1 : HLA permettant de tester 0 à 1 peptide, 2 : 2 à 5 peptides ; 3 : 6 à 11 peptides).

8.3. Données à analyser

### 8.3.1. Populations incluses dans les analyses

Le principe de l'analyse en intention de traiter stipule que tous les sujets randomisés doivent être suivis jusqu'à la fin de l'essai indépendamment du fait que le sujet reçoive toutes les immunisations prévue dans l'étude. L’analyse de la population *per protocole* sera effectuée et comparée à l’analyse en intention de traiter.

### 8.3.2. Analyse du critère principal : tolérance

Cette analyse portera sur tous les sujets ayant reçu au moins une dose d’un candidat-vaccin à l’étude. A chaque temps d’évaluation, seuls seront pris en compte les sujets pour lesquels des données sont disponibles pour le paramètre considéré (les dénominateurs sont donc susceptibles de varier selon le temps d’évaluation et le paramètre considéré).

### 8.3.3. Analyse des critères secondaires : immunogénicité

L’analyse *per protocole* limitera l’étude aux volontaires ayant reçu le candidat-vaccin par la même voie d’administration tout au long de leur participation à l’essai. Tout sujet ayant reçu, à au moins une reprise, le vaccin par une voie autre que celle de la semaine S0 sera exclu de l'analyse *per protocole* de l’immunogénicité. Par contre, un sujet ayant reçu à chaque injection le candidat-vaccin par la même voie d’administration sera analysé dans le groupe correspondant au vaccin reçu, même si ce groupe diffère de celui prévu par la randomisation. Les données seront analysées selon la voie d’administration du vaccin effectivement utilisée à chaque temps.

## 8.4. Méthodologie statistique

### 8.4.1. Tolérance

#### 8.4.1.1. Tolérance locale

Le nombre et le pourcentage de sujets présentant au moins une réaction locale au cours de l’étude seront déterminés pour chaque groupe de randomisation globalement, puis en fonction du délai d’apparition (immédiat : <30 minutes, précoce [1-24h post injection], semi-retardé [24-72h post injection], retardé [72h-7jours] et ≥ 7 jours) et de l’intensité.

Pour chaque type de réaction, le nombre et le pourcentage de sujets concernés seront également présentés pour chaque groupe de randomisation globalement et en fonction de l’intensité maximale, du délai d’apparition et du nombre d’injections pour chaque volontaire.

#### 8.4.1.2. Tolérance générale

Le nombre et le pourcentage de sujets présentant au moins un événement indésirable systémique au cours de l’étude sera déterminé pour chaque groupe de randomisation globalement, puis en fonction de l’intensité maximale, de l’imputabilité au traitement et du délai d’apparition.

Pour chaque type d’événement, le nombre et le pourcentage de sujets concernés seront également présentés pour chaque groupe de randomisation globalement et en fonction de l’intensité maximale, de l’imputabilité, du délai d’apparition et du nombre d’injections pour chaque volontaire.

#### 8.4.1.3. Signes vitaux

Pour chaque paramètre mesuré (pressions artérielles systolique et diastolique, fréquence cardiaque, température buccale), la valeur moyenne, l’écart-type, les valeurs maximale et minimale seront donnés pour chaque groupe de randomisation à chacune des visites de vaccination et de suivi. Pour la température buccale, la fréquence des fièvres (température ≥ 37.5°C) sera tabulée sur les 5 jours suivant chaque injection, globalement et selon le délai d’apparition (immédiat : <30 minutes, précoce [1-24h post injection], semi-retardé [24-72h post injection], retardé [72h-3jours]) pour chaque voie d’administration.

#### 8.4.1.4. Tolérance biologique

Pour chaque paramètre biologique mesuré, la valeur moyenne, l’écart-type, les valeurs maximale et minimale seront donnés pour chaque voie d’administration à chacun des temps d’évaluation.

Des tables de “ transition ” présentant la fréquence des anomalies biologiques (valeurs supérieures ou inférieures aux normes de références du laboratoire réalisant les dosages) apparues au cours de l’étude (par comparaison aux valeurs mesurées à S0) seront construites pour chaque paramètre, à chaque temps d’évaluation.

Pour les tolérances locales, générales et biologiques, les voies d’administration seront caractérisées par les taux de survenue d’évènements indésirables accompagnés d’intervalles de confiance à 95% (distribution binomiale exacte) et comparées 2 à 2 par un test de Fisher exact (tests bilatéraux au seuil 5%).

### 8.4.2. Immunogénicité

Les résultats des paramètres évaluant la réponse immunitaire aux candidats-vaccins peuvent être exprimés selon trois grandes catégories (résultats dichotomiques, en positif/négatif ; résultats semi-quantitatifs, en plusieurs classes ordonnées ; et résultats quantitatifs), pour lesquelles l’abord statistique retenu sera différent.

#### 8.4.2.1. Abord qualitatif

Pour chacun des paramètres pour lesquels un seuil de positivité est défini, le nombre et le pourcentage de sujets positifs à chaque temps d’évaluation seront présentés pour chaque groupe. Les pourcentages de sujets positifs seront accompagnés de leurs intervalles de confiance à 95%, calculés en utilisant la distribution binomiale exacte.

#### 8.4.2.2. Abord semi-quantitatif

Pour chacune des classes définies, l’effectif de la classe et le pourcentage correspondant seront tabulés.

#### 8.4.2.3. Abord quantitatif

Pour chacun des paramètres pour lesquels une valeur quantitative (index de stimulation, nombre de coups par minute, nombre de spots etc.) est recueillie, les statistiques descriptives suivantes seront calculées pour chaque groupe à chaque temps d’évaluation : valeur minimale, valeur maximale, médiane. A titre indicatif et quand cela sera approprié, on calculera également la moyenne géométrique et son intervalle de confiance à 95% (par transformation logarithmique des données, calcul de la moyenne arithmétique et de son intervalle de confiance et transformation anti-log de cette moyenne et des bornes de l’intervalle de confiance).

Les deux groupes vaccinés seront comparés en ce qui concerne les taux de réponse à 1 peptide au moins deux fois par un test de Fisher exact (test bilatéral au seuil 5%).

Les analyses comporteront principalement des comparaisons à temps fixes S2, S6, S14 et sur l’ensemble des réponses au cours du temps (en particulier : réponses cumulées, au moins une réponse, réponses à 2 peptides au moins 2 fois), ajustées ou non sur la réponse à S0. La durabilité de la réponse sera évaluée par une analyse à S24.

# 9. comite de surveillance

## 9.1. Comité scientifique

Le conseil scientifique sera composé des personnes suivantes : D. Salmon, B. Silbermann, C. Desaint, J-P. Levy, C. Durier, J-G. Guillet, H. Gahery, I. Bourgault, V. Rieux, O. Launay, N. Guérin, C. Guérin, B. Rouveix, D. Hill, H. Gras, F. Heshmati, l’investigateur principal de chaque centre et un représentant de la CRO

## 9.2. Comité indépendant

Madame HERZLICH (présidente de ce comité indépendant)

Pr. Claude BAZIN (maladies Infectieuses et Réanimation Médicale, CHU de Caen)

Dr. Fabrice CARRAT (Inserm U444, Faculté de Médecine St Antoine, Paris)

Pr. Eric OKSENHENDLER (Service d’Immuno-hématologie, Hôpital St Louis, Paris)

Pr. Bruno VARET (Service d’Hématologie Adultes, Hôpital Necker, Paris)

# 10. ethique et aspect reglementaire

## 10.1. Aspects éthiques et réglementaires

Cet essai sera réalisé conformément au protocole et :

- au texte de la déclaration d'Helsinki adopté par l'Assemblée Médicale Mondiale en juin 1964, modifié à Tokyo (octobre 1975), Venise (octobre 1983), Hongkong (septembre 1989), à Somerwest (Afrique du Sud, octobre 1996) et à Edimburg (octobre 2000);
- aux recommandations des Bonnes Pratiques Cliniques (ICH May 1996) ;
- au texte de la loi française du 20 décembre 1988 n°88 - 11.38 sur la protection des personnes se prêtant à des recherches biomédicales (loi HURIET modifiée);
- à l'article L365 - 1 du code de la santé publique.

Le protocole a reçu l’avis favorable du CCPPRB de la Pitié-Salpétrière le 18/ 06/2003.

La version définitive sera approuvée par le Promoteur, l’investigateur coordonnateur et le pharmacologue participant à l’essai.

## 10.2. Amendement au protocole

Toute modification substantielle du protocole fera l'objet d'un amendement écrit qui sera soumis au Conseil Scientifique, au promoteur, puis au CCPPRB et signé par le Promoteur, l’investigateur coordonnateur et le pharmacologue. Une copie de l’amendement ainsi que de l’avis du CCPPRB correspondant sera envoyée à chaque centre.

## 10.3. Confidentialité des données

Les données des volontaires recueillies dans le cadre de cette étude resteront strictement confidentielles et anonymes. Seuls les investigateurs et les ARCs impliqués dans le déroulement de l’essai, ainsi qu’un représentant des Autorités de Santé et toute personne mandatée par le Promoteur, pourront avoir accès aux dossiers médicaux des volontaires. Ils le feront dans le seul but de vérifier l’exactitude des données collectées.

Les données enregistrées lors de cette étude feront l’objet d’un traitement informatisé pour le compte du Promoteur ; une déclaration de ce fichier sera faite à la CNIL par le Promoteur et la CRO conformément à la loi n°78-17 du 6 janvier 1978, relative à l’informatique, aux fichiers et aux libertés modifiée par la loi du 6 janvier 1978, Art.40, modifiée le 1^er^ juillet 1994.

Les données concernant un volontaire inclus et nécessaires à l’essai sont reportées dans le cahier d’observations du volontaire par l’investigateur, après chaque visite prévue dans le protocole. Seuls sont notés sur le cahier d’observation le numéro et le code d’identification du volontaire.

## 10.4. Assurance

“L’Agence Nationale de Recherche sur le Sida (ANRS), Promoteur de cet essai a souscrit un contrat d’assurance en responsabilité civile auprès de SM BIOMEDIC-Insure pour Axa Courtage conformément aux dispositions de l’article L1121-7 du Code la Santé Publique” (Annexe 8).

# 11. confidentialite et indemnisation

## 11.1. Confidentialité

### 11.1.1. Confidentialité des données

Les investigateurs s'engagent à tenir confidentielle toute information concernant le produit à tester, les individus prenant part à l'essai, le protocole et les résultats qui ne sont pas dans le domaine public. Les investigateurs devront s'assurer que tous leurs collaborateurs respectent les mêmes règles.

### 11.1.2. Confidentialité des dossiers des volontaires

En signant ce protocole, l'investigateur accepte que, dans le cadre des obligations légales imposées par la réglementation *nationale* en vigueur et dans le respect de l'éthique, le promoteur ou toute autre autorité légale puisse consulter et/ou copier les documents relatifs à l'étude afin de vérifier les données des cahiers d'observations.

## 11.2. Indemnisation des sujets

Les volontaires seront remboursés des frais relatifs aux visites spécifiques à l'essai. Ils ne recevront cependant pas d’indemnisation pour leur participation à cette étude.

# 12. REGLES DE PUBLICATION ET DE CESSION DES DONNEES

Les résultats de cette étude ne pourront faire l'objet de publications ou de communications qu'après accord de l'investigateur coordonnateur, du Conseil Scientifique de l’essai, et du promoteur (ANRS).

Un comité de rédaction des résultats sera désigné par l’investigateur coordonnateur.

Les conditions de cession de tout ou partie de la base de données de l’essai seront précisées par le Conseil Scientifique de l’essai en accord avec les contrats établis avec les partenaires de la recherche par l’ANRS et après accord du promoteur.

# 13. references bibliographiques

(1). AIDS epidemic update December 2003. Joint UNAIDS/WHO, December 2003.

(2). Levine AM et al. Initial studies on active immunization of VIH-infected subjects using a gp120-depleted HIV-1 immunogen: long term follow-up. J. Acquir. Immune Defic. Syndr. 1996, 11 :351-64.

(3). Mascola JR, et al. Protection of macaques against vaginal transmission of a pathogenic HIV-1/SIV chimeric virus by passive infusion of neutralizing antibodies. Nat. Med. 2000;6:207-210.

(4). Baba TW, et al. Human neutralizing monoclonal antibodies of the IgG1 subtype protect against mucasal simian-human immunodeficiency virus infection. Nat. Med. 2000;6:200-206

(5). Rosenberg ES et al. Vigorous HIV-1-specific CD4+T cell responses associated with control of viremia. Science 1997, 278 :1447-50

(6). Amara R. R., et al. Control of a mucosal challenge and prevention of AIDS by a multiprotein DNA/MVA vaccine. Science, 2001, 292:69-74.

(7). Barouch D.H., et al. Control of viremia and prevention of clinical AIDS in rhesus monkey by cytokine-augmented DNA vaccination. Science, 2000, 290:486-892.

(8). Vitiello A, et al. Development of a lipopeptide-based therapeutic vaccine to treat chronic HBV infection. J Clin Invest, 1995, 95:341-9.

(9). Livingston B.D., et al. The hepatitis B virus-specific CTL responses induced in humans by lipopoptide vaccination are comparable to those elicited by acute viral infection. J. Immunol, 1997, 159:1383-92.

(10). Sellati T.J., et al. The cutaneous response in humans to *Treponema pallidum* lipoprotein analogues involves cellular elements of both innate and adaptative immunity. J Immunol, 2001, 166:4131-40.

(11) Pialoux G., Gahery-Segard H., Sermet S., Poncelet H., Fournier S., Gerard L., Tartar A., Gras-Masse H., Levy J.P., Guillet JG. ANRS VAC 04 Study Team. Lipopeptides induce cell-mediated anti-HIV immune responses in seronegative volunteers. AIDS. 2001;15-1239.

(12) Levy Y, Gahery-Segard H, Durier C et al. Immunological and virological efficacy of ALVAC-VIH 1433 and HIV lipopeptides (lipo-6T) combined with SC IL62 in chronically HIV-infected patients- results of the ANRS 093 randomized study. Abstract n°62, 10^th^ Conference on Retroviruses and Opportunistic Infections. February 10-14, 2003. Boston, MA, USA.

(13) Shiver JW, Fu TM, Chen L et al. Replication incompetent adenoviral vaccine vector elicite effective anti-immunodeficiency-virus immunity. Nature. 2002 ; 415, 331-335.

(14). Gahery-Ségard H., et al. Multiepitopic B- and T-Cell Responses Induced in Humans by Human Immunodeficiency Virus Type 1 Lipopeptide Vaccine. J Virol. 2000;744-1694.

(15). Pialoux G., et al. Lipopeptides induce cell-mediated anti-HIV immune responses in seronegative volunteers. AIDS. 2001;15-1239.

(16) Gahery-Ségard H., Pialoux G, Figueiredo S, Igéa C, Surenaud M, Gaston Jessintha, Gras-Masse H, Levy JP, Guillet JG. Long term specific immune responses induced in humans by HIV-1 lipopeptide vaccine : characterization of CD8+-T-cell epitopes recogn ized. J. Virol, 2003, 77, 1-12.

(17). Gahery-Ségard H., et al. The multispecificB- and T-Cell responses indueced in humans by an HIV-1 lipopeptide vaccine can be sustained over a period of up to two years. Abstract n°45, AIDS Vaccine 2001. September 5-8, 2001. Philadelphia, PA, USA.

(18). Watson J.C., Peter G. General immunization practices. In: Plotkin S.A., Orenstein W.A., editors. Vaccines W.B. Third edition. W.B. Saunders Compagny, 1999, p. 47-73.

(19). Yokoyama A., et al. DNA immunization can stimulate florid local inflammation, and the antiviral immunity induced varies depending on injection site. Vaccine 1997;15(5):553-60.

(20). Raz E., et al. Intradermal gene immunization ; the possible role of DNA uptake in the induction of cellular immunity to viruses. Proc. Nat. Acad. Sci. USA 1994;91(20):9519-23.

(21). Puri N., et al. An investigation of the intradermal route as an effective means of immunization for microparticule vaccine delivery systems. Vaccine 2000;18:2600-12.

(22). Hunsaker B.D., et al. Efficacy of intradermal vaccination. Vet. Immunol. Immunopathol. 2001;79:1-13.

(23). Briggs D.J., et al. Antibody response of patients after postexposure rabies vaccination with small intradermal doses of purified chick embryo cell vaccine or purified Vero cell rabies vaccine. Bulletin if the World Health Organization, 2000;785(5):693-8.

(24). Gherardin A.W., et al. Antibody response to intramuscular booster in previously intradermally immunized travelers using human diploid cell rabies vaccine. J. Travel Med. 2001;8:122-8.

(25). Kurugol Z., et al. Low-dose intradermal administration of recombinant hepatitis B vaccine in children: 5-year follow-up study. Vaccine 2001;19:3936-9.

(26). Miller K.D., et al. Intradermal hepatitis B virus vaccine : immunogenicity and side effects in adults. Lancet 1983, 1454-6.

(27). King J.W., et al. Comparaison of the immunogenicity of hepatitis B vaccine administered intradermally and intramusculary. Rev. Inf. Dis. 1990;12:1035-43.

(28). Ren S, et al. Low-volume jet injection for intradermal immunization in rabbits. BMC Biotechnology. 2002, 2:10.

# 14. annexes

Annexe 1 : Déclaration d’Helsinki

Annexe 2 : Information du sujet et formulaire de consentement (pour l’ensemble des sujets pré-inclus)

Annexe 3 : Information du sujet et formulaire de consentement (pour les sujets acceptant de participer à la sous-étude)

Annexe 4 : Cotation des événements indésirables (échelle ANRS)

Annexe 5 : Fiche de déclaration d’événement indésirable

Annexe 6 : Fiche standard de recueil des données initiales/finales concernant la grossesse

Annexe 7 : Centres et personnel de l’essai

Annexe 8 : Attestation d’assurance et autorisations de lieux

Annexe 9 : Fiche de déclaration des événements locaux ou généraux de grade 3 pouvant être lié au produit.

Annexe 1

Déclaration d’Helsinki

Déclaration d'HELSINKI

de l'Association Médicale Mondiale

Principes éthiques applicables aux recherches médicales sur des sujets humains; adoptée par la 18e Assemblée générale, Helsinki, Juin 1964 et amendée par les 29e Assemblée générale, Tokyo, Octobre 1975 35e Assemblée générale, Venise, Octobre 1983 41e Assemblée générale, Hong Kong, Septembre 1989 48e Assemblée générale, Somerset West (Afrique du Sud), Octobre 1996 et la 52e Assemblée générale, Edimbourg, Octobre 2000

A. INTRODUCTION

1 La Déclaration d'Helsinki, élaborée par l'Association médicale mondiale, constitue une déclaration de principes éthiques dont l'objectif est de fournir des recommandations aux médecins et autres participants à la recherche médicale sur des êtres humains. Celle-ci comprend également les études réalisées sur des données à caractère personnel ou des échantillons biologiques non anonymes.

2. La mission du médecin est de promouvoir et de préserver la santé de l'être humain. Il exerce ce devoir dans la plénitude de son savoir et de sa conscience.

3. Le Serment de Genève de l'Association médicale mondiale lie le médecin dans les termes suivants : "La santé de mon patient sera mon premier souci" et le Code international d'éthique médicale énonce que "le médecin devra agir uniquement dans l'intérêt de son patient lorsqu'il lui procure des soins qui peuvent avoir pour conséquence un affaiblissement de sa condition physique ou mentale".

4. Les progrès de la médecine sont fondés sur des recherches qui, in fine, peuvent imposer de recourir à l'expérimentation humaine.

5. Dans la recherche médicale sur les sujets humains, les intérêts de la science et de la société ne doivent jamais prévaloir sur le bien-être du sujet.

6. L'objectif essentiel de la recherche médicale sur des sujets humains doit être l'amélioration des méthodes diagnostiques, thérapeutiques et de prévention, ainsi que la compréhension des causes et des mécanismes des maladies. Les méthodes diagnostiques, thérapeutiques et de prévention, même les plus éprouvées, doivent constamment être remises en question par des recherches portant sur leur efficacité, leur efficience et leur accessibilité.

7. Dans la recherche médicale comme dans la pratique médicale courante, la mise en œuvre de la plupart des méthodes diagnostiques, thérapeutiques et de prévention expose à des risques et à des contraintes.

8. La recherche médicale est soumise à des normes éthiques qui visent à garantir le respect de tous les êtres humains et la protection de leur santé et de leurs droits. Certaines catégories de sujets sont plus vulnérables que d'autres et appellent une protection adaptée. Les besoins spécifiques des sujets défavorisés au plan économique comme au plan médical doivent être identifiés. Une attention particulière doit être portée aux personnes qui ne sont pas en mesure de donner ou de refuser elles-mêmes leur consentement, à celles qui sont susceptibles de donner leur consentement sous la contrainte, à celles qui ne bénéficieront pas personnellement de la recherche et à celles pour lesquelles la recherche est conduite au cours d'un traitement.

9. L'investigateur doit être attentif aux dispositions éthiques, légales et réglementaires applicables à la recherche sur les sujets humains dans son propre pays ainsi qu'aux règles internationales applicables. Aucune disposition nationale d'ordre éthique, légal et réglementaire ne doit conduire à affaiblir ou supprimer les mesures protectrices énoncées dans la présente déclaration.

B. PRINCIPES FONDAMENTAUX APPLICABLES A TOUTE FORME DE RECHERCHE MEDICALE

10. Dans la recherche médicale, le devoir du médecin est de protéger la vie, la santé, la dignité et l'intimité de la personne.

11. La recherche médicale sur des êtres humains doit se conformer aux principes scientifiques généralement reconnus. Elle doit se fonder sur une connaissance approfondie de la littérature scientifique et des autres sources pertinentes d'information ainsi que sur une expérimentation appropriée réalisée en laboratoire et, le cas échéant, sur l'animal.

12. Des précautions particulières doivent entourer les recherches pouvant porter atteinte à l'environnement et le bien-être des animaux utilisés au cours des recherches doit être préservé.

13. La conception et l'exécution de chaque phase de l'expérimentation sur des sujets humains doivent être clairement définies dans un protocole expérimental. Ce protocole doit être soumis pour examen, commentaires, avis et, le cas échéant, pour approbation, à un comité d'éthique mis en place à cet effet. Ce comité doit être indépendant du promoteur, de l'investigateur ou de toute autre forme d'influence indue. Il doit respecter les lois et règlements en vigueur dans le pays où s'effectuent les recherches. Il a le droit de suivre le déroulement des études en cours. L'investigateur a l'obligation de fournir au comité des informations sur le déroulement de l'étude portant en particulier sur la survenue d'événements indésirables d'une certaine gravité. L'investigateur doit également communiquer au comité, pour examen, les informations relatives au financement, aux promoteurs, à toute appartenance à une ou des institutions, aux éventuels conflits d'intérêt ainsi qu'aux moyens d'inciter des personnes à participer à une recherche.

14. Le protocole de la recherche doit contenir une déclaration sur les implications éthiques de cette recherche. Il doit préciser que les principes énoncés dans la présente déclaration sont respectés.

15. Les études sur l'être humain doivent être conduites par des personnes scientifiquement qualifiées et sous le contrôle d'un médecin compétent. La responsabilité à l'égard d'un sujet inclus dans une recherche doit toujours incomber à une personne médicalement qualifiée et non au sujet, même consentant.

16. Toute étude doit être précédée d'une évaluation soigneuse du rapport entre d'une part, les risques et les contraintes et d'autre part, les avantages prévisibles pour le sujet ou d'autres personnes. Cela n'empêche pas la participation à des recherches médicales de volontaires sains. Le plan de toutes les études doit être accessible.

17. Un médecin ne doit entreprendre une étude que s'il estime que les risques sont correctement évalués et qu'ils peuvent être contrôlés de manière satisfaisante. Il doit être mis un terme à la recherche si les risques se révèlent l'emporter sur les bénéfices escomptés ou si des preuves consistantes de résultats positifs et bénéfiques sont apportées.

18. Une étude ne peut être réalisée que si l'importance de l'objectif recherché prévaut sur les contraintes et les risques encourus par le sujet. C'est particulièrement le cas lorsqu'il s'agit d'un volontaire sain.

19. Une recherche médicale sur des êtres humains n'est légitime que si les populations au sein desquelles elle est menée ont des chances réelles de bénéficier des résultats obtenus.

20. Les sujets se prêtant à des recherches médicales doivent être des volontaires informés des modalités de leur participation au projet de recherche.

21. Le droit du sujet à la protection de son intégrité doit toujours être respecté. Toutes précautions doivent être prises pour respecter la vie privée du sujet, la confidentialité des données le concernant et limiter les répercussions de l'étude sur son équilibre physique et psychologique.

22. Lors de toute étude, la personne se prêtant à la recherche doit être informée de manière appropriée des objectifs, méthodes, financement, conflits d'intérêts éventuels, appartenance de l'investigateur à une ou des institutions, bénéfices attendus ainsi que des risques potentiels de l'étude et des contraintes qui pourraient en résulter pour elle. Le sujet doit être informé qu'il a la faculté de ne pas participer à l'étude et qu'il est libre de revenir à tout moment sur son consentement sans crainte de préjudice. Après s'être assuré de la bonne compréhension par le sujet de l'information donnée, le médecin doit obtenir son consentement libre et éclairé, de préférence par écrit. Lorsque le consentement ne peut être obtenu sous forme écrite, la procédure de recueil doit être formellement explicitée et reposer sur l'intervention de témoins.

23. Lorsqu'il sollicite le consentement éclairé d'une personne à un projet de recherche, l'investigateur doit être particulièrement prudent si le sujet se trouve vis-à-vis de lui dans une situation de dépendance ou est exposé à donner son consentement sous une forme de contrainte. Il est alors souhaitable que le consentement soit sollicité par un médecin bien informé de l'étude mais n'y prenant pas part et non concerné par la relation sujet-investigateur.

24. Lorsque le sujet pressenti est juridiquement incapable, physiquement ou mentalement hors d'état de donner son consentement ou lorsqu'il s'agit d'un sujet mineur, l'investigateur doit obtenir le consentement éclairé du représentant légal en conformité avec le droit en vigueur. Ces personnes ne peuvent être inclues dans une étude que si celle-ci est indispensable à l'amélioration de la santé de la population à laquelle elles appartiennent et ne peut être réalisée sur des personnes aptes à donner un consentement.

25. Lorsque le sujet, bien que juridiquement incapable (un mineur par exemple), est cependant en mesure d'exprimer son accord à la participation à l'étude, l'investigateur doit obtenir que cet accord accompagne celui du représentant légal.

26. La recherche sur des personnes dont il est impossible d'obtenir le consentement éclairé, même sous forme de procuration ou d'expression préalable d'un accord, ne doit être conduite que si l'état physique ou mental qui fait obstacle à l'obtention de ce consentement est une des caractéristiques requises des sujets à inclure dans l'étude. Les raisons spécifiques d'inclure des sujets dans une étude en dépit de leur incapacité à donner un consentement éclairé doivent être exposées dans le protocole qui sera soumis au comité pour examen et approbation. Le protocole doit également préciser que le consentement du sujet ou de son représentant légal à maintenir sa participation à l'étude doit être obtenu le plus rapidement possible.

27. Les auteurs et les éditeurs de publications scientifiques ont des obligations d'ordre éthique. Lors de la publication des résultats d'une étude, les investigateurs doivent veiller à l'exactitude des résultats. Les résultats négatifs aussi bien que les résultats positifs doivent être publiés ou rendus accessibles. Le financement, l'appartenance à une ou des institutions et les éventuels conflits d'intérêt doivent être exposés dans les publications. Le compte-rendu d'une étude non conforme aux principes énoncés dans cette déclaration ne doit pas être accepté pour publication.

C. PRINCIPES APPLICABLES A LA RECHERCHE MEDICALE CONDUITE AU COURS D'UN TRAITEMENT

28. Le médecin ne peut mener une recherche médicale au cours d'un traitement que dans la mesure où cette recherche est justifiée par un possible intérêt diagnostique, thérapeutique ou de prévention. Quand la recherche est associée à des soins médicaux, les patients se prêtant à la recherche doivent bénéficier de règles supplémentaires de protection.

29. Les avantages, les risques, les contraintes et l'efficacité d'une nouvelle méthode doivent être évalués par comparaison avec les meilleures méthodes diagnostiques, thérapeutiques ou de prévention en usage. Cela n'exclut ni le recours au placebo ni l'absence d'intervention dans les études pour lesquelles il n'existe pas de méthode diagnostique, thérapeutique ou de prévention éprouvée.

30. Tous les patients ayant participé à une étude doivent être assurés de bénéficier à son terme des moyens diagnostiques, thérapeutiques et de prévention dont l'étude aura montré la supériorité.

31. Le médecin doit donner au patient une information complète sur les aspects des soins qui sont liés à des dispositions particulières du protocole de recherche. Le refus d'un patient de participer à une étude ne devra en aucun cas porter atteinte aux relations que le médecin entretient avec ce patient.

32. Lorsqu'au cours d'un traitement, les méthodes établies de prévention, de diagnostic ou de thérapeutique s'avèrent inexistantes ou insuffisamment efficaces, le médecin, avec le consentement éclairé du patient, doit pouvoir recourir à des méthodes non éprouvées ou nouvelles s'il juge que celles-ci offrent un espoir de sauver la vie, de rétablir la santé ou de soulager les souffrances du malade. Ces mesures doivent, dans toute la mesure du possible, faire l'objet d'une recherche destinée à évaluer leur sécurité et leur efficacité. Toute nouvelle information sera consignée et, le cas échéant, publiée. Les autres recommandations appropriées énoncées dans la présente déclaration s'appliquent. 17/10/00

Annexe 2

Information du sujet et formulaire de consentement

(pour l’ensemble des sujets pré-inclus)

Essai vaccinal de phase I-B comparant la tolérance et l'immunogénicité de deux voies d’administration (intramusculaire et intradermique) de lipopeptides (LPVIH1) spécifiques du virus VIH-1 chez des volontaires

**non infectés par le VIH.**

***Essai sans bénéfice individuel direct***

**FORMULAIRE D’INFORMATION ET CONSENTEMENT ECLAIRE DU SUJET**

*Merci de lire attentivement ce document d’information,*

*il vous servira de base pour nos discussions ultérieures.*

Madame, Monsieur,

En tant que volontaire appartenant au réseau « Volontaires pour un vaccin » de l’ANRS (Agence Nationale de Recherche sur le SIDA), vous êtes sollicité pour participer à un essai clinique dont le promoteur est l’ANRS. Ce document fournit des informations plus précises sur le but de cet essai, la préparation vaccinale qui est utilisée dans cette étude, le déroulement même de l'étude, les risques potentiels ainsi que vos droits en tant que volontaire à cette étude. Nous vous demandons donc de lire attentivement ce document dans sa totalité et de ne pas hésiter à poser toutes les questions au médecin qui vous présente ce document.

**Pourquoi cette étude ?**

Le syndrome d'immunodéficience acquise ou SIDA est la forme la plus avancée de l'infection par le virus de l'immunodéficience humaine ou VIH, virus dont il existe actuellement deux types: le VIH 1 et le VIH 2, le premier étant le plus répandu dans le monde. Devant l'importance de l'épidémie, l'insuffisance des modes de prévention préconisés et l’absence de traitement permettant de guérir la maladie, la mise au point d'un vaccin efficace contre l'infection par le virus VIH est un problème de Santé Publique prioritaire.

Actuellement, différents types de candidats-vaccins sont à l’étude tels que les vaccins recombinants viraux, les vaccins ADN et les lipopeptides. La préparation vaccinale utilisée dans cet essai est constituée de lipopeptides du VIH. Ces produits sont fabriqués par synthèse chimique. Ce type de préparation vaccinale a été testé en France chez plus de 100 volontaires sains, totalisant plus de 400 injections. Par voie intramusculaire, ces injections sont très bien tolérées localement au point d’injection et au niveau de l’organisme en général.

La préparation de lipopeptides qui est utilisée dans cet essai, LPVIH1, est constituée d'un mélange de 4 lipopeptides du VIH. Chaque lipopeptide du produit est constitué d’un peptide (petit morceau de protéine) sélectionné du VIH qui est associé à un peptide issu de la toxine tétanique et à un lipide dans le but d'augmenter la réponse immunitaire. Ce produit a récemment été utilisé dans l’essai VAC12 afin d’évaluer la tolérance et les réponses immunitaires observées après administration par voie intramusculaire. Dans cette étude, les injections étaient bien tolérées en dehors de douleurs au point d’injection (le plus souvent modérées) survenant pendant et dans les heures suivant l'injection. Les premiers résultats concernant les réponses immunitaires cellulaires T, impliquées dans la défense de l’organisme, sont encourageants.

Jusqu’à présent, au cours des précédents essais chez des volontaires non infectés, les lipopeptides ont été administrés par voie intramusculaire. Cependant les vaccins peuvent être administrés par d’autres voies notamment la voie intradermique. L’administration par voie intradermique se fait dans la peau. Cette voie d’administration est utilisée pour d’autres vaccins et permet dans certains cas (comme la vaccination contre la rage, l’hépatite ou le BCG) une meilleure efficacité que la voie intramusculaire. Elle permettrait d’utiliser de plus faibles doses de vaccin (environ 5 à 10 fois plus faibles) et de diminuer ainsi les coûts de la vaccination. C’est pourquoi dans cette étude, nous souhaitons comparer la tolérance et la réponse immunitaire de ces lipopeptides anti-VIH selon que l’administration se fait par voie intramusculaire ou intradermique.

**Buts de cette étude ?**

Cette étude a pour but principal de comparer la tolérance clinique (locale et générale) et biologique d’une préparation vaccinale de lipopeptides du VIH, appelée LPVIH-1, par voie intradermique comparée à une administration intramusculaire .

Cette étude comparera également la réponse immunitaire induite par ce produit selon que ce l’administration se fait par voie intramusculaire ou intradermique.

Néanmoins, cet essai n'a pas pour but de tester une éventuelle propriété protectrice contre le VIH. Il ne vise qu'à évaluer la tolérance de cette préparation et la réaction immunologique qu’elle entraîne. Les connaissances ainsi acquises serviront à la préparation d'un futur vaccin.

**Comment va se dérouler cette étude ?**

Cette étude se déroulera sur une durée totale de 10 mois. Elle se déroulera dans 6 centres en France. Au total, 70 volontaires seront inclus.

Votre participation à l’étude sera de 32 semaines. Vous aurez en tout 8 visites et une injection de la préparation vaccinale aura lieu au cours de trois d’entre elles.

- La première visite constitue la visite de pré-inclusion et a lieu 6 semaines avant la première vaccination. Elle permet de vérifier que vous ne présentez pas de contre-indications cliniques ou biologiques à une participation à l’essai et comporte donc un examen clinique effectué par un médecin et un prélèvement sanguin en vue d’un bilan biologique et de l’étude immunologique.
- La détermination de votre groupe se fera par tirage au sort avant l’inclusion, date de la première injection.
- Vous effectuerez 3 visites de vaccination (aux semaines S0, S4 et S12) qui comporteront un examen clinique avant l’injection et 30 minutes après. L’injection de la préparation vaccinale se fera dans la région deltoïdienne pour la voie intramusculaire et dans la face externe du bras pour la voie intradermique. Selon le groupe auquel vous appartiendrez, la voie d’injection sera toujours la même au cours de l’essai. Après l’injection, un carnet d'auto-surveillance, une réglette et un thermomètre vous seront remis. Ils vous permettront de surveiller et de noter toutes les réactions locales et/ou événements survenus entre 2 visites et de les rapporter au médecin lors de la visite suivante. Des prélèvements sanguins et urinaires seront également effectués à chacune de ces trois visites de vaccination.

| Groupe | Voie d’administration | Dose de LPVIH1  (dose totale) | Sujets  (Total = 70) | Schéma d'immunisation en semaines | | | |
| --- | --- | --- | --- | --- | --- | --- | --- |
|  |  |  |  | 0 | 4 | 12 | 24§ |
| A | intramusculaire | 500µg  (2000µg) | 35 | LPVIH1 | LPVIH1 | LPVIH1 |  |
| B | intradermique | 100µg  (400µg) | 35 (dont 6 §) | LPVIH1 | LPVIH1 | LPVIH1 | LPVIH1§ |

§ : sujets participant à la sous-étude physiopathologique et ayant une biopsie cutanée à S24+48H

- Enfin, 4 visites de suivi sont prévues aux semaines S2, S6, S14 et S24 afin de recueillir les données concernant la tolérance au vaccin. Chacune de ces visites inclut un entretien et un examen clinique, une discussion sur les événements ayant eu lieu depuis la visite précédente, une prise de sang et un examen d'urine.
- Une dernière visite, 12 jours après la visite de la 24^ème^ semaine, est prévue afin de vous communiquer les résultats des analyses biologiques réalisées à S24 et en particulier le résultat de la sérologie VIH.

| **Date (mois et jours)** | **S-6** | **S0** | **S2** | **S4** | **S6** | **S12** | **S14** | **S24** | **S24+12J** |
| --- | --- | --- | --- | --- | --- | --- | --- | --- | --- |
| **Intervalle (jours)** |  |  |  |  |  |  |  |  |  |
| Examen clinique | ✓ | ✓ | ✓ | ✓ | ✓ | ✓ | ✓ | ✓ | ✓ |
| Prélèvement sanguin | ✓ | ✓ | ✓ | ✓ | ✓ | ✓ | ✓ | ✓ |  |
| Prélèvement urinaire | ✓ | ✓ | ✓ | ✓ | ✓ | ✓ | ✓ | ✓ |  |
| **vaccination** |  | **1** |  | **2** |  | **3** |  |  |  |

Au total, des volumes de sang de 1026 ml pour les hommes et de 1036ml pour les femmes seront prélevés sur les 32 semaines de l’essai. Le volume total de sang prélevé n'excède pas la quantité légale que vous êtes autorisé à donner sur cette période, comme c'est le cas pour les donneurs de sang réguliers.

Cependant, le volume de sang prélevé au cours de l’étude peut diminuer vos réserves en fer qui sont normalement rapidement reconstituées par l’alimentation. C’est pourquoi, lors de la pré-inclusion, une mesure de la ferritinémie (taux de fer dans le sang) sera effectuée. Il se peut que ce taux soit alors inférieur à la normale. Nous vous proposerons alors une supplémentation en fer pendant 3 mois puis nous vous proposerons une nouvelle visite de pré-inclusion au cours de laquelle nous réaliserons un nouveau prélèvement de sang de 18 ml pour les hommes et de 23 ml pour les femmes. Ce prélèvement permettra de vérifier votre bilan hématologique et biochimique dont la ferritine. Un suivi de la ferritinémie sera effectué tout au long de l’étude et une supplémentation en fer pourra à nouveau vous être proposée si besoin. Cette supplémentation peut dans certains cas, entraîner des désagréments digestifs (diarrhée, douleurs abdominales).

Une partie du sang prélevé sera congelée et conservée dans la biothèque (collection d’échantillons sanguins congelés) de l’étude. Ces échantillons congelés seront utilisés pour vérifier éventuellement certaines données, réaliser des recherches biologiques particulières dans la mesure où celles-ci permettraient de mieux comprendre la tolérance et l’immunogénicité du candidat-vaccin. Si d’autres études notamment génétiques devaient être réalisées, un nouveau consentement vous sera demandé.

Pour les sujets recevant le vaccin par voie intradermique, il leur sera proposé, lors de la visite de la douzième semaine (S12) et en fonction des résultats des tests biologiques, de participer à une sous-étude physiopathologique. Cette étude aura pour but d’étudier la réponse immunitaire locale au niveau du site d’injection. Cette sous-étude consistera en une quatrième injection intradermique, réalisée à la 24^ème^ semaine à la face interne du bras, suivie 48H après, par une biopsie cutanée au point d’injection. Cette biopsie sera réalisée, sous anesthésie locale, et nécessitera un point de suture qui peut être responsable d’une cicatrice. Cette sous-étude fera l’objet d’un nouveau consentement et votre participation n’est pas obligatoire.

**Quels sont les risques potentiels de la préparation vaccinale et des injections?**

♦ **Cette préparation vaccinale est issue de la synthèse chimique et ne contient aucune particule virale. Il n'y a donc aucun risque d'infection ni de transmission du VIH, ni a fortiori de risque de SIDA**

Les lipopeptides utilisés lors des précédents essais vaccinaux, étaient responsables chez certains volontaires de la production d’anticorps anti-VIH, qui entraînent un résultat positif au test de dépistage ELISA ou « pseudo-séropositivité ». Cependant, dans ce cas, un test de confirmation utilisé habituellement, le WESTERN BLOT, est capable, de démontrer que cette séropositivité au test ELISA est liée à l’injection de la préparation vaccinale et non, à une contamination par le VIH.

***Cependant, avec les lipopeptides qui sont utilisés dans cet essai, il est peu probable que vous développiez ces anticorps responsables de la pseudo-séropositivité. Dans un essai précédent, utilisant la même préparation vaccinale que celle qui est utilisée dans cet essai, aucun sujet n’a développé cette « pseudo-séropositivité ».***

Néanmoins, il vous sera remis une carte plastifiée bilingue en français et en anglais portant vos nom(, prénom, photographie, numéro d'identification de l'essai, ainsi que les mentions « Réseau Volontaire ANRS » et « La personne porteuse de cette carte participe à un essai vaccinal contre le VIH et a été immunisée par un lipopeptide pouvant positiver les tests ELISA ». Il vous est recommandé de porter cette carte en permanence sur vous, à proximité de vos papiers d'identité. De plus, en cas d'urgence médicale, un numéro de téléphone pourra être utilisé 24 h sur 24 pour confirmer votre participation à cet essai vaccinal. Sur demande écrite auprès des investigateurs, il sera possible de fournir à toute personne ou institution que vous jugez nécessaire d'informer, une attestation de participation au présent essai.

♦ D’autre part, il existe aussi un risque théorique que cette préparation vaccinale puisse accroître la sensibilité des cellules à l’infection par le virus du Sida en cas d’exposition ce qui constitue une raison supplémentaire pour se protéger du risque de contamination par le VIH.

**Par conséquent, il est impératif que vous vous protégiez vis à vis de tout risque de contamination par le VIH, notamment par voie sexuelle. Vous ne serez en effet pas protégé contre le VIH par le candidat-vaccin.**

♦ Dans l'état actuel du développement de la recherche, d’autres risques théoriques persistent:

1) ces préparations vaccinales pourraient altérer le système immunitaire et provoquer par exemple des réactions auto-immunes. Ce risque théorique peut être évoqué avant l'injection de toute nouvelle protéine dans l'organisme et n’est pas propre aux préparations vaccinales

2) en recevant cette préparation vaccinale, on ne peut pas exclure que la réponse de votre organisme puisse être modifiée et rendent moins efficace dans l’avenir une vaccination avec un vaccin anti-VIH efficace.

♦ Les effets du LPVIH1 sont inconnus sur la grossesse ou l'enfant à naître. De ce fait, toute femme participant à l'essai et susceptible d'avoir un enfant devra s'assurer d'une contraception efficace. Si vous allaitez, vous ne pourrez pas participer à l'étude. Un test de grossesse sera réalisé lors du bilan biologique à l'inclusion dans l'essai, ainsi qu'à plusieurs reprises au cours de l'essai.

♦ Les injections seront faites par voie intramusculaire ou intradermique selon le groupe qui vous sera attribué. Comme toute injection, il existe des risques locaux qui peuvent se résumer à une réaction au lieu d'injection (douleur, contracture, rougeur, gonflement, induration, moins de 10% des volontaires ont présenté une forte gêne au point d'injection ayant duré quelques minutes) ainsi que quelques signes généraux (fièvre, frissons, fatigue, maux de tête ou éruption). Ces effets secondaires, bien connus des médecins, sont habituellement de courte durée et ne nécessitent généralement pas de traitement médical spécifique. Vous pourrez également ressentir les désagréments mineurs liés aux prises de sang. Les médicaments à prendre en cas de douleurs vous seront prescrits au moment des injections.

♦ Lors d’un essai déjà réalisé et utilisant le même candidat-vaccin, certains volontaires ont présenté des arthralgies (douleurs articulaires) localisées au niveau des épaules, des genoux, des hanches ou de l’articulation de la mâchoire. Ces douleurs étaient le plus souvent de courte durée (moins de 24h) sauf dans 2 cas où elles ont persisté pendant 5 jours.

♦ Une information complémentaire doit être portée à votre connaissance concernant la survenue de 2 cas d'uvéites survenus après vaccinations par des lipopeptides du VIH en association à d’autres candidats-vaccins.

L'uvéite est une inflammation interne de l'œil. C’est une maladie rare (environ 17 cas pour 100.000 personnes) et dans la moitié des cas, la cause de l’uvéite ne peut être retrouvée. Des épisodes d’uvéite ont été rapportés avec des vaccinations courantes, comme celles contre la grippe, l'hépatite B ou la poliomyélite (au total 20 cas ont été déclarés à ce jour à Aventis Pasteur). Deux volontaires, participant en France à un essai vaccinal contre le VIH, ont présenté une uvéite sans gravité, respectivement 4 mois et 1 an après administration de combinaisons vaccinales de lipopeptides associé à un adjuvant potentiellement responsable des complications ophtalmologiques. Ces événements ont régressé sans séquelle sous traitement local par collyres et leur relation avec l'administration de lipopeptides n'a pas été établie à ce jour. Aucune uvéite n’a été observée chez les volontaires ayant reçu les formulations à l’étude dans cet essai (LPVIH1). En cas de manifestation oculaire (douleur ou rougeur de l’œil, baisse de la vision) pouvant faire évoquer une uvéite nous vous demandons donc de prendre contact le plus rapidement possible avec l'un des médecins chargés de cet essai ou de consulter un ophtalmologiste.

**Ce qu'il vous est demandé dans cette étude?**

♦ Etant donné les risques potentiels évoqués ci–dessus, il vous est demandé de continuer de vous protéger du risque de contamination par le VIH. Les résultats obtenus chez l'animal, ainsi que les premiers résultats issus des essais cliniques français et américains montrent qu'il n'y a encore aucune preuve que la réponse immunitaire obtenue chez l'Homme puisse avoir de réelles propriétés protectrices contre l'infection par le virus du SIDA.

♦ En l’absence d’efficacité du candidat-vaccin contre l’infection par le VIH, cet essai est sans bénéfice direct pour vous, puisque aucun avantage médical résultant de votre participation ne peut vous être garanti.

**L'affiliation à un régime de la Sécurité Sociale est obligatoire pour participer à cette étude.**

Pendant la période de l'essai de 32 semaines et 4 mois après la fin de cet essai (période d’exclusion), vous ne pourrez participer à aucune autre recherche biomédicale. Il vous sera également demandé de ne pas donner votre sang ni de tissu humain (moelle, organes, ...) durant l’essai puis pendant la période d’exclusion et/ou négativation du test ELISA VIH.

♦ Vous êtes libre d'informer toute personne de votre participation à cet essai clinique, nous souhaitons cependant vous mettre en garde contre les tentations médiatiques liées à ce type d'essai. En effet, vos déclarations pourraient être mal interprétées et risquent, par exemple, de faire naître des espoirs prématurés sur un vaccin.

**Quels sont vos droits ?**

Vous êtes entièrement libre d'accepter ou de refuser de participer à cette étude, cela ne remettra pas en cause votre participation au réseau des volontaires de l’ANRS. Vous êtes entièrement libre d'arrêter votre participation à l'étude et ce, à tout moment. Dans ce cas, il vous sera demandé de prévenir l'un des médecins précédemment rencontrés.

Nous vous avertirons de toutes nouvelles informations pouvant vous amener à remettre en cause votre participation dans cet essai.

Pour tout renseignement, vous pouvez joindre, soit par l’intermédiaire du répondeur, soit directement, l’un des médecins chargés de cette étude.

A l’issue de la recherche, si vous le souhaitez, une note d’information sur les résultats globaux de l’essai vous sera fournie.

**Confidentialité**

Tous les documents vous concernant seront rendus anonymes, que ce soient les tubes de prélèvements ou les résultats biologiques. Toutes les données recueillies dans le cadre de cette étude resteront strictement confidentielles. Vos noms et adresse ne seront connus que de votre médecin. L'accès à votre dossier de surveillance est réservé à un nombre limité de personnes toutes tenues au strict respect du secret professionnel

Compte tenu de la nécessité de la recherche et de son analyse ultérieure, les données recueillies vous concernant feront l’objet d’un traitement informatisé et anonyme au niveau de la société prestataire chargée de cette fonction et mandatée par l’ANRS.

La loi « Informatique et Libertés » du 6 janvier 1978 (article 40) prévoit votre droit d’opposition à l’informatisation de ces données ainsi que votre droit d’accès à ces informations et de rectification de celles-ci pendant la durée de l’essai.

Les informations qui feront l’objet de ce traitement informatisé seront les suivantes : date de naissance, sexe, événements cliniques, résultats biologiques, immunologiques et virologiques, traitements reçus et en cours.

**Informations complémentaires**

*Recherche auprès de la mairie de naissance d'un sujet perdu de vue*

Il est d'usage dans un essai clinique d'apporter la preuve qu'une personne, incluse dans un essai et avec qui le contact a été perdu en cours d’essai, n'est pas décédée. Pour cela, le médecin responsable de l'essai écrit à la mairie de naissance de cette personne pour demander cette information.

Le promoteur de cet essai, l’ANRS, fera appel à ce type de recherche d'information si nécessaire.

*Aspects réglementaires :*

Le protocole de réalisation de cet essai a reçu l'approbation du Comité Consultatif de Protection des Personnes dans la Recherche Biomédicale de la Pitié-Salpétrière. L'essai se déroule sous la promotion de l'Agence Nationale de Recherche sur le SIDA (ANRS).

*Assurance responsabilité civile*: L’ANRS a souscrit un contrat de responsabilité civile conformément aux dispositions de l’article L.1121-7 du code de la santé publique du 20 décembre 1988, et de l’article 5 du 25 juillet 1994.

*Inscription au fichier national* : Cette étude étant sans bénéfice individuel direct, vous serez inscrit par Minitel sur le fichier national des volontaires, géré par le ministère de la Santé, sur lequel seront notées vos initiales, date de naissance, dates de début et de fin d’étude ainsi que la date de fin d’exclusion (qui est de 4 mois). Vous pourrez à tout moment vérifier l’exactitude de ces données auprès du titulaire de l’autorisation de lieu de recherche et leur destruction au terme du délai prévu à l’article R.2045 du code de la santé publique.

*Frais engagés****:***

Les frais de transport et d’hébergement engagés pendant la période de l'essai vous seront remboursés sur justificatifs.

**CONSENTEMENT ECLAIRE DU SUJET**

Je soussigné(e) (nom, prénom) .........................................................................................

N° de sécurité sociale :...............................................

Certifie avoir lu et compris l’information indiquée ci-dessus.

Le Dr...............................................m'a proposé de participer à un essai vaccinal avec le candidat-vaccin lipopeptides du VIH (LPVIH1) pour comparerla tolérance et l’immunogénicité de ce produit administré par voie intramusculaire ou intradermique, recherche organisée par l'ANRS.

J'ai eu la possibilité de poser toutes les questions relatives à l'étude, au médecin qui est chargé de mon suivi.

Je comprends les contraintes liées à ma participation.

Il m'a précisé que je suis libre d'accepter ou de refuser de participer à cette étude et que cette recherche n'entraînera aucun bénéfice individuel direct pour ma santé.

Je connais la possibilité qui m'est réservée d'interrompre cette étude à tout moment sans que cela me soit préjudiciable et sans avoir à justifier ma décision mais, je m'engage à en informer le Dr ...............................................

J'accepte que les données enregistrées à l'occasion de ce protocole puissent faire l'objet d'un traitement informatisé anonyme par le promoteur ou pour son compte. Cependant, des personnes mandatées par l’ANRS ou par les autorités de santé pourront avoir accès aux données qui me concernent, dans le respect le plus strict de la confidentialité, pour vérifier que l'étude s'est déroulée de manière conforme à la loi HURIET. Je note que le droit d'accès prévu par la loi "informatique et libertés" (Art. 40) s'exerce à tout moment et que je pourrai exercer mon droit de rectification et d'opposition auprès de l’ANRS par l'intermédiaire du Dr …………………

J'accepte d'être inscrit sur le fichier national des volontaires géré par le Ministère de la Santé et j’ai été informé que je peux vérifier l’exactitude des données qu’il contient et leur destruction ultérieure.

Pendant la durée prévue de ma participation, j’accepte qu’il soit procédé à une enquête auprès de ma mairie de naissance dans le cas où je cesserais de participer à cette recherche sans avertir l’un des médecins responsables de l’essai et sans répondre aux demandes de renseignements sur mon état de santé qui me seraient adressées.

Je m'engage à ne pas participer à une autre recherche biomédicale sans bénéfice individuel direct pendant la durée de la recherche. La durée d'exclusion pour la participation à une autre recherche sans bénéfice individuel direct est de 4 mois après la fin d'étude.

J'aurai connaissance des résultats des examens médicaux pratiqués durant l'essai.

Cette étude a reçu un avis favorable du Comité Consultatif de Protection des Personnes dans la Recherche Biomédicale de la Pitié-Salpétrière le 18 / 06 / 2003.

L’ANRS, promoteur de cette étude, a souscrit un contrat d'assurance de responsabilité civile qui me protège des risques encourus par l'essai, conformément à la loi HURIET (art. L1121-7 du Code de la Santé Publique).

J'accepte de participer à cette étude dans les conditions précisées ci-dessus.

Mon consentement ne décharge en rien les organisateurs de l'étude de leurs responsabilités, je conserve tous mes droits garantis par la loi HURIET du 20/12/88, modifiée le 24/07/94.

Fait à ........................................le...................................... Signature

Je soussigné (e), Docteur....................................., certifie avoir communiqué toute information utile concernant cette étude. Je m'engage à faire respecter les termes de cette note de consentement, conciliant le respect des droits et des libertés individuelles et les exigences d'un travail scientifique.

Fait à ........................................le...................................... Signature

Fait en 3 exemplaires : 1 exemplaire pour le volontaire, 1 pour le médecin et 1 pour l’ANRS (ce dernier sera mis sous enveloppe scellée pour préserver la confidentialité)

Annexe 3

Information du sujet et formulaire de consentement

(pour les sujets participant à la sous-étude)

Essai vaccinal de phase I-B comparant la tolérance et l'immunogénicité de

**deux voies d’administration (intramusculaire et intradermique)**

**de lipopeptides (LPVIH1) spécifiques du virus VIH-1**

**chez des volontaires non infectés par le VIH.**

- **Sous-étude physiopathologique : réponse immunitaire locale –**

*essai sans bénéfice individuel direct*

FORMULAIRE D’INFORMATION ET CONSENTEMENT ECLAIRE DU SUJET

*Merci de lire attentivement ce document d’information,*

*il vous servira de base pour nos discussions ultérieures*

Madame, Monsieur,

Vous avez participé à l’essai VAC16 dont l’objectif était de comparer la tolérance et l’immunogénicité de lipopeptides LPVIH-1 administrés par voie intramusculaire ou intradermique. Dans cette étude, vous avez été tiré au sort dans le groupe correspondant à l’administration du produit par voie intradermique. C’est pourquoi, nous vous proposons de participer à la sous-étude physiopathologique sur la réponse immunitaire locale.

**Pourquoi cette sous-étude** **?**

Cette sous-étude a comme objectif de mieux comprendre les mécanismes de la réponse immunitaire, induite par les lipopeptides LPVIH1 au site d’injection, et plus particulièrement la réponse des lymphocytes T cytotoxiques. En effet, les cellules immunitaires sont bien représentées à la fois dans le derme et dans l’épiderme et l’étude immunologique des cellules présentes au niveau du site d’injection permettra de mieux comprendre la réponse au vaccin. Elle ne concernera donc que les volontaires tirés au sort dans le groupe d’administration intradermique qui présenteront un groupe HLA approprié (ensemble de molécules présentes à la surface de certaines cellules impliquées dans les phénomènes immunitaires de reconnaissance du soi ou du non-soi).

**En quoi consiste cette étude?**

Cette sous-étude durera 2 semaines, en plus des 30 semaines de l’essai, et comportera 2 visites supplémentaires à S24+2J et à S24+12J.

- Lors de la visite de la 24ème semaine, un examen clinique sera réalisé avant et 30 minutes après l’injection. Cette quatrième injection de LPVIH1 ( utilisant la même dose de produit que celle utilisée pour les injections précédentes) sera effectuée par voie intradermique, mais cette fois-ci à la face interne du bras. Cette quatrième injection vous sera proposée après s’être assuré que votre tolérance aux injections précédentes était satisfaisante. Après l’injection, un carnet d’auto-surveillance vous sera remis afin de noter toutes les réactions locales et/ou événements survenus entre la visite de l’injection et la visite suivante. Des prélèvements sanguin et urinaire seront effectués notamment le dosage du complément.
- A la visite à S24+J2, qui aura lieu 2 jours après l’injection de la 24ème semaine, un examen clinique sera effectué et un prélèvement sanguin sera réalisé pour l’analyse immunologique. Toutefois la quantité totale de sang prélevé, pour l’analyse immunologique aux visites de S24 et de S24+2jours (50ml et 50ml) sera la même que la quantité prélevée à S24 pour les sujets ne participant pas à la sous-étude (100ml), ceci pour limiter la quantité totale de sang prélevé. Le volume total prélevé sera donc de 1031 ml pour les hommes et 1041 ml pour les femmes.
- A cette visite, une biopsie cutanée sera effectuée au site même de l’injection. La biopsie, c’est à dire le prélèvement d’un morceau de peau de 4 mm de diamètre, sera effectuée sous anesthésie locale par le médecin. L’investigateur réalisera ensuite un point de suture à l’aide d’un fil. Comme ce point de suture est susceptible de laisser une cicatrice et afin de limiter ce désagrément esthétique, l’injection et la biopsie seront réalisées à la face interne du bras.

Une dernière visite est prévue 12 jours après la 24^ème^ semaine au cours de laquelle un examen clinique sera réalisé et le fil du point de suture retiré.

Une partie du sang prélevé lors de cette sous-étude sera congelée et conservée dans la biothèque (collection d’échantillons sanguins congelés) de l’étude. Ces échantillons congelés seront utilisés pour vérifier éventuellement certaines données, pour réaliser des recherches biologiques particulières dans la mesure où celles-ci permettraient de mieux comprendre la tolérance et l’immunogénicité du candidat-vaccin. Si d’autres études notamment génétiques doivent être réalisées, votre consentement sera demandé.

**Quels sont les risques ?**

La préparation vaccinale sera la même (même produit et même dose) que celle utilisée pour les trois injections précédentes, donc les risques seront les mêmes, notamment une gène au point d’injection (douleur, contracture, rougeur, gonflement, induration) et/ou l’apparition de signes généraux (fièvre, maux de tête, frissons, fatigue ou éruption). Un risque supplémentaire, lié à la biopsie, tel qu’un petit suintement sanguinolant, peut être envisagé. La réalisation d’un point de suture suite à la biopsie, entraînera la présence d’une cicatrice minime sur la face interne du bras. Toutefois, l’apparition d’une chéloïde (bourrelet dur de la peau) n’est pas exclue chez les sujets présentant une tendance à développer ce genre de cicatrices. Celle-ci est toujours de taille très limitée, correspondant au diamètre de la biopsie (4mm).

**Ce qu’il vous est demandé?**

Il est impératif que vous vous protégiez vis à vis de tout risque de contamination, notamment sexuelle. Vous ne serez en effet pas protégé contre le VIH par le candidat-vaccin.

Cette sous-étude reste dans le cadre de l’essai ANRS VAC16 qui est sans bénéfice individuel direct pour vous, puisque aucun avantage médical résultant de votre participation ne peut vous être garanti.

Il est demandé à toute femme participant à cette sous-étude et susceptible d’avoir un enfant de s’assurer d’une contraception efficace, les effets de LPVIH1 étant inconnus sur la grossesse.

Pendant la période de cette sous-étude et 4 mois après la fin de cette sous-étude (période d’exclusion), vous ne pourrez participer à aucune autre recherche biomédicale. Il vous sera également demandé de ne pas donner votre sang ni de tissu humain (moelle, organes…) durant l’essai puis pendant la période d’exclusion et/ou négativation du test ELISA.

**Quels sont vos droits ?**

Vous êtes entièrement libre d'accepter ou de refuser de participer à cette sous-étude, cela ne remettra pas en cause votre participation à l’étude elle-même et au réseau des volontaires de l’ANRS. Vous êtes entièrement libre d'arrêter votre participation à l'étude et ce, à tout moment . Dans ce cas, il vous sera demandé de prévenir l'un des médecins précédemment rencontré.

Nous vous avertirons si de nouvelles informations pouvaient vous amener à remettre en cause votre participation dans cet essai.

Pour tout renseignement, vous pouvez joindre, soit par l’intermédiaire du répondeur, soit directement l’un des médecins chargé de cette étude.

A l’issue de la recherche, si vous le souhaitez, une note d’information sur les résultats globaux de l’essai vous sera fournie.

**Confidentialité**

Toutes les données recueillies dans le cadre de cette étude resteront strictement confidentielles. Vos noms et adresse ne seront connus que de votre médecin.

Compte tenu de la nécessité de la recherche et de son analyse ultérieure, les données recueillies vous concernant feront l’objet d’un traitement informatisé et anonyme au niveau de la société prestataire chargée de cette fonction et mandatée par l’ANRS.

La loi « Informatique et Libertés » du 6 janvier 1978 (article 40) prévoit votre droit d’opposition à l’informatisation de ces données ainsi que votre droit d’accès à ces informations et de rectification de celles-ci pendant la durée de l’essai.

Ces droits pourront s’exercer auprès du Dr…………………..

Les informations qui feront l’objet de ce traitement informatisé seront les suivantes : date de naissance, sexe, événements cliniques, résultats biologiques, immunologiques et virologiques, traitements reçus et en cours.

**Informations complémentaires**

*Recherche auprès de la mairie de naissance d'un sujet perdu de vue*

Il est d'usage dans un essai clinique d'apporter la preuve qu'une personne, incluse dans un essai et avec qui le contact a été perdu en cours d'essai, n'est pas décédée. Pour cela, le médecin responsable de l'essai écrit à la mairie de naissance de cette personne pour demander cette information.

Le promoteur de cet essai, l’ANRS, fera appel à ce type de recherche d'information, si nécessaire.

*Aspects réglementaires :*

Le protocole de réalisation de cet essai et sa sous-étude a reçu l'approbation du Comité Consultatif de Protection des Personnes dans la Recherche Biomédicale de la Pitié-Salpétrière. L'essai se déroule sous la promotion de l'Agence Nationale de Recherche sur le SIDA (ANRS).

*Assurance responsabilité civile*: L’ANRS a souscrit un contrat de responsabilité civile conformément aux dispositions de l’article L.1121-7 du code de la santé publique du 20 décembre 1988, et de l’article 5 du 25 juillet 1994.

*Inscription au fichier national* : Cette étude étant sans bénéfice individuel direct, vous serez inscrit par Minitel sur le fichier national des volontaires, géré par le ministère de la Santé, sur lequel seront notées vos initiales, date de naissance, dates de début et de fin d’étude ainsi que la date de fin d’exclusion (qui est de 4 mois). Vous pourrez à tout moment vérifier l’exactitude de ces données.

*Frais engagés****:***

Les frais de transport et d’hébergement engagés pendant la période de l'essai vous seront remboursés sur justificatifs.

**CONSENTEMENT ECLAIRE DU SUJET**

Je soussigné(e) (nom, prénom) .........................................................................................

N° de sécurité sociale :...............................................

Certifie avoir lu et compris l’information indiquée ci-dessus.

Le Dr...............................................m'a proposé de participer à la sous-étude physiopathologique avec le candidat-vaccin lipopeptides du VIH (LPVIH1) pour évaluer la réponse immune locale de ce produit administré par voie intradermique, recherche organisée par l'ANRS.

J'ai eu la possibilité de poser toutes les questions que je souhaite relatives à la sous-étude, au médecin qui est chargé de mon suivi.

Je comprends les contraintes liées à ma participation.

Il m'a précisé que je suis libre d'accepter ou de refuser de participer et que cette recherche n'entraînera aucun bénéfice individuel direct pour ma santé.

Je connais la possibilité qui m'est réservée d'interrompre cette étude à tout moment sans que cela me soit préjudiciable et sans avoir à justifier ma décision mais, je m'engage à en informer le Dr ...............................................

J'accepte que les données enregistrées à l'occasion de ce protocole puissent faire l'objet d'un traitement informatisé anonyme par le promoteur ou pour son compte. Cependant des personnes mandatées par l’ANRS ou par les autorités de santé pourront avoir accès aux données qui me concernent, dans le respect le plus strict de la confidentialité, pour vérifier que l'étude s'est déroulée de manière conforme à la loi HURIET. Je note que le droit d'accès prévu par la loi "informatique et libertés" (Art. 40) s'exerce à tout moment et que je pourrai exercer mon droit de rectification et d'opposition auprès de l’ANRS par l'intermédiaire du Dr …………………

J'accepte d'être inscrit sur le fichier national des volontaires géré par le Ministère de la Santé et j’ai été informé que je peux vérifier l’exactitude des données qu’il contient auprès du titulaire de l’autorisation de lieu de recherche et leur destruction au terme du délai prévu à l’article R.2045 du code de la santé publique.

Pendant la durée prévue de ma participation, j’accepte qu’il soit procédé à une enquête auprès de ma mairie de naissance dans le cas où je cesserais de participer à cette recherche sans avertir l’un des médecins responsables de l’essai et sans répondre aux demandes de renseignements sur mon état de santé qui me seraient adressées

Je m'engage à ne pas participer à une autre recherche biomédicale sans bénéfice individuel direct pendant la durée de la recherche. La durée d'exclusion pour la participation à une autre recherche sans bénéfice individuel direct est de 4 mois après la fin d'étude

J'aurai connaissance des résultats des examens médicaux pratiqués durant l'essai.

Cette étude a reçu un avis favorable du Comité Consultatif de Protection des Personnes dans la Recherche Biomédicale de la Pitié-Salpétrière le 18 / 06 / 2003.

L’ANRS, promoteur de cette étude, a souscrit un contrat d'assurance de responsabilité civile qui me protège des risques encourus par l'essai, conformément à la loi HURIET (art. L1121-7 du Code de la Santé Publique).

J'accepte de participer à cette étude dans les conditions précisées ci-dessus.

Mon consentement ne décharge en rien les organisateurs de l'étude de leurs responsabilités, je conserve tous mes droits garantis par la loi HURIET du 20/12/88, modifiée le 24/07/94.

Fait à ........................................le...................................... Signature

Je soussigné (e), Docteur....................................., certifie avoir communiqué toute information utile concernant cette étude. Je m'engage à faire respecter les termes de cette note de consentement, conciliant le respect des droits et des libertés individuelles et les exigences d'un travail scientifique.

Fait à ........................................le...................................... Signature

Fait en 3 exemplaires :1 exemplaire pour le volontaire, 1 pour le médecin et 1 pour l’ANRS (ce dernier sera mis sous enveloppe scellée pour préserver la confidentialité)

Annexe 4

Cotation des événements indésirables

(échelle ANRS,version 5 du 11-06-02)

Annexe 5

Fiche de déclaration d’événement indésirable

Annexe 6

Fiche standard de recueil des données initiales/ finales

concernant la grossesse

(version du 23/09/97)

Annexe 7

Centres et personnel de l’essai

**I-Coordination et Gestion de l’essai**

**Coordination scientifique**

⮱ Pr Dominique SALMON-CERON

🕿 : 01.58.41.21.29, Fax : 01.43.26.88.92

E-mail : dominique.salmon@cch.ap-hop-paris.fr

**Investigateur coordonnateur**

⮱ Dr Odile LAUNAY

🕿 : 01.44.07.15.41, Fax : 01.40.46.93.08

E-mail : odile.launay@cch.ap-hop-paris.fr

**Co-investigateur**

⮱Dr. Benjamin Silbermann

🕿 : 01.43 25 38 67, Fax : 01.40.46.93.08

E-mail : benjamin.silbermann@cch.ap-hop-paris.fr

**Chef de projet**

⮱ Dr Corinne DESAINT

🕿 : 01.58 41.19.09, Fax : 01.40.46.93.08

E-mail :corinne.desaint@cch.ap-hop-paris.fr

Centre des Essais Vaccinaux Cochin-Pasteur,

Pavillon Saint Louis, Hôpital Cochin,

27, rue du faubourg Saint-Jacques, 75 679 PARIS cedex 14,

🕿 : 01.43.25.38.67, Fax : 01.40.46.93.08

**Pharmacie Coordinatrice**

⮱Dr. Corinne GUERIN

Pharmacie/Secteur des essais cliniques, Hôpital Cochin

27, rue du faubourg Saint-Jacques, 75 679 PARIS cedex 14,

🕿 : 01.58.41.23.09, Fax : 01 58 41 23 12

E-mail : corinne.guerin@cch.ap-hop-paris.fr

| **Clinical Data Management**  ⮱ Audit CRO en cours  🕿 : | **Biostatistiques**  ⮱ Christine DURIER  INSERM SC 10, Hôpital Paul Brousse  16, avenue Paul Vaillant Couturier,  94807 Villejuif  🕿 : 01 45 59 51 56, Fax : 01 45 59 51 80  E-mail : c.durier@vjf.inserm.fr |
| --- | --- |

**Responsable de la Pharmacovigilance**  **Responsable des affaires Réglementair**es

| ⮱ Dr. Annie METRO  ANRS  101 rue de Tolbiac, 75 013 Paris  🕿 : 01.53.94.60.35, Fax : 01.53.94.60.02  E-mail : annie.metro@anrs.fr | ⮱ Véronique RIEUX  ANRS  101 rue de Tolbiac, 75 013 Paris  🕿 : 01.53.94.60.15, Fax : 01.53.94.60.02  E-mail : veronique.rieux@anrs.fr |
| --- | --- |

II-Exploration de l’immunité cellulaire

⮱ Dr Jean-Gérard GUILLET

🕿: 01.40.51.65.33, Fax : 01.40.51.65.35

E-mail : guillet@cochin.inserm.f

⮱ Dr Hanne GAHERY

🕿 : 01.43.21.04.80, Fax : 01 40 48 83 52

E-mail : [:](mailto:guillet@cochin.inserm.fr) gahery@cochin.inserm.fr

Département d’immunologie de l’Institut Cochin

22, rue Méchain, 7514 Paris, France.

III-Liste des centres participant à l’essai

**1. Centre 056 : centre coordonnateur**

Service de Médecine Interne, Essais Vaccinaux, Pavillon Saint Louis,

Hôpital Cochin,

27, rue du faubourg Saint Jacques,

75 679 PARIS cedex 14,

🕿 : 01.43.25.38.67

Fax : 01 40 46 93 08

**3. Centre 054** :

Service d’immunologie clinique,

Hôpital Européen Georges Pompidou,

20, rue Leblanc, 75 908 PARIS

🕿 : 01 56 09 25 62

Fax : 01 56 09 28 59

**5. Centre 076** :

Service des Maladies Infectieuses et Tropicales,

Hôpital de Nantes,

NANTES

🕿 : 02.40.08.33.72

Fax : 02 40 08 31 81

**2. Centre 049 :**

Service des Maladies Infectieuses et Tropicales,

Hôpital Tenon,

4, rue de la Chine, 75010 PARIS.

🕿 : 01.56.01.74.13

Fax : 01 56.01.74.38

**4. Centre 072** :

Service d’Hématologie,

Hôpital Sainte Marguerite,

270, Bd Sainte Marguerite, 13 009 MARSEILLE

🕿 : 04.91.74.56.78

Fax : 04 91 74 49 62

**6. Centre 085 :**

Service des Maladies Infectieuses et Tropicales,

Hôpital Purpan,

TOULOUSE

🕿 : 05.61.77.75.34

Fax : 05 61 77 21 38

**Centre coordonnateur n°56**

Hôpital Cochin

27, rue du Faubourg Saint Jacques

75679 Paris Cedex 14

**Investigateurs**:

Dr Odile LAUNAY

Dr Benjamin SILBERMANN,

🕿 :01.44.07.15.41, Fax : 01.40.46.93.08

01.58.41.19.11

**Laboratoires des analyses de « routine »**

Centre de Transfusion Sanguine

⮱ Dr F. HESHMATI

Hôpital Cochin

🕿 : 01.58.41.86.66

Immunologie clinique

⮱Dr B. WEILL

Hôpital Cochin

🕿 : 01.58.41.20.07

Virologie

⮱ Pr LEBON

74 avenue Denfert Rochereau

75014 Paris

🕿 : 01.40.48.82.43

Pharmacologie Clinique

⮱ Pr GIROUD

Hôpital Cochin

🕿 : 01.58.41.23.20

Hématologie

⮱ Dr F. PICARD

Hôpital Cochin

🕿 : 01.58.41.09.79

Hémostase

⮱ Dr TOULON

Hôpital Cochin

🕿 : 01.58.41.19.97

Biochimie

⮱Dr CHERRUAU, Mme DESMOULINS

Hôpital Cochin

🕿 : 01.58.41.15.98

Bactériologie

⮱ Pr PHILIPPON

Hôpital Cochin

🕿 : 01.58.41.13.75

Laboratoire HLA – CTS

⮱ Dr SOREAU

Hôpital Paul Brousse

12 avenue Paul Vaillant Couturier

94800 Villejuif

🕿 : 01.45.59.35.20

Institut Fournier, bactériologie

⮱ Dr. P. SEDNAOUI

25 bd St Jacques

75014 Paris

🕿 : 01.40.78.26.03

**Pharmacie « coordinatrice »**

Pharmacie/Secteur des essais cliniques

⮱ Corinne GUERIN

Hôpital Cochin

🕿 : 01.58.41.23.09

**Centre investigateur n°049**

Service des Maladies Infectieuses et Tropicales,

Hôpital Tenon,

4 rue de la Chine, 75020 PARIS.

🕿 : 01

Fax : 01

**Investigateurs**:

Dr Gilles PIALOUX

Dr Laurence SLAMA

🕿 : 01 56 01 74 22

Fax : 01 56.01.74.18

Laboratoires des analyses de «routine »

Centre de Transfusion Sanguine

⮱ Dr Jacqueline MAURY

🕿 : 01.56.01.64.45

Immunologie clinique

⮱ Pr. Jacqueline CAPEAU

🕿 : 01.56.01.69.03/63.04

Virologie

⮱ Pr. NICOLAS (hôp.Rothschild)

🕿 : 01.50.19.34.05

Pharmacologie Clinique

⮱

🕿 :

Hématologie

⮱ Pr. GIROT

🕿 : 01.56.01.67.43

Hémostase

⮱ Dr Pr. GIROT

🕿 : 01.56.01.69.06/67.42

Biochimie

⮱ Pr. Jacqueline ETIENNE

🕿 : 01.56.01.64.88/64.43

Bactériologie

⮱ Dr. Guillaume ARLET

🕿 : 01.56.01.67.33

Laboratoire HLA – CTS

⮱

🕿 :

**Pharmacie :**

Pharmacie/Secteur des essais cliniques

⮱ Annie BECHER

🕿 : 01.56.01.63.86/64.38

Fax : 01.56.01.73.63

**Centre investigateur n°054**

Service d’immunologie clinique,

Hôpital Européen Georges Pompidou,

20 rue Leblanc, 75 908 PARIS

🕿 : 01 56 09 25 62

Fax : 01 56 09 28 59

**Investigateurs**:

Dr Gustavo GONZALEZ CANALI

Dr Philippe CASTIEL

🕿 : 01 56 09 25 62/01 56 09 32 99

Fax :01 56 09 28 59

**Laboratoires des analyses de « routine »**

Centre de Transfusion Sanguine

⮱ Dr Anne FRANCOIS

🕿 01 56 09 38 74

Immunologie

⮱ Pr FRIDMAN

🕿 : 01 56 09 39 40

Virologie

⮱ Dr Ali SI MOHAMED

🕿 : 01 56 09 39 58

Pharmacologie Clinique

⮱ Pr Jean-Michel ALEXANDRE

🕿 : 01 56 09 39 60

Hématologie

⮱ Pr Anne-Marie FISCHER

🕿 : 01 56 09 39 37

Hémostase

⮱ Pr Martine AIACH

🕿 : 01 56 09 39 01

Biochimie

⮱ Dr Nicole MOATI

🕿 : 01 56 09 39 43

Bactériologie

⮱ Pr Laurent GUTMANN

🕿 : 01 56 09 39 51

Laboratoire HLA – CTS

⮱ Dr Anne FRANCOIS

🕿 : 01 56 09 39 40

**Pharmacie**

⮱ Brigitte SABATIER

🕿 : 01 56 09 31 54

Pharmacie/Secteur des essais cliniques

🕿 : 01 56 09 25 69 / 31 54

**Centre investigateur n°072**

Service d’Hématologie,

Hôpital Sainte Marguerite,

270 Bd Sainte Marguerite, 13 009 MARSEILLE

🕿 : 04.91.74.56.78

Fax : 04 91 74 49 62

**Investigateurs**:

Dr Isabelle POIZOT-MARTIN

🕿 : 04 91 74 61 63

Fax : 04 91 74 50 69

**Laboratoires des analyses de « routine »**

Centre de Transfusion Sanguine

⮱ Dr CHABRIERES

🕿04.91.74.46.54

Immunologie clinique

⮱ Pr. BERNARD (La Timone)

🕿 : 04.91.38.39.07

Virologie

⮱ Pr ; BONGRAND

🕿 : 04.91.74.32.09

Pharmacologie Clinique

⮱ Pr ; BLIN (La Timone)

🕿 : 04.91.38.75.63

Hématologie

⮱ ⮱ Pr. PORTUGAL

🕿 : 04.91.74.40.46

Hémostase

⮱ Pr. PORTUGAL

🕿 : 04.91.74.40.46

Biochimie

⮱ Pr ; PORTUGAL

🕿 : 04.91.74.40.46

Bactériologie

⮱ Pr ; RAOULT

🕿 : 04.91.38.55.19

Laboratoire HLA

⮱ Dr ; MERCIER

🕿 : 04.91.18.95.71

**Pharmacie**

Madame PENOT-RAGON

🕿 : 04 91 74 40 45.

Fax :04 91 74 54 69

**Centre investigateur n°076**

Service des Maladies Infectieuses et Tropicales,

Hôpital de Nantes,

NANTES

🕿 : 02.40.08.33.72

Fax : 02 40 08 31 81

**Investigateurs**:

Dr Bénédicte BONNET

Dr Véronique RELIQUET

🕿 : 02 40 08 31 12/02 40 08 33 14

Fax : 02 40 08 31 81

**Laboratoires des analyses de « routine »**

Centre de Transfusion Sanguine

⮱ Dr FOLLEA

🕿 02 40 12 33 38

Immunologie clinique

⮱ Dr AUDRAIN

🕿 : 02 40 08 40 67

Virologie

⮱ Pr. BILLAUDEL

Dr FERRE

🕿 : 02 40 08 41 01

Pharmacologie Clinique

⮱ Dr KERGUERIS

🕿 : 02 40 08 40 95

Hématologie

⮱ Pr. BATAILLE

🕿 : 02 40 08 40 49

Hémostase

⮱ Dr FREISSINAUD

🕿 : 02 40 08 40 68

Biochimie

⮱ Dr ORSONNEAU

🕿 : 02 40 08 40 04

Bactériologie

⮱ Dr RICHET

🕿 : 02 40 08 39 61

Laboratoire HLA – CTS

⮱ Dr BIGNON, Dr. C. ADJOU

🕿 : 02 40 12 33 24

⮱ Dr. C. ADJOU

🕿 : 02 40 12 33 19

**Pharmacie**

Pharmacie/Secteur des essais cliniques

Isabelle FALCONI – Pr. Vincent BALLEREAU

Aline LEPELLETIER

🕿 : 02 40 08 40 42 / 02 40 08 41 53

Fax : 02 40 08 41 63

**Centre investigateur n°085**

Service des Maladies Infectieuses et Tropicales,

Hôpital Purpan,

TOULOUSE

🕿 : 05.61.77.75.34

Fax : 05 61 77 21 38

**Investigateurs**:

Pr Patrick MASSIP

Dr Lise CUZIN

Dr Martine OBADIA

🕿 : 05 61 77 90 43/05 61 77 76 18

Fax : 05 61 77 21 88

**Laboratoires des analyses de « routine »**

Centre de Transfusion Sanguine

⮱ Etablissement français du sang

🕿 05.61.31.20.10

Immunologie clinique

⮱ Dr KUCHEIN

🕿 : 05.61.32.34.31

Virologie

⮱ Pr . IZOPET

🕿 :05.61.77.22.71

Pharmacologie Clinique

⮱ Pr. MONTASTRUC

🕿 : 05.61.14.59.00

Hématologie

⮱ Pr. SIE

🕿 : 05.61.77.90.71

Hémostase

⮱ Pr. SIE

🕿 : 05.61.77.90.71

Biochimie

⮱ Pr. THOUVENOT

🕿 : 05.61.77.23.06

Bactériologie

⮱ Pr. DABERNAT

🕿 : 05.61.77.21.22

Laboratoire HLA – CTS

⮱ Dr. ABBAL

🕿 : 05.61.32.22.06

**Pharmacie**

Pharmacie/Secteur des essais cliniques

Dr. PEYRANNE

🕿 : 05.61.77.24.96

Fax :05.61.77.75.14

Annexe 8

Attestation d’assurance

Et

Autorisations de lieux
